# Examination of Coligands in Mefloquine–Metal Complexes Reveals the Structural Determinants of Activity against *Plasmodium falciparum* and *Schistosoma mansoni*

**DOI:** 10.1021/acs.jmedchem.5c03739

**Published:** 2026-03-11

**Authors:** Wilmer Villarreal, Helenita Costa Quadros, Legna Colina-Vegas, Sammy Y. Aboagye, Godwin Akpeko Dziwornu, Gabriel H. Ribeiro, Ariane Isis Barros, Dawid Jakub Kucharski, Mahsa Rahbari, Christina Brandstädter, Sarah D’Alessandro, Nicoletta Basilico, Keabetswe Masike, Nandi Mehlala, Joaquim Araújo Nobrega, Victor M. Deflon, Maribel Navarro, Przemysław J. Boratyński, Kelly Chibale, David L. Williams, Alzir A. Batista, Diogo R. M. Moreira

**Affiliations:** † 201361 Universidade Federal de São Carlos, Departamento de Química, São Carlos, SP 13565-905, Brazil; ‡ Universidade Federal do Rio Grande do Sul, Instituto de Química, Porto Alegre, RS 91501-970, Brazil; § Fundação Oswaldo Cruz, Instituto Gonçalo Moniz, Salvador, BA 40296-710, Brazil; ∥ 2468 Rush University Medical Center, Department of Microbial Pathogens and Immunity, Chicago, Illinois 60612, United States of America; ⊥ Drug Discovery and Development Centre (H3D), Department of Chemistry, 37716 University of Cape Town, Rondebosch 7701, South Africa; # 67828 Universidade Federal de Mato Grosso, Departamento de Solos e Engenharia Rural, Cuiabá, MT 78060-900, Brazil; g Department of Organic and Medicinal Chemistry, Wrocław University of Technology, Wyb. Wyspiańskiego 26, Wrocław 50-370, Poland; h Biochemistry and Molecular Biology, Interdisciplinary Research Center, 9175 Justus Liebig University Giessen, Heinrich-Buff-Ring 26-32, Giessen, 35392, Germany; i Dipartimento di Scienze Farmacologiche e Biomolecolari, 9304 Università degli Studi di Milano, Milan, 20133, Italy; j Dipartimento di Scienze Biomediche, Chirurgiche e Odontoiatriche, Universitá degli Studi di Milano, Milan, 20133, Italy; k South African Medical Research Council Drug Discovery and Development Research Unit, Department of Chemistry and Institute of Infectious Diseases and Molecular Medicine, University of Cape Town, Rondebosch 7701, South Africa; l Universidade de São Paulo, Instituto de Química de São Carlos, São Carlos, SP 13560-970, Brazil; m 28113 Universidade Federal de Juiz de Fora, Departamento de Química, Juiz de Fora, MG 36036-900, Brazil

## Abstract

Mefloquine (MQ) is an important component for antiparasitic therapy. Herein, the synthesis and antiplasmodial and antischistosomal activities of MQ–metal complexes of the general formula [M­(II)­(L)­(MQ)]­PF_6_ are described. Variation of the metal center (platinum and palladium) and coligand (phosphine or bipyridine) consistently yielded MQ coordinated as a *N*,*O*-bidentate ligand. Biological evaluation against *Plasmodium falciparum* and *Schistosoma mansoni* revealed that the metal center augmented the antiparasitic property of MQ by functioning as a thioredoxin/glutathione reductase-targeting moiety, while the coligand modulated chemical reactivity and physicochemical properties. MQ–Pt complexes displayed high *in vivo* efficacy. The intracellular accumulation of the metal in parasite cells contributed to the abrogation of essential biochemical pathways. Notably, despite being isostructural, Pd complexes differed from their Pt counterparts in their ligand dissociation behavior. The current work establishes a new structural framework for developing metal-based antiparasitic agents capable of selectively targeting essential parasite biochemical pathways while sparing mammalian cells.

## Graphical abstract

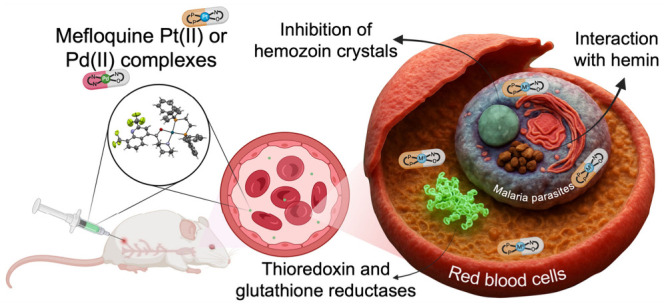

## Introduction

Parasitic diseases such as malaria (caused by *Plasmodium* spp. protozoans) and schistosomiasis (caused by *Schistosoma* spp. flatworms) are some of the most prevalent tropical diseases worldwide. Although recent control efforts have significantly reduced morbidity and mortality in many regions, major challenges persist in endemic areas. These include the decreased efficacy of malaria treatment due to the selection for parasite resistance and the rising concern of parasite resistance to Praziquantel (PZQ), the mainstay of schistosomiasis treatment, which lacking efficacy against juvenile stages warrant the urgent search for new drugs against the two diseases.^1–4^

Both *Plasmodium* and *Schistosoma* parasites are susceptible to the (2-)­piperidinyl methanol-derived quinoline drug mefloquine (MQ). Clinically, MQ is used for malaria prophylaxis and treatment, and of proven *in vitro* and *in vivo* efficacy against *S. mansoni*.^5–9^ The mode of action (MoA) for MQ remains poorly defined in schistosomiasis, whereas in malaria MQ is recognized as a drug with pleiotropic effects. MQ has been shown to bind to the 80S ribosome subunit and to *P. falciparum* purine nucleoside phosphorylase thereby inhibiting protein synthesis and purine catabolism, respectively.^10,11^ Additionally, MQ inhibits heme detoxification into hemozoin, although less effectively than chloroquine (CQ).^12,13^ Structure–activity relationships (SAR) studies indicate that MQ antiparasitic activity depends on the (2-)­piperidinyl methanol moiety and its stereochemistry, while the quinoline contributes to π-π stacking interactions with molecular targets.^13–16^

Metal complexes are important assets in the pipeline for designing new anti-infective agents because they can engage molecular targets that are inaccessible to their purely organic counterparts.^17^ To address limitations with antiparasitic quinolines, including narrow spectrum of activity and emergence of resistance, quinoline based metal complexes have been developed.^18–23^ Transition metals, in particular, offer promising scaffolds for antimicrobial design. For example, Pt­(II) complexes display increased reactivity toward biologically relevant nucleophilic agents, such as glutathione (GSH) and cysteine residues in proteins, and can undergo rapid ligand exchange reactions in blood.^24–27^ Although such reactivity may limit the specificity of Pt­(II) in some contexts, this property motivated our interest in targeting the blood stages of *P. falciparum* parasites and adult *S. mansoni* worms residing in the mesenteric veins using antiparasitic Pt­(II) and Pd­(II) complexes containing MQ in their coordination spheres (Figure 1). To evaluate the capacity of Pt­(II) and Pd­(II) agents to reach blood parasites and to deliver MQ payload, an efficient synthesis of aqueous-stable, MQ–metal complexes with the general formula [M­(II)­(L)­(MQ)]­PF_6_ was developed. These metallic complexes were hypothesized to preferentially target pathogens circulating in the bloodstream, rather than host cells, and would exhibit potent antiparasitic activity by acting on molecular targets not affected by MQ alone.

**Figure 1:**
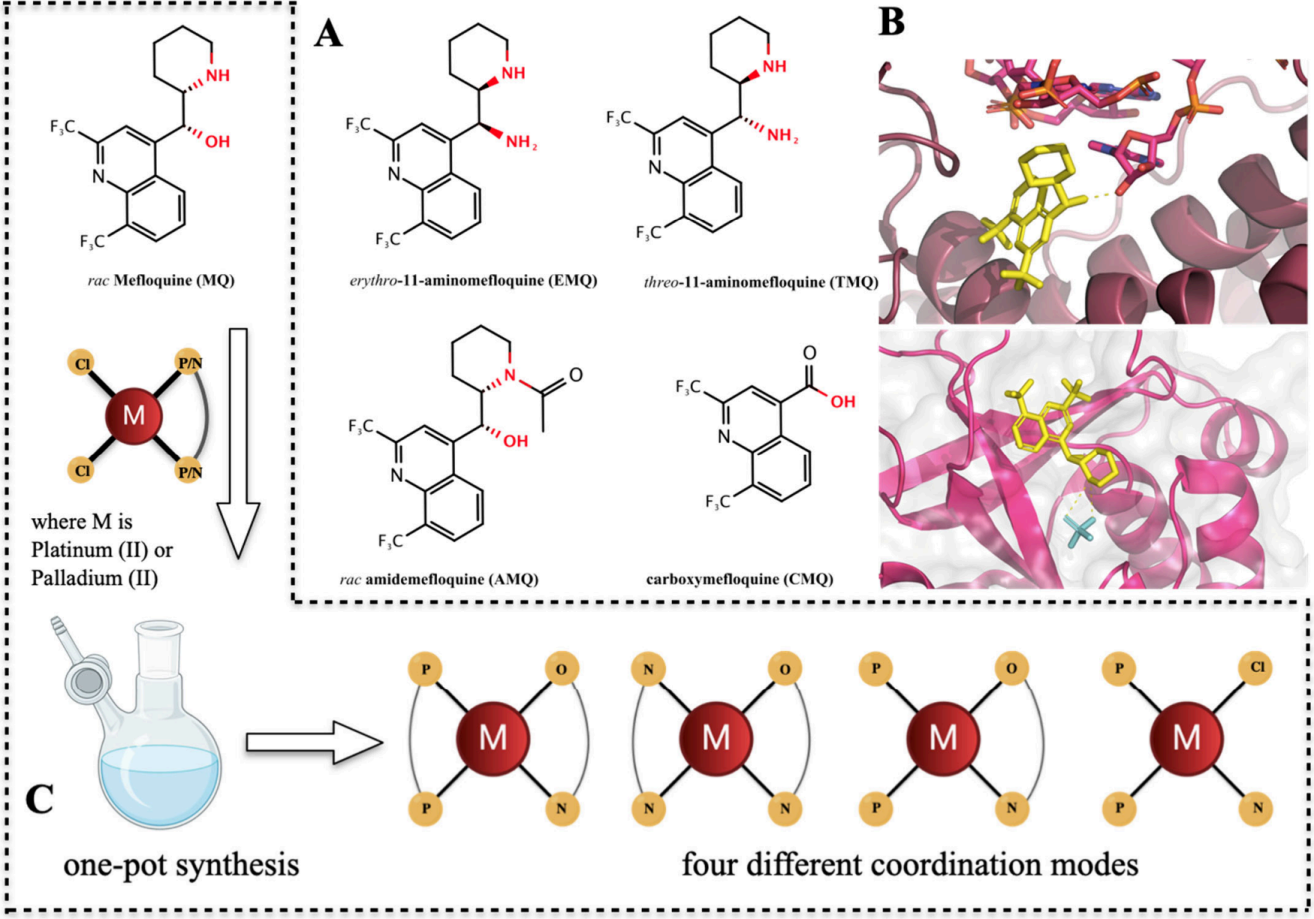
Structure–activity relationship of Mefloquine (MQ). Panel A shows the chemical structures of MQ’s aminomefloquine derivatives (EMQ and TMQ), an amide derivative (AMQ), as well as its main hepatic metabolite, carboxymefloquine (CMQ). Panel B depicts the cocrystals between MQ (in yellow) and *Plasmodium* proteins (PDB IDs 5UMD (80S ribosome), top; 5ZNI (purine nucleoside phosphorylase), bottom) highlighting the intermolecular hydrogen bonds of MQ’s (2-)­piperidinyl methanol moiety to the binding sites. The importance of the intermolecular binding properties of MQ’s (2-)­piperidinyl methanol moiety is highlighted by red labeled atoms. Panel C shows a scheme for a one-pot synthesis of MQ–metal complexes (**1**–**13**) and a quinine–metal complex (**14**).

## Results

### Chemistry

#### Synthesis and Characterization

The syntheses of mefloquine (MQ) and quinine (QN) metal complexes from a precursor of general formula [M­(L)­Cl_2_], where M = Pt­(II) and L is triphenylphosphine (PPh_3_) are presented in Figure 2. MQ coordinated to Pt­(II) as a *N*,*O*-bidentate ligand through the (2-)­piperidinyl methanol moiety, yielding complex Pt (**1**). However, like other antimalarial quinoline-based drugs such as chloroquine (CQ) and amodiaquine (AQ),^18–20^ QN coordinated as a monodentate ligand to Pt­(II) species through its quinoline nitrogen atom, yielding complex Pt (**14**).

**Figure 2:**
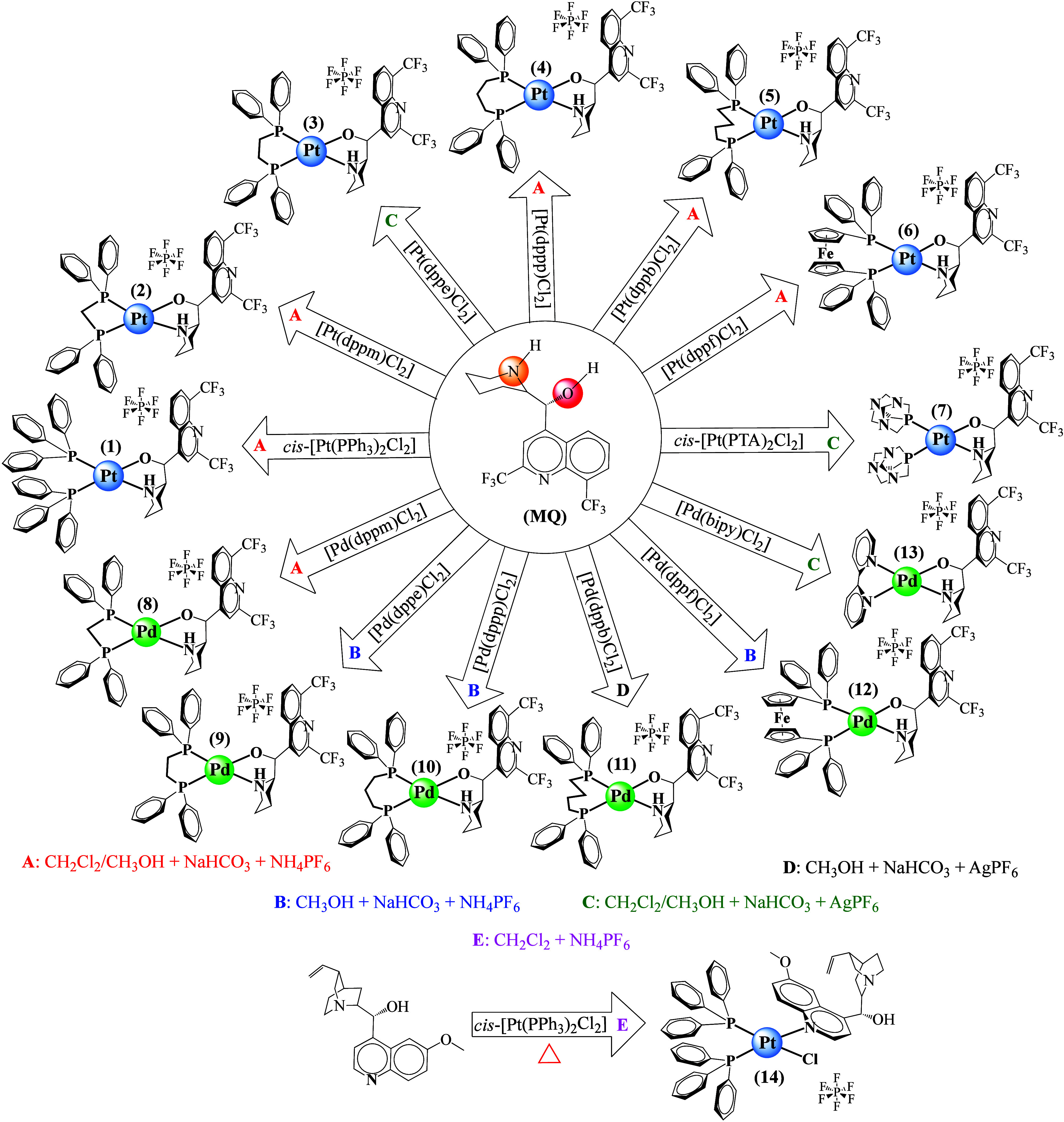
Mefloquine (MQ) reacts with Pt and Pd species as a *N*,*O*-bidentate ligand. Structures of MQ–metal complexes of general formula [M­(II)­(L)­(MQ)]­PF_6_ (**1**–**13**) and their reaction conditions. A: CH_2_Cl_2_/CH_3_OH + NaHCO_3_ + NH_4_PF_6_, B: CH_3_OH + NaHCO_3_ + NH_4_PF_6_, C: CH_2_Cl_2_/CH_3_OH + NaHCO_3_ + AgPF_6_, D: CH_3_OH + NaHCO_3_ + AgPF_6_, and E: CH_2_Cl_2_ + NH_4_PF_6_ + heat. Abbreviations for the coligands: L = triphenylphosphine (PPh_3_) (**1**, **14**), 1,1-bis­(diphenylphosphine) methane (dppm) (**2**, **8**), 1,2-bis­(diphenylphosphine) ethane (dppe) (**3**, **9**), 1,3-bis­(diphenylphosphine) propane (dppp) (**4**, **10**), 1,4-bis­(diphenylphosphine) butane (dppb) (**5**, **11**), 1,1′-bis­(diphenylphosphine) ferrocene (dppf) (**6**, **12**), 1,3,5-triaza-7-phosphoadamantane (PTA) (**7**), and 2,2′-bipyridine (bipy) (**13**). The scheme at the bottom shows that quinine reacts with Pt species as a *N*-monodentate ligand, yielding *cis*-[Pt­(PPh_3_)_2_(quinine)­Cl]­PF_6_ (**14**).

The complex Pt (**1**), showing unique coordination sphere and outstanding stability, inspired expansion of the scope of the synthesis to obtain 13 new complexes with the general formula [M­(II)­(L)­(MQ)]­PF_6_ (**1**–**13**), where M = Pt­(II) or Pd­(II) centers and L = phosphine or bipyridine as coligands (Figure 2). The inorganic base NaHCO_3_ deprotonated the hydroxy group of MQ and the addition of AgPF_6_ forced the removal of the chlorido ligand of the coordination sphere in some complexes.

In all complexes, PF_6_^–^ was employed as the counteranion necessary to stabilize the cationic species. The recorded molar conductivity values were in the range for 1:1 electrolyte in DMSO.^28^ All complexes were purified by trituration and isolated in good to excellent yields (>83%). Elemental analyses supported the proposed molecular formula and desired purity (>95%). The simple and efficient one-pot synthesis protocol described above maintained the stereochemistry of the MQ ligand, which was confirmed by NMR spectroscopy and X-ray crystallography. However, complexes of Pt with bipyridine as well as complexes of Pd with 1,3,5-triaza-7-phosphoadamantane (PTA) as coligands could not be obtained under similar conditions. A detailed description of the physical-chemical and spectral characterization of the metal complexes [M­(II)­(L)­(MQ)]­PF_6_ (**1**–**13**) are provided in the Supporting Information (Figures S1–S4, Tables S1 and S2 and associated text).

It is noteworthy that the crystal structures of complexes Pt (**2**), Pt (**6**) and Pd (**9**) confirmed a distorted planar square geometry, where the bidentate coordination of MQ to the metal centers is through the nitrogen atom of the piperidine ring and the oxygen atom of the secondary alcohol. Complexes Pt (**2**), Pt (**6**) and Pd (**9**) crystallized in the space group *P*2_1_/*c*, *P*2_1_2_1_2_1_ and *P*1̅, respectively (Figure 3A), but only the crystal structure of the complex Pd (**9**) showed the presence of the *rac*-MQ ligand (*S*,*R* and *R*,*S*) coordinated to the metal center (Figure 3B). Inspection of the overlay of the two configurations of complex Pd (**9**) presented in the crystal structure showed configurational changes (Figure 3C). These changes were more pronounced in the piperidine ring of the MQ ligand. In support of this observation, the ^13^C­{^1^H} NMR spectra not only confirmed the presence of all ligand carbons as well as the deshielding effect of the atoms near the coordination sites, such as the C3′, C1′ and C1″ atoms, but also these spectra surprisingly showed a duplication of the signals of the carbon atoms of the piperidine ring of the MQ (Figure 3D). We attributed this phenomenon to the presence of two conformational isomers produced by the combination of three factors: the enantiomers of mefloquine ligand (*S*,*R* and *R*,*S*), the chiral center formed on the nitrogen atom (a new stereogenic center generated by metal coordination) and the chair flip of the piperidine ring (Figure 3E).

**Figure 3:**
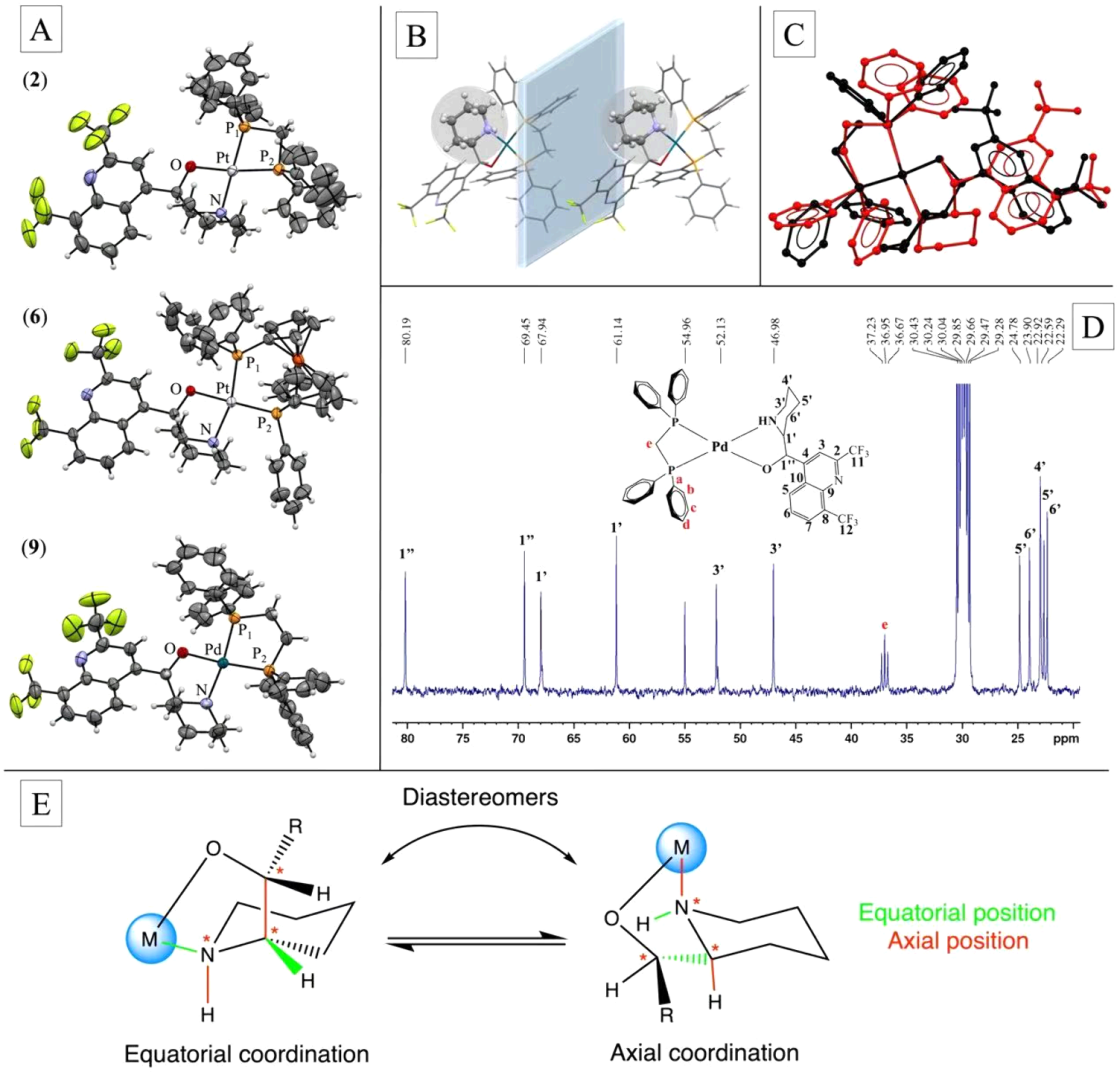
Metals form two diastereomeric complexes with the *rac*-MQ ligand (*S*,*R* and *R*,*S*). Panel A) The crystal structures of the complexes Pt (**2**), Pt (**6**) and Pd (**9**) at 50% of thermal ellipsoids. For clarity, the presence of the counterion PF_6_^–^ and solvents were omitted. Panel B) The crystal structure of complex Pd (**9**), highlighting the orientation of the piperidine ring in the MQ ligand. Panel C) A superposition of both isomers of complex Pd (**9**) inferred from the crystal unit. Panel D) A representative ^13^C­{^1^H} NMR spectrum in the aliphatic region for complex Pd (**8**) recorded in acetone*-d*_6_, showing the duplication of the carbon atoms of the piperidine ring of MQ. Panel E) A general scheme of the conformers generated by the chair flip of the piperidine ring.

#### Chemical Stability

Monitoring the chemical stability in solution by NMR for complexes Pt (**3**), Pd (**9**) and Pt (**14**) revealed stability for up to 72 h in pure DMSO-*d*_6_. Similarly, in a mixture of DMSO-*d*_6_/D_2_O or DMSO-*d*_6_/cell culture medium (Figures S5 and S6), complex Pt (**3**) did not display any measurable chemical transformation for up to 72 h. However, complex Pd (**9**) underwent a 10% chemical transformation in a mixture of DMSO-*d*_6_/D_2_O and a 44% in chemical transformation in a mixture of DMSO-*d*_6_/cell culture medium. Its parental counterpart, complex Pt (**14**), which contains a monodentate QN ligand, underwent 23% chemical transformation in a mixture of DMSO-*d*_6_/cell culture medium. Comparatively, the Pd complexes were more reactive to ligand scrambling than their Pt counterparts. Interestingly, a Pt complex containing a monodentate QN ligand, Pt (**14**), was less reactive than the Pd complexes. The ^1^H and ^31^P­{^1^H} NMR signals revealed that the chemical transformation in solution for the metal complexes Pd (**9**) and Pt (**14**) involves the dissociation of the quinoline drug [MQ for Pd (**9**) and QN for Pt (**14**)] rather than the phosphine ligand (Figures S5, S6, S11, and S12).

### Pharmacology

#### Structure–Activity Relationship Findings

The *in vitro* activity of the MQ derivatives and metal complexes (**1**–**14**) was evaluated against asexual blood stage (ABS) of *P. falciparum*, while mammalian cell toxicity was assessed in J774 murine macrophages (Tables 1 and 2, Figures S9 and S10).^3^ The drug stability of the compounds in the presence of GSH and aqueous solubility in pH 7.4 PBS are provided in Table S3.

**Table 1:**
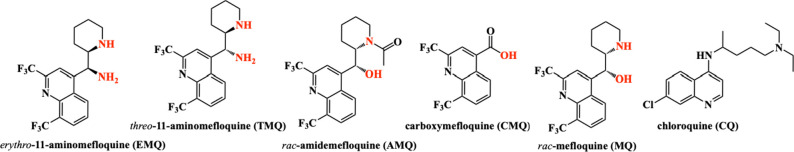
Screening of the Effects of MQ Derivatives on the Growth of Asexual Blood Stages (ABS) of *P. falciparum*, Cytotoxicity to Mammal Cells, Hemin Association Constant (log *K*) and β-Hematin Inhibitory Activity (BHIA)^a^

	*P. falciparum*, IC_50_ [nM]^a^				
Compounds	3D7	W2	J774 cells, CC_50_ [μM]^b^	Log *K* ^c^	BHIA^d^
EMQ	466 (340–522)	304 (281–329)*	29.0 (23.7–31.5)	4.11 ± 0.20	>2.0
TMQ	341 (298–342)	276 (271–288)*	46.6 (40.3–55.9)	4.20 ± 0.14	>2.0
AMQ	>1000	>1000	>100	4.08 ± 0.05	>2.0
CMQ	>1000	>1000	58.0 (48.8–119)	4.45 ± 0.15	1.87 ± 0.09
MQ	22 (16–29)	6.5 (5.8–7.2)*	10.6 (8.50–17.9)	4.51 ± 0.17	1.92 ± 0.02
CQ	10 (6–11)	319 (270–399)*	70 ± 5.5^d^	5.21 ± 0.13	0.40 ± 0.02

a Abbreviations: MQ = Mefloquine, CQ = Chloroquine. *p < 0.05 by unpaired and nonparametric Mann–Whitney rank test versus 3D7 strain.

b Assessment of parasite growth at ring stages after 72 h incubation by [^3^H]-hypoxanthine incorporation. 3D7 is a drug-sensitive strain; W2 is resistant to CQ.

c Cytotoxicity in J774 macrophage cell lineage determined after 72 h incubation by Alamar Blue. Data are the mean and confidence interval 95% of one single experiment using three technical replicates.

d Association constant (log *K*) to [Fe­(III)-PPIX] (hemin). Values are median ± S.E.M. of three independent experiments.

e β-Hematin inhibitory activity (BHIA) upon incubation with compounds and determined after 48 h. IC_50_ values (mM) are median ± S.E.M. of three independent experiments.

**Table 2:** Screening of the Metal Complexes with MQ (**1**–**13**), QN (**14**) and Their Representative Metallic Precursors on the Asexual Blood Stage Growth of *P. falciparum*, Cytotoxicity in Mammal Cells and Selectivity Indexes[Table-fn t2fn0]

	*P. falciparum*, IC_50_ ± S.E.M. [nM]^a^			Selectivity index^c^	
Compounds (coligand)	3D7	W2	Cytotoxicity in J774 CC_50_ ± S.E.M. [nM]^b^	3D7	W2
Mefloquine–Platinum Complexes					
Pt (**1**) (PPh_3_)	34 ± 4.5	10 ± 1.2	4400 ± 900	129	440
Pt (**2**) (dppm)	64 ± 6.5	72 ± 8.5	6300 ± 700	98	87
Pt (**3**) (dppe)	7.3 ± 1.5	2.1 ± 0.2	6900 ± 900	945	3285
Pt (**4**) (dppp)	18 ± 4.5	13 ± 2.4	2100 ± 1100	116	161
Pt (**5**) (dppb)	29 ± 2.7	10 ± 0.5	3100 ± 1500	106	310
Pt (**6**) (dppf)	16 ± 2.4	12 ± 1.5	6100 ± 800	381	508
Pt (**7**) (PTA)	27 ± 0.5	3.8 ± 0.4	7100 ± 300	262	1868
Mefloquine–Palladium Complexes					
Pd (**8**) (dppm)	18 ± 2.2	5.6 ± 0.9	19100 ± 1200	1061	3410
Pd (**9**) (dppe)	28 ± 5.0	3.4 ± 0.4	14900 ± 800	532	4382
Pd (**10**) (dppp)	18 ± 4.2	6.9 ± 0.6	10200 ± 900	566	1478
Pd (**11**) (dppb)	19 ± 2.3	1.6 ± 0.5	12100 ± 1000	639	7562
Pd (**12**) (dppf)	16 ± 2.2	15 ± 0.1	7100 ± 400	443	473
Pd (**13**) (bipy)	18 ± 3.9	10 ± 1.1	20900 ± 2800	1161	2090
Quinine–Platinum Complex					
Pt (**14**) (PPh_3_)	54.9 ± 3.8	266 ± 15.7	22900 ± 3800	417	86
Metal Precursors					
Pt (**15**) (dppe)	>2000	N.D.	75500 ± 7500	N.D.	N.D.
Pd (**16**) (dppe)	>2000	N.D.	35100 ± 4000	N.D.	N.D.
Antiplasmodials of Reference					
MQ	15 ± 0.6	3.5 ± 0.4	11600 ± 1200	773	3314
QN	131 ± 20.0	457.1 ± 22.5	25400 ± 2200	193	55
CQ	10 ± 1.2	319 ± 27	70000 ± 5500	7000	219

a The data in columns 2–4 are the mean ± S.E.M. of three independent experiments, each concentration in triplicate. Chemical structures of all compounds are shown in Figure 2 and in the Supporting Information. Abbreviations: S.E.M. = standard error of the median. MQ = Mefloquine. CQ = Chloroquine. QN = Quinine.

b Rings stage parasites were incubated with the drugs for 72 h. Parasite growth was assessed by [^3^H]-hypoxanthine incorporation. 3D7 is a drug-susceptible strain; W2 is resistant to chloroquine.

c Cytotoxicity in J774 macrophage cell lineage determined after 72 h incubation and viability assessed by Alamarblue.

d Selectivity indexes were calculated as CC_50_/IC_50_.

The cocrystal structures between MQ and *Plasmodium* proteins indicate that MQ establishes intermolecular interactions through H-bonding of the secondary alcohol at position 1″ and hydrophobic interactions with the piperidine ring (Figure 1), where the stereochemistry and the conformational dynamics of this ring further modulate the overall affinity of MQ for binding to its targets.^9,10,29^ In this regard, the amino-derivatives of MQ, diastereomers EMQ and TMQ, were screened for their antiplasmodial activity and metal binding property of MQ to investigate the effect of the amino group as potential isosteric replacement of the secondary alcohol at position 1″ of the MQ (Table 1).^30^ These amino-derivatives of MQ showed a 40-fold decrease in antiplasmodial potency and a slightly reduced affinity for binding to hemin compared to MQ. Attempts to prepare Pt complexes with TMQ under the same condition used for Pt (**1**) did not yield the desired Pt complex containing the TMQ as a ligand, suggesting that the secondary alcohol at position 1″ of MQ is essential for metal binding property and for the antiplasmodial activity of MQ. Similarly, the acetamide derivative of MQ (AMQ) lacked antiplasmodial activity and hemin-binding properties. In contrast, CMQ, a metabolite of MQ lacking the (2-)­piperidinyl methanol moiety, exhibited affinity for binding to hemin, consistent with the established role of the quinoline ring in this interaction. However, like previous reports,^31,32^ CMQ did not display antiplasmodial activity.

With regards to the SAR of the metal complexes (**1**–**13**) (Table 2), both the monodentate phosphine Pt (**1**) and bidentate phosphine (dppm) containing Pt (**2**) exhibited *in vitro* antiplasmodial potencies lower than that of MQ, which could be attributed to a rapid ligand exchange reaction in the presence of GSH and a 4-member ring between phosphine and metal in Pt (**2**). Unlike Pt (**2**), the bidentate phosphine in complexes Pt (**3**–**6**) yielded *in vitro* activities comparable to or greater than MQ. Despite its desirable *in vitro* potency, complex Pt (**7**) containing a monodentate phosphine (PTA) showed the least aqueous stability (Table S3), followed by complex Pt (**5**), containing a hydrophobic bidentate phosphine (dppb).

For Pd (**8**–**12**), potencies mirrored those observed for Pt counterparts (**2**–**6**), with Pd complexes slightly less potent than the Pt analogs. Complexes Pd (**8**–**12**) displayed ligand exchange reactions with GSH faster than Pt complexes. Notably, Pd complexes displayed lower aqueous solubility than their Pt counterparts. Although the poor solubility of these complexes in the mid-μM range may not fully account for their lower antiplasmodial potency in the nM range, it could explain their comparatively reduced cytotoxicity toward J774 cells relative to the Pt complexes. It is worth noting that complex Pd (**13**), which contains a bipyridine ligand, exhibited improved solubility compared to that of the Pt compounds. However, it was less potent against drug-resistant parasites (W2 and TM91C235 strains), indicating a shift in the phenotypic activity profile.

The quinoline ring of MQ, its derivatives and the complexes (**1**–**13**) played an important role in hemin binding and in the inhibition of β-hematin formation (Table 1, Table S4). However, this property did not correlate with the *in vitro* antiplasmodial activity mediated through suppression of the heme detoxification pathway, an observation consistent with previous studies.^11,14,15^ Moreover, mammalian cell toxicity for complex Pt (**3**) was similar across the different cell lines tested (Table S5), supporting its favorable selectivity profile. Based on the overall SAR analysis, complex Pt (**3**) emerged as the benchmark compound for subsequent *in vitro* and *in vivo* studies.

#### Spectrum of Activity against *P. falciparum*

To characterize the antiplasmodial spectrum of the frontrunner complexes Pt (**3**) and congener Pd (**9**), their activity was first validated in asynchronous culture against the ABS of two additional *P. falciparum* strains. Consistent with the differences observed between 3D7 and W2 strains, MQ and both complexes showed higher potency against K1 (multidrug-/CQ-resistant) than against NF54 (CQ-susceptible) strains (Table S6). This observation aligns with the phenomenon of collateral drug sensitivity, in which parasite strains such as W2 and K1, each carrying a single copy of the *pfmdr1* gene, often display higher susceptibility to MQ.^29,33–35^ In all these parasite strains, Pt (**3**) had IC_50_ values lower than MQ, confirming its frontrunner status (Table S6).

Gametocytes, the parasite stages responsible for human-to-insect transmission,^36^ are known to be susceptible to the redox-active drug methylene blue (MB). Given the potential of Pt and Pd complexes to affect enzymes involved in the thiol redox homeostasis, we evaluated their activity against stages IV/V *P. falciparum* gametocytes (Table S6). Although gametocytes were sensitive to MB, their viability was not affected by MQ or its metal complexes.

Another characteristic of MQ is that its inhibition of the ABS of *P. falciparum* occurs more slowly than CQ or dihydroartemisinin (DHA) but more rapidly than Atovaquone (ATO). This pattern reflects the fact that MQ primarily inhibits trophozoites stages,^37^ whereas DHA rapidly suppresses parasite growth within 24 h by targeting both rings and trophozoites stages. MQ and its metal complexes exhibited a similar onset of action against parasite growth reducing parasite viability after 48 h of incubation, while ATO required approximately 72 h to achieve a comparable effect (Table S6).

The IC_50_ values obtained against the MQ-resistant TM91C235 strain of *P. falciparum* (Figure 4A) showed a marked loss of potency for MQ and its complexes compared with the MQ-susceptible W2 strain. In contrast, DHA showed no significant change in IC_50_ between the two strains (Figure 4B). Both increased copy number and single nucleotide polymorphisms on *pfmdr1* can reduce the transport of drugs from the cytosol into the digestive vacuole.^34,35^ To evaluate whether overcoming this impaired drug transport alters parasite survival, the parasites were exposed to a drug concentration approximating MQ plasma levels and monitored for recrudescence (Figure 4C). In the drug susceptible NF54 strain, no recrudescence was observed following treatment with MQ or Pt (**3**), whereas ATO at subtherapeutic concentration permitted recrudescence. The W2 strain, which carries a single copy of the *pfmdr1* gene, showed rapid recrudescence after CQ treatment. In contrast, both MQ and Pt (**3**) significantly delayed recrudesce relative to CQ. For the TM91C235 strain, which contains multiple *pfmdr1* copies, recrudescence occurred 7-days later with Pt (**3**) than with MQ, and this delay was statistically significant. Together, these data show that once drug transport mediated resistance is bypassed, complex Pt (**3**) can inhibit additional parasitic targets in the parasite life cycle not affected by MQ, which explains its superior efficiency against parasites harboring multiple *pfmdr1* copies.

**Figure 4:**
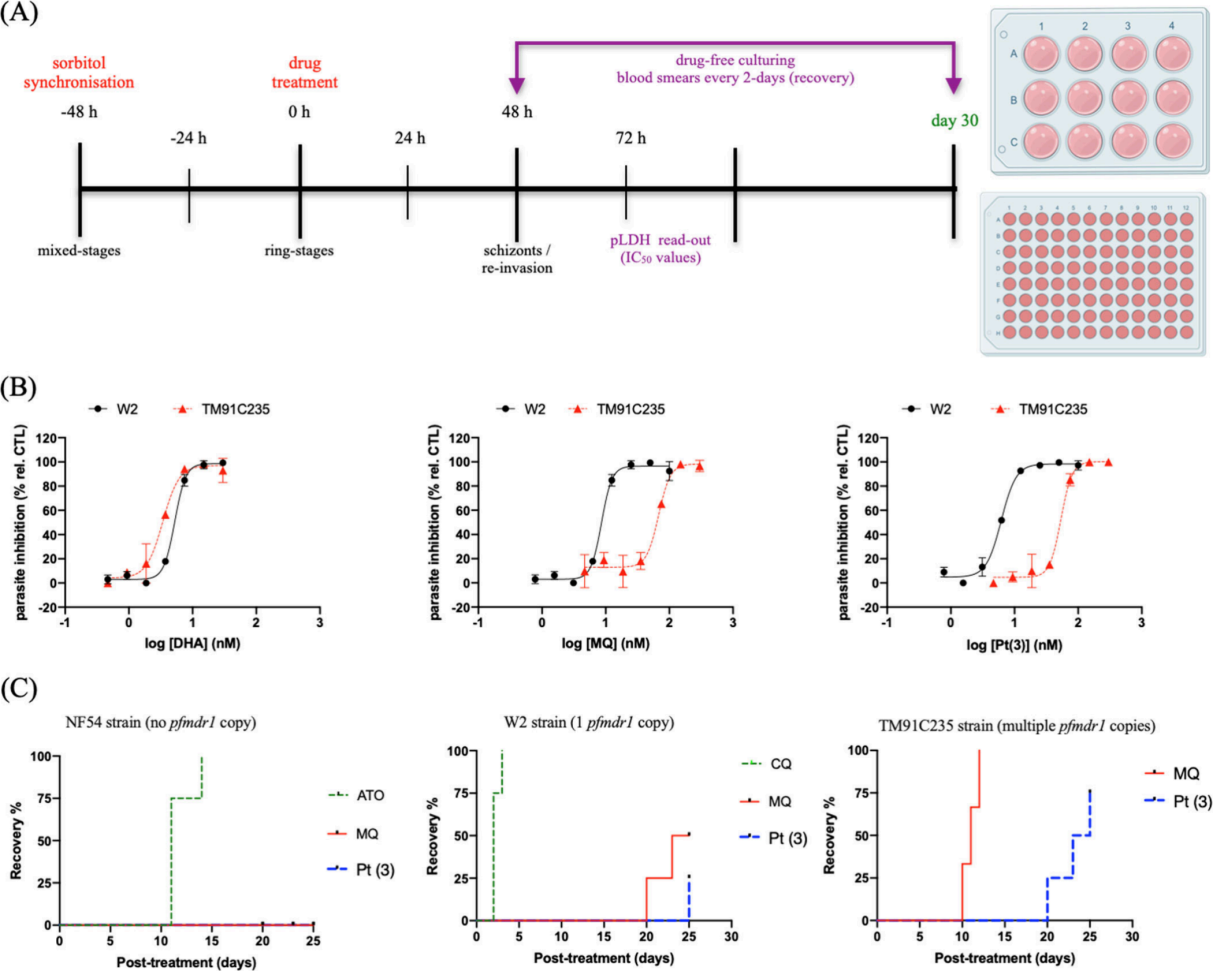
Examination of the antiplasmodial activity for Pt (**3**) across a panel of different parasite strains of *P. falciparum*. Panel A shows the experimental design. Panel B shows representative curves of the parasite inhibition in MQ-susceptible (W2 strain) and MQ-resistant (TM91C235 strain) after 72 h incubation and determined by pLDH readout. Points are the mean ± SD of two independent experiments, which one containing two replicates. Panel C shows the survival profiles of parasites after treatment with a single concentration of drugs MQ or Pt (**3**) at 1000 nM, CQ at 800 nM and ATO at 1000 nM and determined by microscopy. The percentage of parasitemia recovered to the onset of the experiment (recovery %) was determined in four technical replicates. The IC_50_ values, recrudescence times to recover and the statistical analysis are shown in Table S6 in the Supporting Information. Abbreviations: CQ = chloroquine; DHA = dihydroartemisinin; ATO = atovaquone.

### Mechanism of Action

#### Antiplasmodial Activity

Like MQ, both metal complexes Pt (**3**) and Pd (**9**) exhibited more potent activity against *P. falciparum* during the trophozoites stages, whereas the control drug DHA inhibited both the rings and trophozoites stages (Figure 5A). This stage-specific activity aligns with the peak in hemozoin (Hz) formation, protein synthesis and purine catabolism that occur during the trophozoites stages, processes that are known to target MQ. To determine whether Pt (**3**) differs from MQ on its effect on the heme detoxification pathway involved in the formation of Hz crystals, their activities were compared using ultrastructural transmission electron microscopy (TEM). Trophozoites treated with a 50 nM Pt (**3**) displayed fewer hemozoin (Hz) crystals than those treated with MQ (Figure 5B). Figure 5C shows the quantification of the hemin binding affinity of all compounds and their ability to inhibit β-hematin crystal formation. Across complexes (**1**–**13**), metal coordination significantly increased the hemin binding affinity (log *K*) and the inhibition of β-hematin crystallization (IC_50_) relative to MQ. Specifically, Pt (**3**) displayed a mean log *K* of 4.83 ± 0.05 (versus 4.51 ± 0.17 for MQ) and inhibited β-hematin formation with a mean IC_50_ of 0.57 ± 0.02 mM (versus 1.92 ± 0.02 for MQ), albeit less potently than CQ (mean 0.40 ± 0.02 mM) (Table S4).

**Figure 5:**
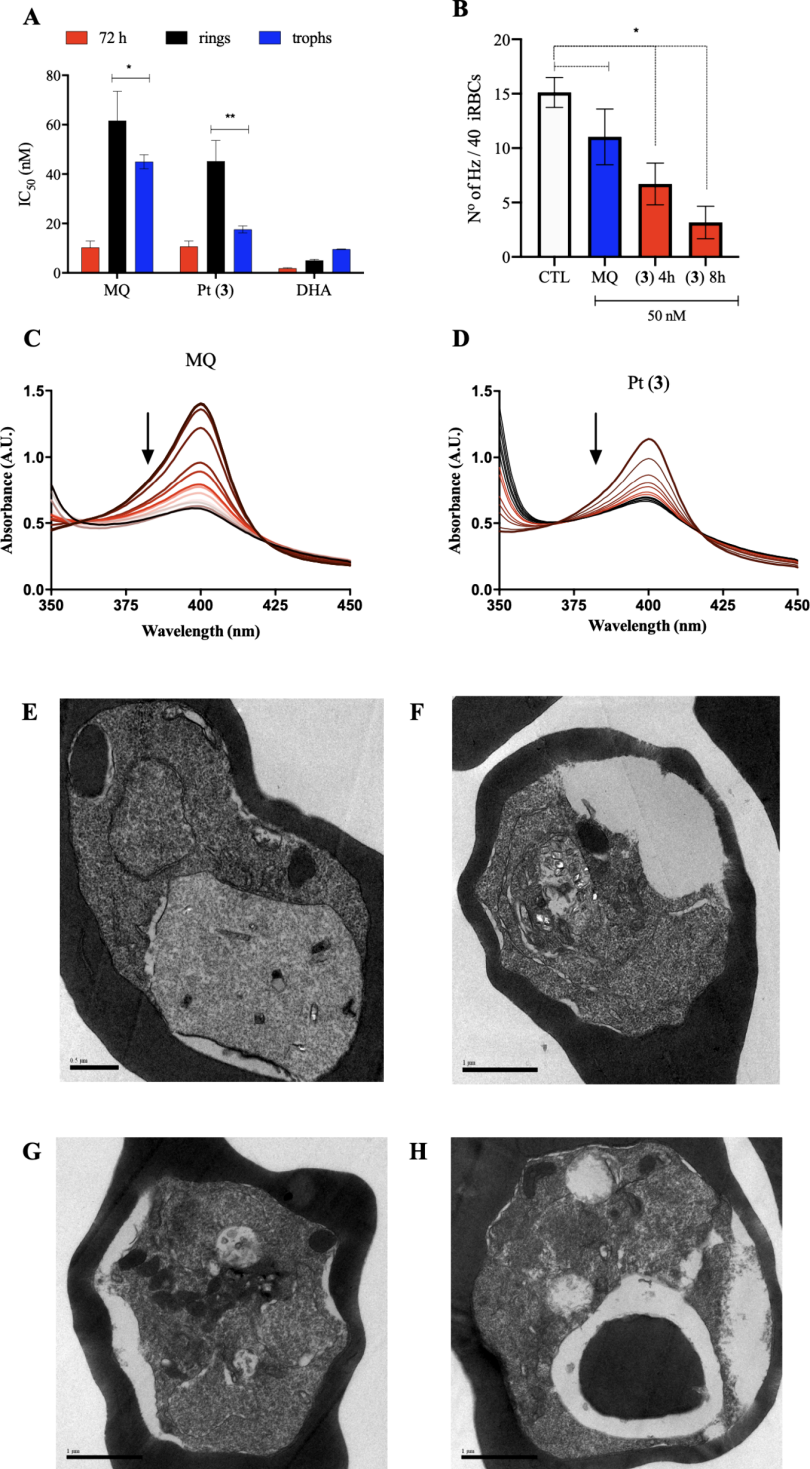
Pt (**3**) arrests trophozoite development by targeting heme detoxification of *P. falciparum*. A) Assessment of parasite stage susceptibility to treatment in 3D7 strain of *P. falciparum* after 72 h incubation by pLDH readout. Bars are the mean and error bars are SD of at least two independent experiments. *p < 0.05; **p < 0.01 by unpaired and nonparametric Mann–Whitney rank test. B) Quantification of the number of hemozoin crystals (Hz) per parasite cells in trophozoites of W2 strain of *P. falciparum*. Treatment at 50 nM was given at indicated times. Hz was visualized by transmission electron microscopy (TEM). Bars are the mean and error bars are SD. C, D) Representative titration spectra of Fe­(III)­PPIX (hemin) upon increasing compound concentration (indicated by arrow). Association constant (log *K*) values for compounds with hemin is shown in Table S4 in the Supporting Information. E–H) Representative micrographs of trophozoites of W2 strain of *P. falciparum* and visualized by transmission electron microscopy (TEM). Panel D is untreated control, 4 h. Panels E–G are treated with 50 nM of MQ, 4 h; Pt (**3**), 4 h and Pt (**3**), 8 h; respectively. Scale bar = 1 μm. Abbreviations: DHA = dihydroartemisinin; trophs = trophozoites; Hz = hemozoin.

Examination of trophozoite morphology by TEM (Figure 5D) showed that parasites retained an intact digestive vacuole albeit with a slightly reduced number of Hz crystals compared with untreated controls. A more common alteration was an atypical budding of organelles in the cytoplasm surrounding the digestive vacuole. At the same drug concentration, parasites treated with Pt (**3**) frequently displayed a markedly smaller digestive vacuole containing few Hz crystals, along with the cytoplasm alterations in organelles and the appearance of atypical structures resembling cytostomes. In MQ-treated parasites, the predominant morphological changes were localized to the cytoplasm rather than the digestive vacuole; consistent with its presumed mode of action.^38^ In contrast, Pt (**3**) induced pronounced alterations in both the cytoplasm and the digestive vacuole consistent with its superior ability to disrupt the heme detoxification pathway as well as additional Pt-associated mechanisms (Figure 5D).

Next, we assessed the ability of these complexes to induce oxidative stress and disrupt the glutathione redox balance. Here, trophozoites of the *P. falciparum* 3D7 strain were incubated with the compounds at concentrations ranging from 500 to 2000 nM, approximating MQ plasma levels. MQ did not induce oxidative stress under these conditions but the metal complexes and the control drug, DHA, triggered oxidative stress at 2000 nM (Figure 6A). To characterize this response, the potential of the compounds to alter intracellular GSH: GSSG ratio, where GSH represents the reduced dithiol and GSSG the oxidized disulfide, was investigated. For this analysis, a transgenic *P. falciparum* 3D7 line expressing a cytosolic biosensor (hGrx1-roGFP2), denoted here as *P. falciparum* 3D7^[hGrx1‑roGFP2]^, was used. Parasites were incubated with the compounds under the same conditions, and the fluorescence ratio of hGrx1-roGFP2 in living parasites was quantified by confocal microscopy.^39,40^ Diamide (DIA), as positive control, strongly perturbed the cytosolic GSH-dependent redox balance. In contrast, MQ and its metal complexes Pt (**3**) and Pd (**9**) did not alter the GSH: GSSG redox balance at 500 nM (Figure 6B). Extending the drug incubation time from 4 to 24 h reduced parasitemia, but still did not affect the GSH: GSSG redox ratio, confirming that these complexes do not directly disrupt the intracellular glutathione redox homeostasis of *P. falciparum*.

**Figure 6:**
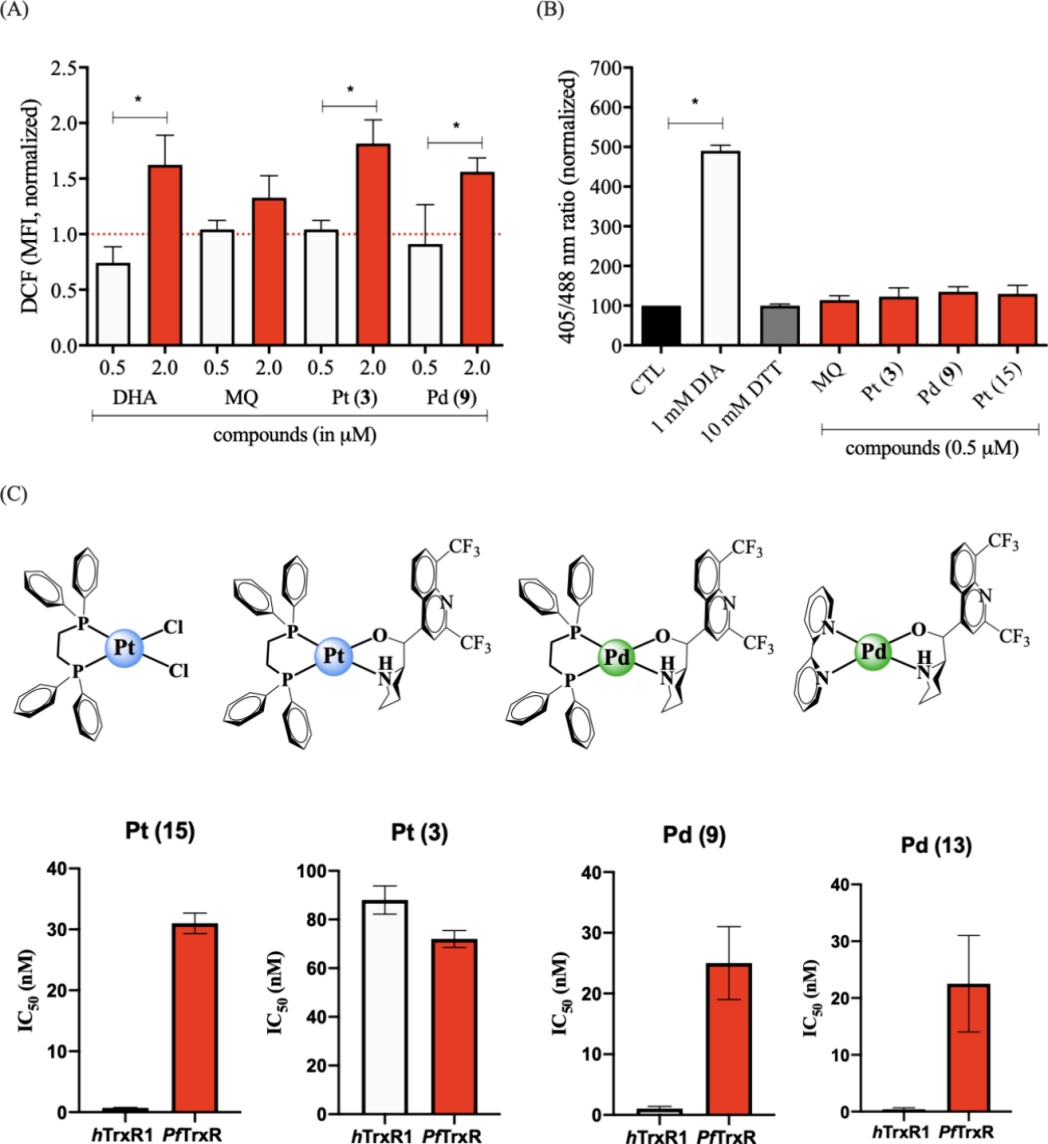
MQ–metal complexes arrest parasite development by increasing the general oxidative stress and inhibiting TrxR of *P. falciparum*. A) Quantification of reactive oxygen species in trophozoite stages in 3D7 strain of *P. falciparum* after 4 h incubation using the probe CM-H2-DCFDA and detected by flow cytometry. Indicated compound concentration are in μM. Bars are the mean and error bars are SD of two independent experiments. *p < 0.05, unpaired and nonparametric Mann–Whitney rank test. B) Quantification of parasite redox status under treatment using a genetically integrated ratiometric redox biosensor of *P. falciparum* 3D7^[hGrx1‑roGFP2]^ transgenic parasites. Trophozoites were treated for 4 h at concentration of 0.5 μM and fluorescence was quantified by confocal laser scanning microscopy. Bars are the mean ± S.E.M. of three independent experiments. ***p < 0.05 by one way-ANOVA and Dunnett post-test. C) Chemical structures of the metal complexes and their inhibitory effects on the enzymatic activity of recombinant flavoproteins *P. falciparum* thioredoxin reductase (*Pf*TrxR) and human thioredoxin reductase (*h*TrxR1). Raw values are shown in Table S7 in the Supporting Information. Abbreviations: DHA = dihydroartemisinin; CTL = untreated parasites, DIA = diamide, DTT = dithiothreitol.

Given the potential affinity of the metal centers for thiolates and selenolates, the ability of these complexes to inhibit thioredoxin reductases (TrxRs) activity was evaluated (Figure 6C; Table S7). The *P. falciparum* TrxR (*Pf*TrR) contains a cysteine rather than a selenocysteine at its redox-active catalytic site and its biochemical function overlaps with that of *P. falciparum* glutathione reductase (*Pf*GR).^41,42^ To address this limitation and gain broader insight in to the reactivity of the metal complexes toward thiolate and selenolate containing TrxR, we also assessed their inhibitory effects on the human thioredoxin reductase-1 (*h*TrxR1), a flavoenzyme that contains a selenocysteine in its catalytic redox center. The metal complexes inhibited TrxR enzyme activity (Figure 6C; Table S7). Analysis of the SAR and inhibition profile showed that the Pd complexes, Pd (**9**) and (**13**), were the most potent inhibitors of the selenocysteine containing *h*TrxR1 and were more effective against this enzyme than against the cysteine containing *Pf*TrxR. Overall, they were the strongest TrxR inhibitors among all complexes evaluated. The Pt (**3**) was less potent than its Pd (**9**) analogue, yet it inhibited all TrxRs with comparable potencies. Notably, this equipotent inhibition by Pt (**3**) appears to arise from the presence of the bidentate MQ ligand, as the related complex [Pt­(dppe)­Cl_2_] (**15**) exhibited a greater potency toward the selenocysteine enzyme *h*TrxR1 than toward the cysteine enzyme *Pf*TrxR.

The presence of labile chlorido ligands in the complex [Pt­(dppe)­Cl_2_] (**15**) likely accounts for its stronger inhibition of both thiolate and selenolate containing TrxRs. While such reactivity is expected for chloride Pt­(II) complexes, the extent of ligand scrambling from Pt (**3**) or Pd (**9**) in the presence of thiolates and selenolates was less predictable. Chemical stability of these complexes was monitored by NMR in the presence of GSH or GSSG (Figures S7 and S8). In the presence of GSH, Pt (**3**) underwent a 79% chemical transformation, yielding two products. One product corresponded with the replacement of bidentate MQ ligand by two GSH molecules coordinating in a monodentate mode. In the presence of GSSG, Pt (**3**) underwent a slower but progressive transformation, reaching 64% conversion and forming a single product attributed to partial dissociation of the bidentate MQ ligand with coordination of one GSSG molecule. In both conditions (GSH or GSSG), the transformation remained incomplete. In contrast, Pd (**9**) showed a distinct reactivity profile. In the presence of GSH, Pd (**9**) rapidly and completely transformed, consistent with pseudo-first order reactions.^43–45^ Two products were formed: one in which GSH and MQ each coordinated in a monodentate fashion, and another in which GSH bound in an *S*,*N*-bidentate mode. Similar coordination modes were observed in the presence of GSSG. Importantly, analysis of the NMR spectra indicated that MQ, rather than the phosphine ligand, was the ligand that dissociated from the complex.

#### Antischistosomal Activity

MQ has demonstrable activity against the skin-stage or newly transformed schistosomula (NTS) as well as adult worms of *S. mansoni*.^6^ Moreover, ferrocenyl and ruthenocenyl complexes of MQ have previously displayed antischistosomal activity.^8^ Thus, the antiparasitic activity of the Pt and Pd complexes were evaluated against NTS and adult *S. mansoni* worms (Figure 7A; Table S7). In this regard, Pt (**3**) showed superior activity against NTS compared with both MQ and the reference drug for schistosomiasis, Praziquantel (PZQ). Pt (**3**) also exhibited favorable selectivity, with a selectivity index of 12.7 against NTS of *S. mansoni* relative to J774 mammalian cells, compared with a selectivity index of 1.8 for MQ. Against adult *S. mansoni* worms, Pt (**3**) remained highly active, displaying at least half the potency of MQ and PZQ. In contrast, Pd complexes, Pd (**9**) and Pd (**13**) were less effective at impairing the viability of both NTS and adult worms compared to MQ. Our data confirm that MQ itself is an antischistosomal agent, whereas QN lacks such activity, and the amino-derivatives EMQ and TMQ were substantially less potent than MQ. The presence of MQ is essential for antischistosomal activity of Pt (**3**), as its metal precursor [Pt­(dppe)­Cl_2_] (**15**) displayed no activity (Table S7).

**Figure 7:**
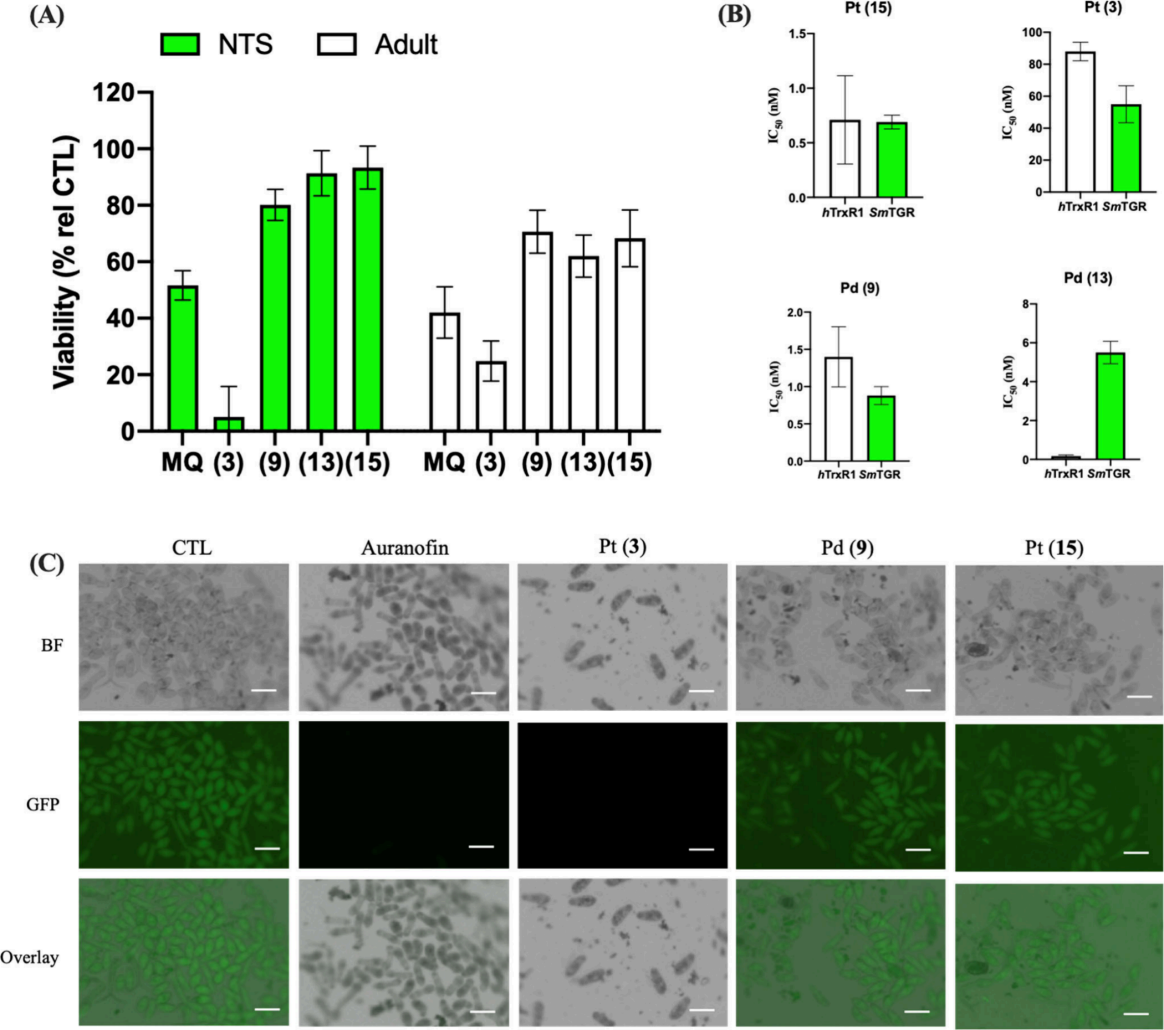
MQ–metal complexes inhibit worm viability by inhibiting the thioredoxin-glutathione reductase of *S. mansoni*. A) Effects of compounds on the viability of newly transformed schistosomula (NTS) where drugs were tested at 5 μM and adult worms at 15 μM of *S. mansoni* determined 24 h after drug exposure. B) IC_50_ values of the inhibitory effects on the enzymatic activity of *S. mansoni* thioredoxin-glutathione reductase (*Sm*TGR) versus *h*TrxR1. C) Representative micrographs of NTS of *S. mansoni* treated with complexes at 30 μM (or auranofin at 5.0 μM) for 2 h, stained with TRFS-Green and visualized by fluorescence microscopy. Scale bar = 200 μm. In panels A and B, values are mean and SD of triplicates. MQ = mefloquine. Raw values are shown in Table S7 in the Supporting Information.

Unlike *P. falciparum, S. mansoni* worms rely exclusively on thioredoxin-glutathione reductase (*Sm*TGR) to maintain their Trx/GSH redox homeostasis.^46–48^ Like *h*TrxR1, *Sm*TGR contains a selenocysteine in its redox-active catalytic site. Determination of IC_50_ values against the *Sm*TGR showed that the metal complexes inhibited its enzymatic activity in the low nM range, with inhibitory profiles closely resembling those observed for the human flavoenzyme *h*TrxR1 (Figure 7B; Table S7).

In this regard, Pt (**3**) was evaluated for inhibition of *Sm*TGR activity *in situ* in NTS (Figure 7C). To do this, NTS were exposed to the compounds for 2 h, stained with the fluorescent probe TRFS-green, a selective fluorescent substrate for TGR in schistosome worms,^46^ and imaged by fluorescence microscopy. Untreated worms displayed strong TRFS-green fluorescence, while worms treated with auranofin, the reference TGR inhibitor, exhibited a pronounced reduction in fluorescence intensity, confirming inhibition of *Sm*TGR in living worms. Treatment with Pt (**3**) similarly produced a strong decrease in fluorescence, whereas the metal precursor [Pt­(dppe)­Cl_2_] (**15**) or the Pd analogue (**9**) did not decrease the TRFS-green fluorescence in the worms. Visual inspection of worm fluorescence was consistent with microplate base quantification. Furthermore, decreasing the concentrations of Pt (**3**) and auranofin also led to graded reduction in fluorescence intensity, suggesting a concentration-dependent effect. Measurement of the GSH: GSSG ratio in NTS was not performed, as the combined evidence, potent inhibition of recombinant *Sm*TGR, strong antischistosomal activity against both NTS and adult worms and clear suppression of intracellular *Sm*TGR activity in a phenotypic assay, strongly supports that Pt (**3**) inhibits *Sm*TGR within a cellular context.

#### *In Vivo* Efficacy and Metal Distribution

We evaluated the *in vivo* efficacy of frontrunner complexes Pt (**3**) and Pd (**9**) in Swiss mice infected with *P. berghei* blood schizonts.^49^ Efficacy was first assessed using the Peters’ suppressive test, in which treatment is administered by daily intraperitoneal injection beginning 3 h postinfection and continued for four consecutive days. Pt (**3**) exhibited a dose-dependent suppressive effect on parasitemia and demonstrated superior efficacy compared to MQ. At a dosage of 22 μmol/kg/day, Pt (**3**) achieved complete suppression of parasitemia and enabled mouse survival, whereas MQ required a higher dosage of 33 μmol/kg/day to achieve comparable efficacy. In contrast, Pd (**9**) showed suppressive effect similar to that of MQ (Figure 8A and 8B; Table 3, Tables S8 and S9). The curative potential of these complexes was then assessed using the Thompson test, in which treatment is initiated on day 3 postinfection. As expected, combination therapy with artesunate plus MQ produced 100% cure rate in the positive control group. Under the same regimen, Pt (**3**) achieved a 40% cure rate, outperforming MQ alone, which cured 20% of infected mice (Figure 8C and 8D).

**Figure 8:**
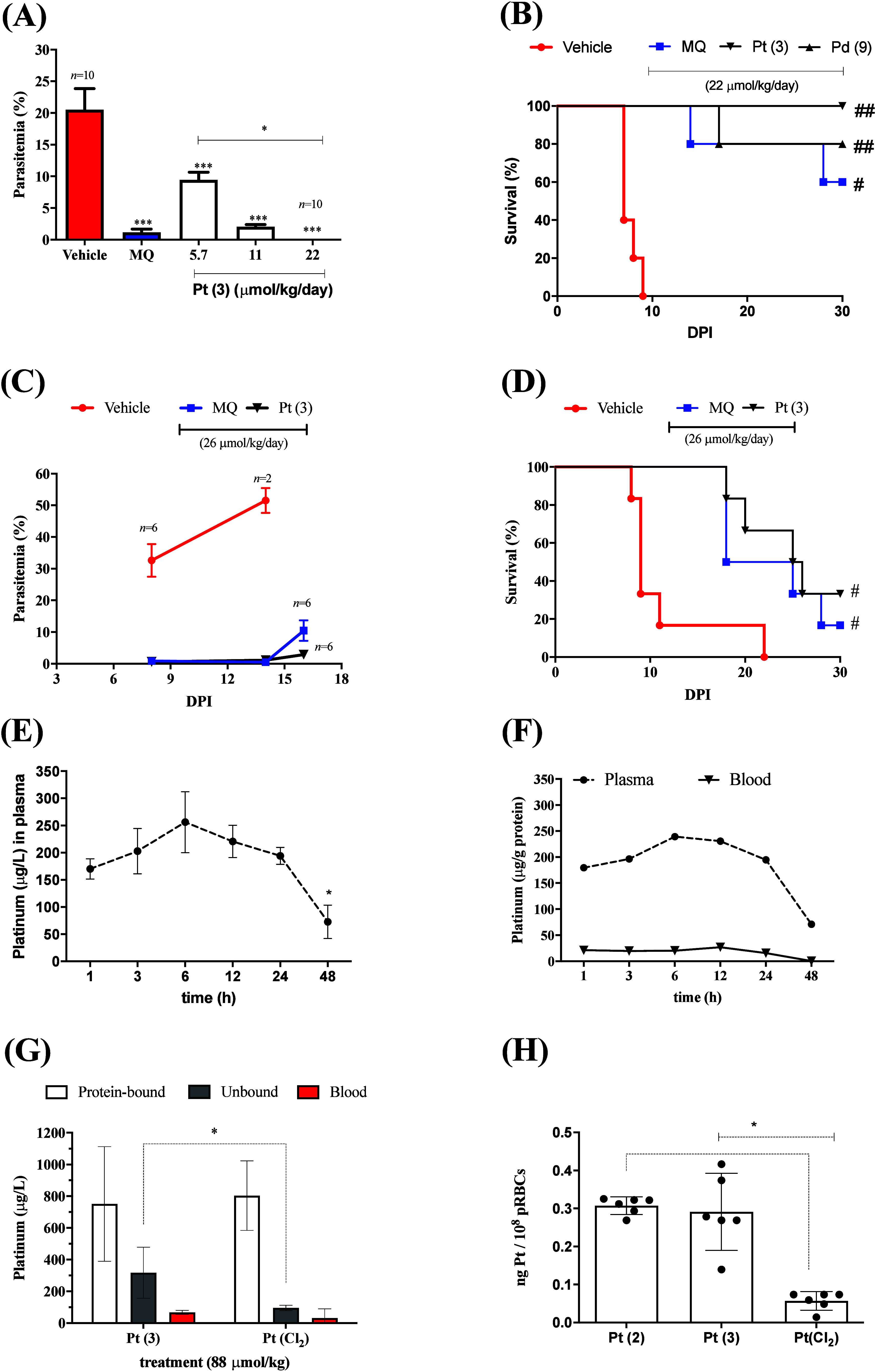
Profiling the pharmacodynamic and pharmacokinetic of MQ–metal complexes. A, B) Suppressive Peters test (treatment initiated 24 h postinfection) on parasitemia and animal survival. MQ was given at a dose of 22 μmol/kg. C, D) Curative Thompson test (treatment initiated 3 days postinfection) on parasitemia and animal survival. Efficacy was performed in NK65 strain of *P. berghei*-infected Swiss mice, parasitemia was determined by flow cytometry. E, F) Plasma and blood concentration of platinum in single-dosed Pt (**3**) at 88 μmol/kg in uninfected mice. G) Distribution of platinum concentration in single-dosed Pt (**3**) and matched Pt (Cl_2_) at 88 μmol/kg in uninfected mice determined after 6 h of intravenous injection treatment. H) Parasite uptake of platinum content at drug concentration of 1000 nM in *ex vivo* pRBCs determined after 6 h of treatment. Platinum concentrations in plasma, blood and pRBCs were determined by ICP-MS. DPI = days postinfection. For A–F: treatment was given by intraperitoneal route, an *n* = 5/group was employed (unless indicated) and values are mean and S.E.M.; for panel F, values are medians. Error bars indicate S.E.M. Supporting data is shown in Tables S8 and S9. *p < 0.05, ***p < 0.01 (one-way ANOVA and Dunnett post-test); ^#^ *p* < 0.05, ^##^ *p* < 0.01 (log-rank, Mantel-Cox test). Abbreviations: DPI = days postinfection; MQ = mefloquine; Pt­(Cl_2_) = [Pt­(dppe)­Cl_2_] (**15**); pRBCs = parasitized *P. berghei*-red blood cells.

**Table 3:** Summary of the Parameters Calculated from *P. berghei*-Infected Mice and the *in Vitro* Metabolism Determined in the Mouse Microsome

	Suppressive efficacy in Peters’ 4-day testing in *P. berghei*-infected mice^a^			
Compounds	ED_50_ (μmol/kg)	ED_99_ (μmol/kg)	Curative efficacy (%) in Thompson test^b^	Intrinsic clearance (Cl_int_) (μL/min/mg)^c^
MQ	7.3	33	20	14.48
Pt (**3**)	5.7	22	40	264.18
Pd (**9**)	12	24	N.D.	25.33
MQ+ART	-	-	100	-

a Parasitemia was determined at day 7 postinfection and employed to calculate % parasitemia reduction and the ED_50_ and ED_99_ values.

b Percent of cure determined by monitoring animal survival until 30 days postinfection.

c Cl_int_ = intrinsic clearance. Values are the average of at least *n* = 2 replicates. Abreviations: MQ = mefloquine; ART = Artesunate.

The mean blood and plasma concentration–time profiles of Pt were determined following a single intraperitoneal dose of Pt (**3**) at 88 μmol/kg in uninfected C57BL/6 mice using inductively coupled plasma mass spectrometry (ICP-MS). This dose corresponds to the total amount administered over the Peters’ test regimen and was well tolerated, with no observed adverse effects. The resulting concentration–time curves are shown in Figure 8E. Plasma concentrations peaked at 6 h (256 μg/L) and then declined to 75 μg/L by 48 h. After adjusting Pt concentration using hematocrit and plasma protein content, we observed Pt levels in whole blood did not parallel those of plasma, with a blood-to-plasma concentration ratio <0.1 (Figure 8F).

In a parallel experiment, we quantified Pt levels 6 h after a single intravenous injection of Pt (**3**) at the same dose (88 μmol/kg) (Figure 8G). Pt concentrations in blood and in fractionating the plasma (protein-bound and fractionated plasma), were measured. The mean Pt concentrations intravenous dosing was 1068 ug/L, approximately 4-fold higher than after intraperitoneal injection, corresponding an estimated intraperitoneal bioavailability of 41%. Regardless of the route of administration, Pt levels were consistently higher in plasma than in blood. Further fractionation revealed approximately twice as much Pt in protein-bound versus unbound plasma fractions (Figure 8G). Notably, unbound Pt concentrations following Pt (**3**) administration were substantially higher than those observed for the Pt precursor (**15**), likely due to the lability of chlorido ligands in (**15**), which promotes protein binding via ligand exchange. Pt (**3**) displayed a higher apparent intrinsic clearance than Pd (**9**) in mouse liver microsomes, whereas MQ displayed low clearance (Table 3).^50^

Given the relatively low blood-to-plasma concentration ratio of Pt following Pt (**3**) administration, corresponding to an estimated blood concentration of 20 nM compared to a mean IC_50_ of 2.1 ± 0.2 to 7.3 ± 1.5 nM against ABS of *P. falciparum*, we investigated whether Pt was reaching parasitized red blood cells (pRBCs) in *P. berghei* infected mice. *In vitro* uptake studies were performed by incubating pRBCs harvested from mouse blood for 6 h under treatment with 100 nM of Pt (**2**), Pt (**3**), and Pt (**15**) followed by Pt quantification via ICP-MS. Treatment with Pt (**2**) and Pt (**3**) led to significantly higher intracellular Pt levels compared with precursor [Pt­(dppe)­Cl_2_] (**15**) (Figure 8H), suggesting that MQ plays an essential role in promoting efficient accumulation of platinum complexes in pRBCs.

To directly confirm intracellular Pt localization, pRBCs isolated from *P. berghei*-infected mice were treated with Pt (**3**), imaged by TEM and analyzed in parallel by energy-dispersive X-ray spectroscopy (EDXS). While ICP-MS provided bulk Pt quantification, EDXS offered spatially resolved elemental detection, confirming Pt intracellular accumulation within parasite cells. Microscopic analysis revealed that both Pt (**3**) and MQ induced morphological alterations and reduced Hz crystal formation in *P. berghei* pRBCs under both *in vitro* and *in vivo* conditions (Figure 9A–C). EDXS showed that, after 4 h of treatment with Pt (**3**), intracellular Pt levels were significantly higher in parasites exposed to Pt (**3**) than to precursor [Pt­(dppe)­Cl_2_] (**15**) (Figure 9D). Together, the ICP-MS and EDXS data demonstrate that Pt (**3**) effectively delivers Pt into *Plasmodium* cells.

**Figure 9:**
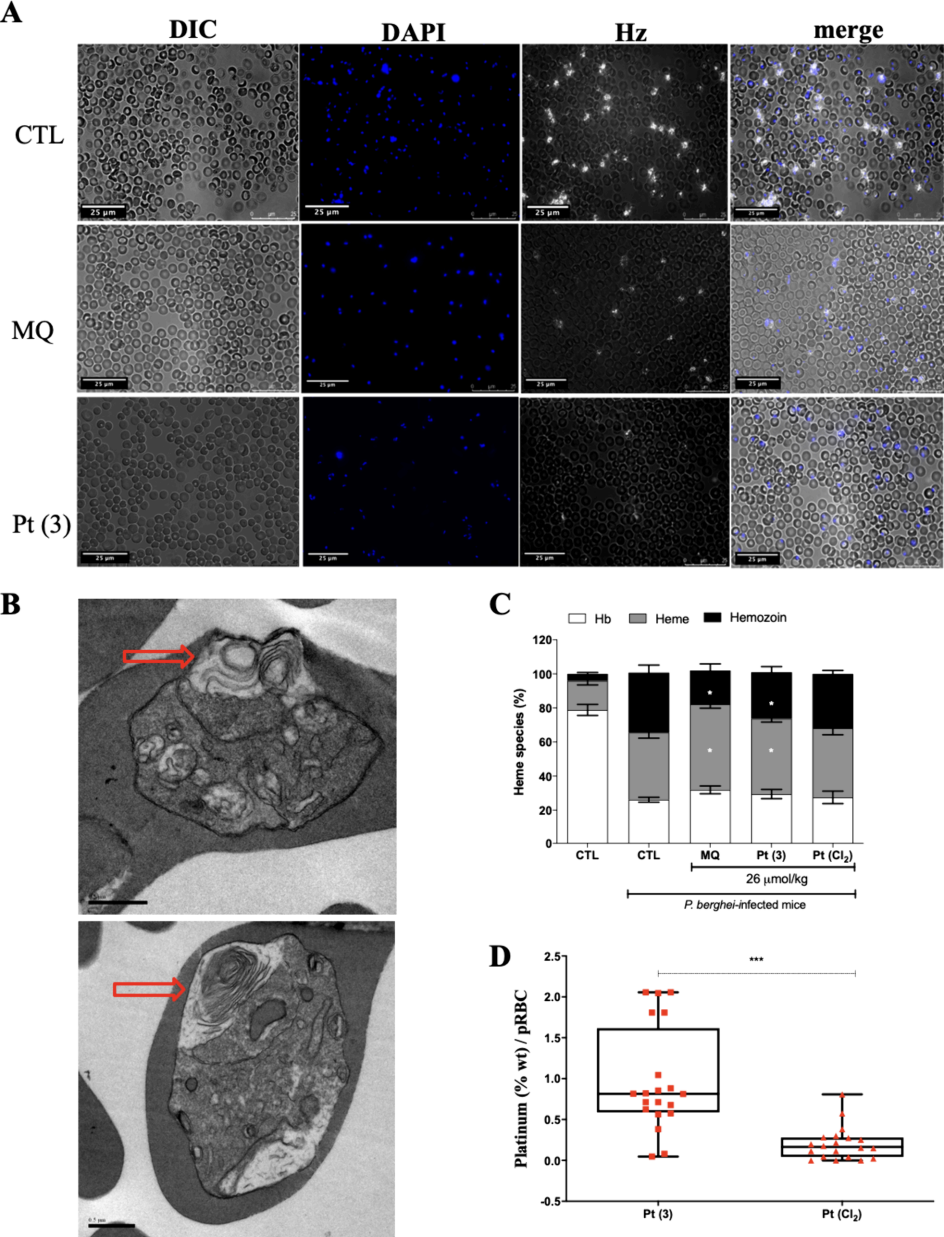
MQ–metal complex Pt (**3**) suppresses the *Plasmodium* heme detoxification, leads to an accumulation on Pt content in pRBC and concomitantly causes morphological alterations in trophozoites of *P. berghei*-infected mice. A) *P. berghei*-infected mice at 10% parasitemia received intraperitoneal treatment of a single-dosed drug at 26 μmol/kg. After 24 h of treatment, blood smears were mounted and hemozoin (Hz) was visualized by reflection contrast polarized light microscopy, while nuclei were stained with DAPI. B) Ultrastructural alterations in parasite morphology were visualized by transmission electron microscopy (TEM) after 4 h of treatment, which received intraperitoneal treatment of a single-dosed drug at 26 μmol/kg of Pt (**3**). Scale bar = 0.5 μm. C) Quantification of heme species in the blood of *P. berghei*-infected mice shows a decrease in Hz content and an increase in heme levels after 24 h of treatment with MQ and Pt (**3**) compounds. D) Quantification of Pt content in pRBCs isolated in the blood of *P. berghei*-infected mice and determined by energy-dispersive X-ray spectroscopy (EDXS) after 4 h of *ex vivo* drug treatment. Panels C and D) Bars are mean and error bars are SD of one single experiment. Mouse samples were the same in panels A and C. *p < 0.05, ***p < 0.01 (one-way ANOVA and Dunnett post-test). Abbreviations: Hz = hemozoin. MQ = mefloquine; Pt­(Cl_2_) = [Pt­(dppe)­Cl_2_] (**15**); pRBCs = parasitized red blood cells; Hb = hemoglobin; DIC = differential interference contrast.

## Discussion

We have attempted to improve our current understanding of the structural determinants required for designing metal-based drugs for parasitic infectious diseases. First, we established an efficient route, in terms of simplicity, purification procedures and yields, for the synthesis of MQ–metal complexes (**1**–**13**). We found that the reactivity of MQ is unique, behaving as a bidentate ligand toward forming the metal complexes with Pt and Pd. This is contrast to the aminomethanol QN that is not capable of behaving as a bidentate ligand. This is an unprecedented finding, and differs substantially from other antimalarial drugs containing a quinolinic heterocycle, which are all capable of forming metal complexes as a monodentate ligand.^18–20^ Comparatively, the MQ–Pt complex (**1**) containing a bidentate antimalarial ligand was more soluble and stable in aqueous medium, as well as less prone to undergo ligand exchange reactions in the presence of the reductant GSH than its parental Pt counterpart (**14**), which contains a QN monodentate ligand.

One concern with positively charged complexes containing a bidentate ligand, as the complexes (**1**–**13**), is the potential of ligand coordination to metal to hinder the (2-)­piperidinyl methanol access for achieving antiparasitic activity. Our SAR data set with the amino-derivatives of MQ (EMQ and TMQ), as well as the MQ derivatives AMQ and CMQ all argued that the (2-)­piperidinyl methanol group is essential for MQ interacting with the molecular targets and to achieve antiparasitic activity. However, our SAR data set argued that these metallic complexes (**1**–**13**) exhibit a potent and efficacious antiparasitic property against the ABS of *P. falciparum* parasites and against both skin-stage and adult *S. mansoni* worms.

It is crucial to discuss the physical-chemical properties which are determinants for the SAR of complexes (**1**–**13**). The interplay of aqueous solubility versus lipophilicity, and the kinetics of ligand exchange reactions is undoubtedly complex, but these are likely key determinants. How these factors can impact the antiparasitic activity of metal-based drugs was largely unknown. We hypothesized that the nature of phosphine ligands likely contributes to the reactivity profile of complexes (**1**–**13**) in solution. For Pt complexes **3** and **4**, we attributed that the employed phosphines, forming a five- and six-membered chelated rings with Pt respectively, contribute to an overall drug stability, resulting in low reactivity for ligand exchange reactions.

The interplay between lipophilicity versus antiparasitic activity should also be important. Although we did not directly examine the lipophilicity for these complexes, it is well-known that phosphine ligands can very often increase the lipophilicity in comparison to complexes devoid of phosphine.^51–53^ In support of this, Pt complexes were found to accumulate in and deliver Pt to the parasite cells, indicating that these possess a desirable degree of lipophilicity. With all these considerations in mind, we propose that the antiparasitic profile of complexes (**1**–**13**) is due to the relatively moderate aqueous solubility of MQ, in addition to a gain in lipophilicity conferred by phosphines. More importantly, an increase in lipophilicity induced by phosphine coligands does not jeopardizes the inert profile of the complexes in the context of ligand exchange reactions. This is a result of the chelated ring between metal and lipophilic phosphines, which in our interpretation, can be very stable and to be less prone to undergo ligand exchange reactions.

Another concern with complexes of Pt­(II) species (**1**–**13**) is the potential lack in preferentially targeting pathogens. It is well-known for Pt­(II) agents, albeit less known for Pd­(II) agents, that they can exhibit a reactivity toward thiolate and selenolate species by undergoing ligand exchange reactions in the blood and this could conceivably confer a limited efficacy for addressing tissues other than blood.^54,55^ However, the selectivity index, the *in vivo* efficacy in mice, and the determined Pt concentrations all indicate that these complexes can selectively target pathogens in the blood rather than mammalian cells by preferentially delivering metallic species to the pathogens. We noted that the presence of bidentate ligand MQ is crucial for complexes selectively recognizing the targeted pathogens.

Conceivably, the metal-bidentate ligand conjugate should reach its molecular targets. Regarding to this point, we noted that the complexes of Pt were more efficient to recognize and deliver the metallic species to the pathogens than the complexes of Pd. Although the complexes of Pt were more water-soluble than the Pd counterpart, we argued that the reactivity of complexes of Pd in the presence of nucleophilic species is higher than the respective complexes of Pt and this is a determining factor. While this is obvious for less-functionalized complexes or salt containing Pd,^56^ this was less obvious for complexes of Pd featuring quite-stable bidentate ligands, such as in the MQ–Pd complexes (**8**–**13**).

Biologically available thiolate/selenolate species can play a dual role for metal complexes: by acting as a drug activator, through ligand exchange reactions, and also playing a role as a molecular target. Conceivable, GSH is a key drug activator and responsible for metal speciating and ligand dissociation. We found that the reactivity of MQ–Pd complexes (**8**–**13**) in the presence of thiolate species were remarkable dissimilar than MQ–Pt complexes (**1**–**7**). While the MQ was dissociated from metal species from both complexes of Pt and Pd, the kinetics of MQ dissociation from the complexes of Pt was significantly more gradual and slowly than the observed for the complexes of Pd. Our data further support that the complex Pt (**3**) is of enough stability to reach cellular compartments in the pathogens to engage on its molecular targets, such as TrxRs and heme species.

Given our extensive profile of the inhibitory activity of complexes (**1**–**13**) for TrxRs, we concluded that the complexes of Pd are more potent in inhibiting TrxRs than Pt complexes. This is not entirely a surprise, as this is consistent with previous observations that Pd­(II) salts are more potent inhibitors of human TrxR1 than Pt­(II) salts.^56^ Besides potency, it is pivotal to discuss the selectivity versus promiscuous profiles. Pd complexes were selective in targeting the selenolate TrxRs of *S. mansoni* and the human more effectively than the thiolate *Pf*TrxR; conceivably this is due to a kinetic aspect.^57^ Pt complexes were less potent than Pd complexes but inhibited all three TrxRs. The presence of MQ was essential to maintain the activity of Pt (**3**) in equally inhibiting all three TrxRs, while the complex [Pt­(dppe)­Cl_2_] (**15**) inhibited with higher potency the selenolate TrxRs than their counterpart thiolate one.

Outcomes of drug targeting parasite TrxRs can differ substantially among pathogenic parasites. *S. mansoni* worms would be more susceptible to drugs inhibiting the enzymatic activity of *Sm*TGR, while conversely, *P. falciparum* is presumably less susceptible to drugs inhibiting its *Pf*TrxR as the *Pf*GR has a redundancy in function. Indeed, this is the case of pan-TrxR inhibitor Auranofin acting as a potent antiparasitic agent for *S. mansoni* but not for *P. falciparum.* ^58,59^ With this caveat noted, complex Pt (**3**) has induced an intracellular oxidative stress, but it was not capable in causing an imbalance in the homeostasis of GSH: GSSG ratio in *P. falciparum*, a phenotypic outcome expected for any drug efficiently targeting cellular *Pf*TrxR and *Pf*GR function. Therefore, in drug-susceptible *P. falciparum* strains, complex Pt (**3**) did not affect the redox homeostasis of GSH as its main mechanism of action but rather than a secondary mechanism. However, against *S. mansoni*, the observed cell-based activity and TrxR inhibition are all consistent with complexes behaving as inhibitors of *Sm*TGR activity in *S. mansoni* worms as the main mechanism of action.

Furthermore, it is important to mention that little is known about whether MQ-resistant *P. falciparum* parasites have an altered expression of *Pf*TrxR compared to parental, drug-susceptible parasites. For comparison, Artemisinin partially resistant parasites can exhibit an increased expression on *Pf*TrxR compared to parental parasites.^60^ As drug resistances are redox-mediated phenomena and with this caveat noted, we show that complex Pt (**3**) has improved activity against parasites of MQ-resistant TM91C235 strain, which is not reproduced after treatment by MQ. This was achieved at a drug concentration that overcome the efflux of drug transport. It is possible that a stronger oxidative stress and a reduction of the catalytic activity of *Pf*TrxR is achieved by Pt (**3**) to inhibit MQ-resistant parasite growth. These findings suggest a vulnerability of the parasites harboring multiple copies of the *pfmdr1* gene that is perfectly amenable to antiplasmodial drug discovery targeting TrxR.

With the examination of Pt distribution in plasma, blood, and *P. berghei* parasites after complex Pt (**3**) dosing, and further supported by parallel assessment of complex [Pt­(dppe)­Cl_2_] (**15**), it was possible to conclude that complex Pt (**3**) exhibits an extended exposure pharmacokinetic profile, similar to MQ, with plasma concentrations maintained 48 h after dosing. This quantitative examination was further confirmed in a qualitative study by EDXS, where parasites undergoing morphological alterations concomitantly exhibited an accumulation of Pt. This observed Pt distribution resulting from complex Pt (**3**) administration is most likely attributable to the fact that complex Pt (**3**) has a good aqueous stability, adequate lipophilicity, and it is less prone to undergo ligand exchange reactions in comparison to other complexes.

## Conclusions

Circumventing *P. falciparum* drug resistance and developing new drugs for schistosomiasis remain key priorities for global disease control and elimination efforts. Mefloquine (MQ) and quinine (QN) possess multiple coordination sites, making them attractive scaffolds for incorporation into metal complexes. This work focused on a new series of MQ–metal (Pt and Pd) complexes with chemically diverse coligands as promising antiplasmodial and antischistosomal agents. The complexes inhibited the growth of both drug-sensitive and drug-resistant *P. falciparum* strains at single-digit nanomolar concentrations. They also exhibited activity against both newly transformed schistosomula and adult *S. mansoni* worms, in some cases surpassing MQ and Praziquantel (PZQ). Notably, bidentate coordination of MQ was essential for achieving potent antiparasitic effect, and importantly, the stability of the complexes. Phosphine coligands strongly modulated the balance between aqueous solubility and lipophilicity in Pt complexes, whereas this effect was less pronounced in Pd analogues.

MQ–Pd complexes (**8**–**13**) displayed reactivity toward the thiolate species (GSH and GSSG) distinct from that of MQ–Pt complexes (**1**–**7**). Although MQ dissociation occurred in both Pt and Pd complexes, the kinetics were markedly slower and more gradual for Pt complexes. Pt (**3**) possessed sufficient stability to reach intracellular compartments within the parasite and engage key molecular targets, such as TrxRs and heme species, which were confirmed as potential mechanisms through which the complexes displayed their antiparasitic activity.

Metal-based drugs often fail in *in vivo* efficacy studies due to rapid reactivity with nucleophiles in the bloodstream. With this concern in mind and guided by initial evaluation of aqueous stability even in the presence of GSH, we have further demonstrated that Pt (**3**) complex effectively suppresses and cures *P. berghei*-infection in mice. Its activity *in vivo*, following intraperitoneal dosing, was approximately twice that of MQ. Analysis of Pt distribution in plasma, blood, and parasitized erythrocytes, together with comparison to the precursor [Pt­(dppe)­Cl_2_] (**15**) revealed that Pt (**3**) exhibited an extended pharmacokinetic exposure profile similar to MQ, with plasma concentrations maintained for up to 48 h after dosing.

The results generated in this work support further studies, for example, expanding the scope of the metal center and drug partner, to identify new compounds with improved *in vitro* and *in vivo* antiplasmodial and/or antischistosomal activity. Further, drug leads with complementary TrxRs and hemozoin-formation inhibitory activities would support ongoing efforts to address *P. falciparum* drug resistance and to discover and develop new drugs for schistosomiasis.

## Experimental Section

### Chemistry

#### Materials for Synthesis

All chemical syntheses were carried out under argon with standard Schlenk techniques. Solvents were purified by standard procedures immediately prior to use. K_2_[PtCl_4_] and *trans*-[PdCl_2_(PPh_3_)_2_] was purchased from Precious Metals (Australia). Triphenylphosphine (PPh_3_), 1,1-bis­(diphenylphosphine)­methane (dppm), 1,3-bis­(diphenylphosphine)­ethane (dppe), 1,3-bis­(diphenylphosphine)­propane (dppp), 1,4-bis­(diphenylphosphine)­butane (dppb), 1,1′-bis­(diphenylphosphine)­ferrocene (dppf), 1,3,5-triaza-7-phosphaadamantanequinoline (PTA), 2,2′-bipyridine (bipy), quinine, AgPF_6_, NH_4_PF_6_, and hemin chloride were sourced from Sigma-Aldrich (St. Louis, MO, USA). Mefloquine hydrochloride was obtained from FarManguinhos (Brazil). Reagent grade solvents were appropriately distilled and dried before use. All other commercial reagents were used without further purification. Mefloquine in its free base form was obtained by solubilizing the hydrochloride salt in an aqueous solution containing equimolar concentration of K_2_CO_3_, stirring, cooling, filtering the mefloquine free base, and recrystallization in ethyl ether. Mefloquine derivatives *erythro*-11-aminomefloquine (EMQ) and *threo*-11-aminomefloquine (TMQ) were synthesized as previously described.^30^ *Rac*-amidemefloquine (AMQ) and carboxymefloquine (CMQ) were kindly supplied by WorldWide Antimalarial Resistance Network (WWARN) Reference Standards Program (Thailand).

#### Instrumentation

The infrared spectra were recorded on an FTIR Bomen-Michelson 102 spectrometer in the 4000–200 cm^–1^ region. Ultraviolet–visible (UV–vis) spectra were recorded on an HP 8452A diode array spectrophotometer. All NMR experiments were performed at 298 K on a Bruker DRX 400 MHz spectrometer, at 9.4 T, observing ^1^H at 400.13, ^13^C­{^1^H} at 100.62, ^31^P­{^1^H} at 161.98 MHz. The NMR spectra were recorded in acetone-*d*_6_ and DMSO-*d*_6_, with TMS (^1^H and ^13^C­{^1^H}), 85% H_3_PO_4_ (^31^P­{^1^H}) as the internal and external references, respectively. ESI­(+) mass spectra were obtained by direct infusion in a Waters Synapt Mass Spectrometer in positive ion mode, utilizing acetone:water and acetonitrile:water (LC/MS grade from Honeywell; B&J Brand) as the solvent. Purity of all metal complexes was confirmed by elemental analysis and found to be in accordance with ACS standards (purity was >95%).

#### General Methods of Chemical Synthesis

##### Synthesis of Complexes [Pt­(P)_2_Cl_2_] (**P1**–**P7**)

The platinum precursors were synthesized as described in the literature, or with little modification.^51,61^ A solution of bisphosphine (1.85 or 3.70 mmol in the case of PPh_3_) was dissolved in 50 mL of methanol (for **P1**–**P4**) and ethanol (for **P5**, **P6**), heated in reflux under an inert atmosphere until complete solubilization. Afterward, heating was turned off and then 750 mg of K_2_[PtCl_4_] (1.81 mmol) in 120 mL of dichloromethane (for **P1**–**P3**) and chloroform (for **P4**–**P6**) was added. The mixture was stirred for 30 min, then 20 mL of distilled water (**P1**, **P2**, **P4**–**P6**) was added and the mixture was refluxed for 24 h. The crude mixture was concentrated, and the resulting precipitate was filtered, triturated several times with hexanes and diethyl ether and finally dried under a vacuum. For **P3** (also identified as **15**) complex, the resulting crude solid was solubilized in 20 mL of DMF and recrystallized in diethyl ether. For the complex **P7**, a solution of 200 mg of PTA (1.47 mmol) and 20 mL of ethanol was refluxed under an inert atmosphere until complete solubilization, then a solution of K_2_[PtCl_4_] (0.67 mmol) in 4 mL of distilled water was added. The mixture was kept at reflux for 2 h, then placed in an ice bath, the resulting solid was filtered and washed with ether and finally dried under a vacuum.

##### Synthesis of Complexes [Pd­(P)_2_Cl_2_] (**P8**–**P12**) and [Pd­(2,2′-bipyridine)­Cl_2_] (**P13**)

A solution of *trans*-[Pd­(PPh_3_)_2_Cl_2_] (1.85 mmol) was dissolved in 50 mL of dichloromethane, to which a 2.0 mmol of bisphosphine or 1.2 mmol of 2,2′-bipyridine and 40 mL of dichloromethane were added, and the mixture was refluxed for 24 h under an inert atmosphere. Then, the mixture was concentrated, and hexane was added to precipitate the complex; the resulting solid was washed with hexanes and ether and finally dried under a vacuum.

##### Synthesis of Complexes [M­(MQ)­(P)_2_]­PF_6_ and [Pd­(MQ)­(bipy)]­PF_6_

A methanolic solution containing 0.18 mmol of mefloquine salt and 0.36 mmol of NaHCO_3_ was added into a Schlenk flask containing a solution of [M­(P)_2_Cl_2_] or [Pd­(bipy)­Cl_2_] (0.15 mmol) dissolved in 50 mL of dichloromethane (for complexes **1**–**8** and **13**) or 50 mL of methanol (for complexes **9**–**12**), For the synthesis of **1**, **2**, **4**–**6**, **8**–**10** and **12**, a 0.20 mmol of NH_4_PF_6_ was added and the mixture was stirred for 24 h. For complexes **3**, **7**, **11** and **13**, it was added 0.30 mmol of AgPF_6_ and the mixture was stirred for 4 h, for which the solution was filtrated for removal of AgCl. For all the complexes, the volume of the solvent was reduced under a vacuum. The resulting solid was washed with water and dried under a vacuum.

##### *cis*-[Pt­(PPh_3_)_2_(MQ)]­PF_6_ (**1**)

A white solid was obtained with a yield of 89.3% (168 mg). Elemental analysis (%) Calc. for C_53_H_45_F_12_N_2_OP_3_Pt: C 51.26; H 3.65; N 2.26. Found: C 51.16; H 3.39; N 2.33. IR [*v*_max_ cm^–1^ (assignation)]: ν 3244 (N—H), ν 3061 (C—H aromatic), ν 2945 (C—H aliphatic), ν 1620 (C=C), ν 1587 and 1568 (C=N), ν 1105 (P—C), 843 (PF_6_), ν 557 and 552 (Pt—P). UV–vis (DMSO) [Log ε, λ (assignation)]: 4.34 M^–1^ cm^–1^, 264 nm (π–π* Ph); 4.31 M^–1^ cm^–1^, 270 nm (M → L); 4.25 M^–1^ cm^–1^, 278 nm (L → M); 3.86 and 3.71 M^–1^ cm^–1^, 304 and 316 nm (π–π* MQ). NMR-^1^H (acetone-*d*_6_) [δ ppm, (integral; multiplicity; assignation, *J* Hz)]: 0.71; 2.12 (2H, d, H6′, ^3^ *J* = 13.20 Hz), 1.17; 1.75 (2H, m, H5′), 1.30; 1.81 (2H, d, H4′, ^3^ *J* = 12.00 Hz), 1.72; 2.63 (2H, m, H3′), 3.53 (1H, d, H1′, ^3^ *J* = 8.00 Hz), 6.16 (1H, bs, NH), 6.22 (1H, bs, H1″), 7.04 (1H, s, H3), 7.33; 7.44 (12H, m, Hc), 7.52 (6H, m, Hd), 7.56; 7.86 (12H, m, Hb), 7.86 (1H, m, H6), 8.25 (1H, d, H5, ^3^ *J*_*ortho*_ = 7.2 Hz), 8.51 (1H, d, H7, ^3^ *J*_*ortho*_ = 8.00 Hz). NMR-^13^C­{^1^H} (acetone-*d*_6_) [δ ppm, (multiplicity; assignation, *J* Hz)]: 23.1 (C6′), 23.5 (C5′), 26.0 (C4′), 49.2 (C3′), 68.4 (C1′), 80.1 (C1″), 117.1 (C3), 123.5 (C9), 126.2 (C10), 127.8 (C4), 128.3 (C6), 128.3; 129.8 (Ca), 129.2; 130.1 (Cc), 129.3 (C12), 129.5 (C7), 129.9 (C5), 132.5; 132.9 (Cd), 135.1; 135.6 (Cb), 143.5 (C8), 148.4 (q, C11, ^1^ *J* C—F = 35.22 Hz), 152.9 (d, C2, ^2^ *J* C—F = 7.04 Hz). NMR-^31^P­{^1^H} (acetone-*d*_6_) [δ ppm, (multiplicity, assignation)]: 10.27 (d, PPh_3_^b^, ^1^ *J* P_b_-Pt = 3651.72 Hz); 8.22 (d, PPh_3_^a^, ^1^ *J* P_a_-Pt = 3385.09 Hz); ^2^ *J* P_a_-P_b_ = 25.75 Hz; −144.5 (PF_6_, sept, ^1^ *J* P—F = 711.24 Hz). ESI­(+)-MS-MS (acetone:water): [M – PF_6_]^+^ (1096.2580 *m*/*z*, 55%), [M – PF_6_ – MQ + H]^+^ (720.1563 *m*/*z*, 100%). Molar conductivity (DMSO): 27.10 ± 0.01 S cm^2^ mol^–1^.

##### [Pt­(dppm)­(MQ)]­PF_6_ (**2**)

A white solid was obtained with a yield of 91.3% (174 mg). Elemental analysis (%) Calc. for C_42_H_37_F_12_N_2_OP_3_Pt: C 45.79; H 3.39; N 2.54. Found: C 45.57; H 3.12; N 2.51. IR [*v*_max_ cm^–1^ (assignation)]: ν 3250 (N—H), ν 3057 (C—H aromatic), ν 2945 (C—H aliphatic), ν 1618 (C=C), ν 1599 and 1585 (C=N), ν 1107 (P—C), 847 (PF_6_), δ 692 (Ph—P—Ph), ν 557 (Pt—P). UV–vis (DMSO) [Log ε, λ (assignation)]: 4.21 M^–1^ cm^–1^, 264 nm (π–π* Ph); 4.17 M^–1^ cm^–1^, 268 nm (M → L); 4.12 M^–1^ cm^–1^, 276 nm (L → M); 4.04 M^–1^ cm^–1^, 286 nm (L → M and n−π* MQ); 3.85 and 3.68 M^–1^ cm^–1^, 304 and 316 nm (π–π* MQ). NMR-^1^H (acetone-*d*_6_) [δ ppm, (integral; multiplicity; assignation, *J* Hz)]: 0.76; 2.01 (2H, d, H6′, ^3^ *J* = 12.96 Hz), 0.89;0.95 (2H, m, H5′), 1.10;1.41 (2H, d, H4′, ^3^ *J* = 12.44 Hz), 3.20;3.59 (2H, d, H3′, ^3^ *J* = 11.20 Hz), 3.56 (1H, m, H1′), 5.26 (2H, m, He), 6.21 (1H, d, H1″, ^3^ *J* = 2.40 Hz), 6.66 (1H, bs, NH), 7.41; 7.57; 7.69; 7.80 (8H, m, Hc), 7.50; 7.62; 7.76; 7.78 (4H, m, Hd), 7.94 (1H, m, H6), 7.99; 8.17; 8.53 (8H, m, Hb), 8.33 (1H, d, H5, ^3^ *J*_*ortho*_ = 7.24 Hz), 8.51 (1H, s, H3), 8.56 (1H, d, H7, ^3^ *J*_*ortho*_ = 7.92 Hz). NMR-^13^C­{^1^H} (acetone-*d*_6_) [δ ppm, (multiplicity; assignation, *J* Hz)]: 22.7 (C4′), 23.2 (C6′), 23.9 (C5′), 42.8 (t, Ce, ^1^ *J* C—P = 34.88 Hz), 53.2 (C3′), 67.3 (C1′), 81.8 (C1″), 117.1 (C3), 123.8 (C9), 125.8 (C10), 127.3; 128.1 (Ca), 128.4 (C4), 128.5 (C6), 129.4 (C12), 129.4 (C7), 130.2 (C5), 130.1; 130.6; 132.8; 133.4 (d, Cc, ^3^ *J* C—P = 11.32 Hz), 133.3; 134.0, 134.50 (Cd), 133.8; 135.4 (Cb, ^2^ *J* C—P = 12.15; 12.90 Hz), 144.0 (C8), 148.6 (q, C11, ^1^ *J* C—F = 34.56 Hz), 154.2 (d, C2, ^2^ *J* C—F = 6.20 Hz). NMR-^31^P­{^1^H} (acetone-*d*_6_) [δ ppm, (multiplicity, assignation)]: −54.73 (d, dppm^b^, ^1^ *J* P_b_-Pt = 2971.33 Hz); −57.84 (d, dppm^a^, ^1^ *J* P_a_-Pt = 2592.44 Hz); ^2^ *J* P_a_-P_b_ = 74.34 Hz; −144.5 (PF_6_, sept, ^1^ *J* P—F = 711.24 Hz). ESI­(+)-MS-MS (acetone:water): [M – PF_6_]^+^ (956.2017 *m*/*z*, 100%), [M – PF_6_ – MQ]^+^ (579.0988 *m*/*z*, 72.13%). Molar conductivity (DMSO): 28.80 ± 0.01 S cm^2^ mol^–1^.

##### [Pt­(dppe)­(MQ)]­PF_6_·^1^/_2_H_2_O (**3**)

A white solid was obtained with a yield of 87.9% (148 mg). Elemental analysis (%) Calc. for C_43_H_39_F_12_N_2_OP_3_Pt·^1^/_2_H_2_O: C 46.10; H 3.55; N 2.50. Found: C 46.29; H 3.52; N 2.51. IR [*v*_max_ cm^–1^ (assignation)]: ν 3246 (N—H), ν 3059 (C—H aromatic), ν 2943 (C—H aliphatic), ν 1622 (C=C), ν 1599 and 1585 (C=N), ν 1109 (P—C), 845 (PF_6_), ν 557 (Pt—P). UV–vis (DMSO) [Log ε, λ (assignation)]: 4.12 M^–1^ cm^–1^, 264 nm (π–π* Ph); 4.10 M^–1^ cm^–1^, 270 nm (M → L); 4.10 M^–1^ cm^–1^, 278 nm (L → M); 4.04 M^–1^ cm^–1^, 286 nm (L → M and n−π* MQ); 3.86 and 3.71 M^–1^ cm^–1^, 304 and 316 nm (π–π* MQ). NMR-^1^H (acetone-*d*_6_) [δ ppm, (integral; multiplicity; assignation, *J* Hz)]: 0.66; 1.83:1.21; 1.72 (2H, d, H6′, ^3^ *J* = 13.12; 12.40 Hz), 0.78; 0.85:1.38 (2H, m, H5′), 1.00; 1.37:1.29 (2H, m, H4′), 2.56; 2.98:2.91; 3.34 (2H, m, H3′), 3.02 (4H, m, He), 2.97:3.50 (1H, m, H1′), 5.59:6.53 (1H, d;bs, H1″, ^3^ *J*_*ortho*_ = 3.97 Hz), 7.32; 7.60 (4H, m, Hd), 7.33; 7.74 (8H, m, Hc), 7.52:7.88 (1H, m:d, H6, ^3^ *J*_*ortho*_ = 7.90 Hz), 8.18:8.21 (1H, s, H3), 7.65; 8.15; 8.38 (8H, m, Hb), 8.06:8.30 (1H, d, H5, ^3^ *J*_*ortho*_ = 6.73:6.97 Hz), 8.68:8.82 (1H, d, H7, ^3^ *J*_*ortho*_ = 8.36:7.90 Hz). NMR-^13^C­{^1^H} (acetone-*d*_6_) [δ ppm, (multiplicity; assignation, *J* Hz)]: 23.1:25.1 (C6′), 23.7:27.2 (C5′), 23.1:27.0 (C4′), 26.0;32.0 (dd, Ce, ^1^ *J* C—P = 43.50; 42.50 Hz and ^3^ *J* C—P = 7.56; 8.15 Hz), 47.5:52.1 (C3′), 62.2:67.7 (C1′), 73.2:80.6 (C1″), 116.5:116.9 (C3), 121.2 (C9), 123.5 (C10), 126.5 (d, Ca, ^1^ *J* C—P = 56.27 Hz), 128.1 (C6), 129.2 (C12), 129.7:130.0 (C5), 129.9; 130.1; 130.7 (d, Cc, ^3^ *J* C—P = 11.52 Hz), 130.1:130.2 (C7), 130.2 (C4), 132.7; 133.0; 133.2 (Cd), 133.8; 133.9; 134.2; 135.6 (d, Cb, ^3^ *J* C—P = 11.52 Hz), 143.7:144.3 (C8), 148.3:148.4 (q, C11, ^1^ *J* C—F = 34.94 Hz), 154.3:154.9 (s:d, C2, ^2^ *J* C—F = 5.98 Hz). NMR-^31^P­{^1^H} (acetone-*d*_6_) [δ ppm, (multiplicity, assignation)]: 36.10 (d, dppe^b^, ^1^ *J* P_b_-Pt = 3423.50 Hz); 32.65 (d, dppe^a^, ^1^ *J* P_a_-Pt = 3304.84 Hz); ^2^ *J* P_a_-P_b_ = 6.04 Hz; −144.5 (PF_6_, sept, ^1^ *J* P—F = 711.24 Hz). ESI­(+)-MS-MS (acetone:water): [M – PF_6_]^+^ (970.2026 *m*/*z*, 100%), [M – PF_6_ – MQ]^+^ (593.0976 *m*/*z*, 61.40%). Molar conductivity (DMSO): 25.90 ± 0.01 S cm^2^ mol^–1^.

##### [Pt­(dppp)­(MQ)]­PF_6_ (**4**)

A white solid was obtained with a yield of 93.1% (163 mg). Elemental analysis (%) Calc. for C_44_H_41_F_12_N_2_OP_3_Pt: C 46.78; H 3.66; N 2.48. Found: C 46.65; H 3.40; N 2.44. IR [*v*_max_ cm^–1^ (assignation)]: ν 3246 (N—H), ν 3059 (C—H aromatic), ν 2943 (C—H aliphatic), ν 1624 (C=C), ν 1597 and 1585 (C=N), ν 1105 (P—C), 845 (PF_6_), ν 557 (Pt—P). UV–vis (DMSO) [Log ε, λ (assignation)]: 4.12 M^–1^ cm^–1^, 264 nm (π–π* Ph); 4.11 M^–1^ cm^–1^, 270 nm (M → L); 4.09 M^–1^ cm^–1^, 274 nm (L → M); 3.97 M^–1^ cm^–1^, 286 nm (L → M and n−π* MQ); 3.78 and 3.63 M^–1^ cm^–1^, 304 and 316 nm (π–π* MQ). NMR-^1^H (acetone-*d*_6_) [δ ppm, (integral; multiplicity; assignation, *J* Hz)]: 0.66; 1.99 (2H, d, H6′, ^3^ *J* = 13.48 Hz), 1.04; 1.51 (2H, m, H4′), 1.07; 1.58 (2H, m, H5′), 1.95; 2.73 (2H, m, H3′), 2.09; 2.27 (2H, m, Hf), 2.79; 2.99; 3.18 (4H, m, He), 3.50 (1H, d, H1′, ^3^ *J* = 10.40 Hz), 5.99 (1H, bs, H1″), 6.10 (1H, bs, NH), 7.43; 7.60 (8H, m, Hc), 7.45 (1H, s, H3), 7.51; 7.70 (4H, m, Hd), 7.86 (1H, m, H6), 7.90 (8H, m, Hb), 8.25 (1H, d, H5, ^3^ *J*_*ortho*_ = 7.2 Hz), 8.43 (1H, d, H7, ^3^ *J*_*ortho*_ = 8.60 Hz). NMR-^13^C­{^1^H} (acetone-*d*_6_) [δ ppm, (multiplicity; assignation, *J* Hz)]: 19.6 (Cf), 23.1 (C6′), 23.3 (C5′), 25.4 (C4′), 23.0;26.4 (dd, Ce, ^1^ *J* C—P = 40.34 Hz and ^3^ *J* C—P = 5.92 Hz), 49.9 (C3′), 68.7 (C1′), 79.8 (C1″), 117.1 (C3), 126.8 (C9), 127.9 (C10), 128.2 (C6), 128.5; 128.9 (Ca), 129.3 (C12), 129.4 (C7), 129.5; 130.2; 132.6; 133.1 (Cc), 129.8 (C5), 130.2; 132.6 (Cd), 133.6; 134.4 (Cb), 143.6 (C8), 148.4 (q, C11, ^1^ *J* C—F = 34.52 Hz), 153.7 (d, C2, ^2^ *J* C—F = 6.38 Hz). NMR-^31^P­{^1^H} (acetone-*d*_6_) [δ ppm, (multiplicity, assignation)]: −7.8 (d, dppp^b^, ^1^ *J* P_b_-Pt = 3327.60 Hz); −11.1 (d, dppp^a^, ^1^ *J* P_a_-Pt = 3057.48 Hz); ^2^ *J* P_a_-P_b_ = 36.12 Hz; −144.5 (PF_6_, sept, ^1^ *J* P—F = 711.24 Hz. ESI­(+)-MS-MS (acetone:water): [M – PF_6_]^+^ (984.2216 *m*/*z*, 100%), [M – PF_6_ – MQ]^+^ (606.1055 *m*/*z*, 51.80%). Molar conductivity (DMSO): 27.83 ± 0.01 S cm^2^ mol^–1^.

##### [Pt­(dppb)­(MQ)]­PF_6_ (**5**)

A white solid was obtained with a yield of 85.7% (142 mg). Elemental analysis (%) Calc. for C_45_H_43_F_12_N_2_OP_3_Pt: C 47.25; H 3.79; N 2.45. Found: C 47.16; H 3.72; N 2.47. IR [*v*_max_ cm^–1^ (assignation)]: ν 3252 (N—H), ν 3059 (C—H aromatic), ν 2943 (C—H aliphatic), ν 1624 (C=C), ν 1597 and 1585 (C=N), ν 1105 (P—C), 843 (PF_6_), ν 557 (Pt—P). UV–vis (DMSO) [Log ε, λ (assignation)]: 4.10 M^–1^ cm^–1^, 264 nm (π–π* Ph); 4.09 M^–1^ cm^–1^, 270 nm (M → L); 4.08 M^–1^ cm^–1^, 274 nm (L → M); 3.95 M^–1^ cm^–1^, 286 nm (L → M and n−π* MQ); 3.78 and 3.63 M^–1^ cm^–1^, 304 and 316 nm (π–π* MQ). NMR-^1^H (acetone-*d*_6_) [δ ppm, (integral; multiplicity; assignation, *J* Hz)]: 0.68; 1.95 (2H, d, H6′, ^3^ *J* = 12.76; 12.52 Hz), 1.08; 1.58 (2H, d, H5′, ^3^ *J* = 13.08; 11.20 Hz), 1.17; 1.45 (2H, d, H4′, ^3^ *J* = 12.96;13.20 Hz), 1.79; 2.64 (2H, m, H3′), 1.83; 2.30 (4H, m, Hf), 2.61; 3.04; 3.24 (4H, m, He), 3.46 (1H, d, H1′, ^3^ *J* = 8.32 Hz), 5.85 (1H, bs, NH), 6.03 (1H, bs, H1″), 7.46; 7.66 (8H, m, Hc), 7.54 (1H, s, H3), 7.58; 7.72 (4H, m, Hd), 7.86 (1H, m, H6), 7.86; 7.99 (8H, m, Hb), 8.26 (1H, d, H5, ^3^ *J*_*ortho*_ = 6.76 Hz), 8.46 (1H, d, H7, ^3^ *J*_*ortho*_ = 8.52 Hz). NMR-^13^C­{^1^H} (acetone-*d*_6_) [δ ppm, (multiplicity; assignation, *J* Hz)]: 22.7 (Cf), 23.0 (C6′), 23.3 (C5′), 25.5 (C4′), 25.4; 28.9 (d, Ce, ^1^ *J* C—P = 37.39 Hz), 49.3 (C3′), 68.4 (C1′), 79.9 (C1″), 117.1 (C3), 123.6 (C9), 126.3 (C10), 128.3 (d, Ca, ^1^ *J* C—P = 65.10 Hz), 128.3 (C6), 129.3 (C12), 129.3 (C7), 129.5;130.51 (Cc), 129.9 (C5), 132.5; 132.7; 133.2 (Cd), 133.4; 133.7; 134.2 (Cb), 143.6 (C8), 148.4 (q, C11, ^1^ *J* C—F = 34.76 Hz), 153.5 (d, C2, ^2^ *J* C—F = 6.68 Hz). NMR-^31^P­{^1^H} (acetone-*d*_6_) [δ ppm, (multiplicity, assignation)]: 8.42 (d, dppb^b^, ^1^ *J* P_b_-Pt = 3220.43 Hz), 0.59 (d, dppb^a^, ^1^ *J* P_a_-Pt = 3427.06 Hz); ^2^ *J* P_a_-P_b_ = 28.35 Hz; −144.5 (PF_6_, sept, ^1^ *J* P—F = 711.24 Hz). ESI­(+)-MS-MS (acetone:water): [M – PF_6_]^+^ (998.2403 *m*/*z*, 52.70%), [M – PF_6_ – MQ]^+^ (620.1232 *m*/*z*, 100%). Molar conductivity (DMSO): 28.52 ± 0.01 S cm^2^ mol^–1^.

##### [Pt­(dppf)­(MQ)]­PF_6_ (**6**)

A dark yellow solid was obtained with a yield of 88.6% (145 mg). Elemental analysis (%) Calc. for C_51_H_43_F_12_FeN_2_OP_3_Pt: C 48.17; H 3.41; N 2.20. Found: C 47.87; H 3.34; N 2.24. IR [*v*_max_ cm^–1^ (assignation)]: ν 3248 (N—H), ν 3059 (C—H aromatic), ν 2951 (C—H aliphatic), ν 1620 (C=C), ν 1597 and 1587 (C=N), ν 1105 (P—C), 847 (PF_6_), ν 559 (Pt—P). UV–vis (DMSO) [Log ε, λ (assignation)]: 4.23 M^–1^ cm^–1^, 264 nm (π–π* Ph); 4.19 M^–1^ cm^–1^, 270 nm (M → L); 4.14 M^–1^ cm^–1^, 276 nm (L → M); 4.03 M^–1^ cm^–1^, 286 nm (L → M and n−π* MQ); 3.84 and 3.72 M^–1^ cm^–1^, 304 and 316 nm (π–π* MQ); 3.06 M^–1^ cm^–1^, 346 nm (*d*–*d* Pt); 2.35 M^–1^ cm^–1^, 434 nm (*d*–*d* ferrocene). NMR-^1^H (acetone-*d*_6_) [δ ppm, (integral; multiplicity; assignation, *J* Hz)]: 0.71; 2.22 (2H, d, H6′, ^3^ *J* = 13.30 Hz), 1.17; 1.75 (2H, d, H5′, ^3^ *J* = 13.30 Hz), 1.24; 1.78 (2H, d, H4′, ^3^ *J* = 13.80 Hz), 1.45; 2.51 (2H, d, H3′, ^3^ *J* = 12.76 Hz), 3.31; 3.75; 4.77; 5.06 (4H, bs, Hf), 3.49 (1H, m, H1′), 4.49; 4.50; 5.06; 5.77 (4H, bs, Hg), 6.07 (1H, bs, H1″), 6.13 (1H, bs, NH), 7.03 (1H, s, H3), 7.26; 7.50; 7.68 (8H, m, Hc), 7.46; 7.54; 7.77 (4H, m, Hd), 7.58; 8.03; 8.14 (8H, m, Hb), 7.86 (1H, t, H6, ^3^ *J*_*ortho*_ *=* 7.83 Hz), 8.26 (1H, d, H5, ^3^ *J*_*ortho*_ = 7.83 Hz), 8.47 (1H, d, H7, ^3^ *J*_*ortho*_ = 7.83 Hz). NMR-^13^C­{^1^H} (acetone-*d*_6_) [δ ppm, (multiplicity; assignation, *J* Hz)]: 22.9; 23.0 (C6′), 23.6 (C5′), 26.1; 26.2 (C4′), 48.8; 48.9 (C3′), 68.6; 68.8 (C1′), 69.6; 71.4 (d, Ce, ^1^ *J* C—P = 69.71 Hz), 73.8; 75.9; 76.1; 76.8 (d, Cg, ^3^ *J* C—P *=* 7.58; 7.58; 5.89; 5.05 Hz), 75.5; 77.3; 78.3; 81.0 (d, Cf, ^2^ *J* C—P *=* 10.11; 12.63; 8.42; 20.22 Hz), 79.8 (C1″), 117.0 (C3), 120.8 (C9), 123.5 (C10), 128.2 (C4), 128.3 (C6), 129.3 (C7), 129.3 (C12), 129.4; 130.3; 130.4 (Cc), 129.8; 134.0; 135.9; 136.5 (Cb), 129.9 (C5), 132.7; 132.8; 133.0; 133.4 (Cd), 130.5; 132.8 (d, Ca, ^1^ *J* C—P *=* 67.80 Hz), 143.5 (C8), 148.4 (q, C11, ^1^ *J* C—F = 34.80 Hz), 152.9 (d, C2, ^2^ *J* C—F = 6.87 Hz). NMR-^31^P­{^1^H} (acetone-*d*_6_) [δ ppm, (multiplicity, assignation)]: 9.84 (d, dppf^b^, ^1^ *J* P_b_-Pt = 3733.96 Hz); 8.40 (d, dppf^a^, ^1^ *J* P_a_-Pt = 3443.05 Hz); ^2^ *J* P_a_-P_b_ = 25.83 Hz; −144.5 (PF_6_, sept, ^1^ *J* P—F = 711.24 Hz). ESI­(+)-MS-MS (acetone:water): [M – PF_6_]^+^ (1126.1756 *m*/*z*, 85.70%), [M – PF_6_ – MQ]^+^ (748.0340 *m*/*z*, 100%). Molar conductivity (DMSO): 26.94 ± 0.01 S cm^2^ mol^–1^.

##### *cis*-[Pt­(PTA)_2_(MQ)]­PF_6_·2H_2_O (**7**)

A pale gray solid was obtained with a yield of 83.4% (138 mg). Elemental analysis (%) Calc. for C_29_H_39_F_12_N_8_OP_3_Pt·2H_2_O: C 32.62; H 10.50; N 4.06. Found: C 32.49; H 10.54; N 4.03. IR [*v*_max_ cm^–1^ (assignation)]: ν 3272 (N—H), ν 3079 (C—H aromatic), ν 2946 (C—H aliphatic), ν 1630 (C=C), ν 1601 and 1585 (C=N), ν 1107 (P—C), 846 (PF_6_), ν 592 and 582 (Pt—P). UV–vis (DMSO) [Log ε, λ (assignation)]: 4.08 M^–1^ cm^–1^, 264 nm; 4.11 M^–1^ cm^–1^, 280 nm (L → M); 3.88 and 3.71 M^–1^ cm^–1^, 304 and 316 nm (π–π* MQ). NMR-^1^H (DMSO-*d*_6_) [δ ppm, (integral; multiplicity; assignation, *J* Hz)]: 0.52; 1.77 (2H, d, H6′, ^3^ *J* = 12.00 Hz), 1.12; 1.55 (2H, d, H5′, ^3^ *J* = 12.00 Hz), 1.42; 1.75 (2H, m, H4′), 3.29 (2H, m, H3′), 3.38 (1H, m, H1′), 4.44 (12H, bs, Ha), 4.46 (12H, bs, Hb), 5.57 (1H, d, H1″, ^3^ *J* = 1.85 Hz), 6.96 (1H, bs, NH), 7.89 (1H, t, H6, ^3^ *J* = 8.00 Hz), 8.09 (1H, s, H3), 8.29 (1H, d, H5, ^3^ *J*_*ortho*_ = 8.00 Hz), 8.33 (1H, d, H7, ^3^ *J*_*ortho*_ = 8.00 Hz). NMR-^13^C­{^1^H} (DMSO-*d*_6_) [δ ppm, (multiplicity; assignation, *J* Hz)]: 21.5 (C6′), 21.8 (C5′), 24.4 (C4′), 49.6 (t, Ca, ^1^ *J* C—P = 21.25 Hz), 51.9 (C1′), 67.2 (C3′), 71.2 (dd, Cb, *J* C—P = 26.85; 7.83 Hz), 78.5 (C1″), 115.4 (C3), 122.2 (C9), 124.9 (C10), 126.7 (C4), 126.9 (C12), 128.0 (C6), 128.3 (C7), 129.7 (C5), 142.3 (C8), 146.8 (q, C11, ^1^ *J* C—F = 34.37 Hz), 152.2 (d, C2, ^2^ *J* C—F = 6.71 Hz). NMR-^31^P­{^1^H} (DMSO-*d*_6_) [δ ppm, (multiplicity, assignation)]: −59.74 (d, PTA^b^, ^1^ *J* P_b_-Pt = 3131.29 Hz); −71.29 (d, PTA^a^, ^1^ *J* P_a_-Pt = 3016.57 Hz); ^2^ *J* P_a_-P_b_ = 22.92 Hz; −144.5 (PF_6_, sept, ^1^ *J* P—F = 711.24 Hz). ESI­(+)-MS (acetonitrile:water): [M – PF_6_]^+^ (886.2627 *m*/*z*, 15.42%), [MQ + H]^+^ (379.1273 *m*/*z*, 100%). ESI­(+)-MS-MS (acetonitrile:water): [M – PF_6_]^+^ (886.2627 *m*/*z*, 100%), [M – PF_6_ – PTA]^+^ (729.1564 *m*/*z*, 5.12%), [M – PF_6_ – 2PTA]^+^ (510.1266 *m*/*z*, 28.30%). Molar conductivity (DMSO): 32.60 ± 0.01 S cm^2^ mol^–1^.

##### [Pd­(dppm)­(MQ)]­PF_6_·2H_2_O (**8**)

A pale orange solid was obtained with a yield of 87.8% (120 mg). Elemental analysis (%) Calc. for C_42_H_37_F_12_N_2_OP_3_Pd·2H_2_O: C 48.08; H 3.94; N 2.67. Found: C 48.28; H 3.84; N 3.07. IR [*v*_max_ cm^–1^ (assignation)]: ν 3261 (N—H), ν 3060 (C—H aroma′tico), ν 2944 (C—H alifa′tico), ν 1618 (C=C), ν 1601 and 1585 (C=N), ν 1108 (P—C), 848 (PF_6_), ν 560 (Pd—P). UV–vis (DMSO) [Log ε, λ (assignation)]: 4.28 M^–1^ cm^–1^, 268 nm (π–π* Ph); 4.25 M^–1^ cm^–1^, 278 nm (L → M); 4.21 M^–1^ cm^–1^, 290 nm (L → M and n−π* MQ); 4.13 and 4.00 M^–1^ cm^–1^, 304 and 316 nm (π–π* MQ); 2.97 M^–1^ cm^–1^, 410 nm (*d*–*d* Pd). NMR-^1^H (acetone-*d*_6_) [δ ppm, (integral; multiplicity; assignation, *J* Hz)]: 0.79; 2.11:1.86 (2H, d:m, H6′, ^3^ *J* = 13.16 Hz), 0.97:1.06; 1.46 (2H, m, H5′), 1.52; 1.91 (2H, m, H4′), 2.97; 3.06:3.46; 3.81 (2H, m:d, H3′, ^3^ *J* = 12.85 Hz), 3.55:4.09 (1H, m:d, H1′, ^3^ *J* = 12.86 Hz), 4.88; 5.09 (2H, m, He), 6.23 (1H, bs, NH), 6.24:6.27 (1H, d, H1″, ^3^ *J* = 2.84 Hz), 7.38; 7.57; 7.64; 7.76 (8H, m, Hc), 7.48; 7.69; 7.85 (4H, m, Hd), 7.66; 8.00; 8.16; 8.55 (8H, m, Hb), 7.92:7.98 (1H, t, H6, ^3^ *J*_*ortho*_ = 7.97 Hz), 8.26:8.50 (1H, s, H3), 8.31:8.37 (1H, d, H5, ^3^ *J*_*ortho*_ = 7.97 Hz), 8.59:8.66 (1H, d, H7, ^3^ *J*_*ortho*_ = 7.97 Hz). NMR-^13^C­{^1^H} (acetone-*d*_6_) [δ ppm, (multiplicity; assignation, *J* Hz)]: 22.3:23.9 (C6′), 22.6:24.8 (C5′), 22.9 (C4′), 36.9 (t, Ce, ^1^ *J* C—P = 28.33 Hz), 47.0:52.1 (C3′), 61.1:67.9 (C1′), 69.4:80.2 (C1″), 116.6:117.3 (C3), 121.4 (C9), 123.7 (C10), 126.1 (d, Ca, ^1^ *J* C—P = 49.31 Hz), 127.5:128.9 (C4), 128.3:129.2 (C6), 129.0:129.6 (C7), 130.0 (C5), 130.2; 130.5; 130.7; 130.8 (d, Cc, ^3^ *J* C—P = 12.39 Hz), 133.0; 133.7; 134.0; 135.6 (Cb, ^2^ *J* C—P = 11.80; 12.96; 12.39; 12.98 Hz), 133.3; 134.50 (Cd), 143.9:144.3 (C8), 148.5 (q, C11, ^1^ *J* C—F = 34.50 Hz), 150.6:155.3 (s:d, C2, ^2^ *J* C—F = 6.90 Hz). NMR-^31^P­{^1^H} (acetone-*d*_6_) [δ ppm, (multiplicity, assignation)]: −37.56 (d, dppm^b^); −40.58 (d, dppm^a^); ^2^ *J* P_a_-P_b_ = 111.28 Hz; −144.5 (PF_6_, sept, ^1^ *J* P—F = 711.24 Hz). ESI­(+)-MS (acetone:water): [M – PF_6_]^+^ (867.1231 *m*/*z*, 8,10%), [MQ + H]^+^ (379.1249 *m*/*z*, 100%). Molar conductivity (DMSO): 42.68 ± 0.01 S cm^2^ mol^–1^.

##### [Pd­(dppe)­(MQ)]­PF_6_ (**9**)

A white solid was obtained with a yield of 93.4% (164 mg). Elemental analysis (%) Calc. for C_43_H_39_F_12_N_2_OP_3_Pd: C 50.28; H 3.83; N 2.73. Found: C 50.13; H 3.74; N 2.96. IR [*v*_max_ cm^–1^ (assignation)]: ν 3258 (N—H), ν 3059 (C—H aromatic), ν 2942 (C—H aliphatic), ν 1617 (C=C), ν 1597 and 1586 (C=N), ν 1107 (P—C), 846 (PF_6_), ν 558 (Pd—P). UV–vis (DMSO) [Log ε, λ (assignation)]: 4.45 M^–1^ cm^–1^, 266 nm (π–π* Ph); 4.41 M^–1^ cm^–1^, 276 nm (L → M); 4.40 M^–1^ cm^–1^, 286 nm (L → M and n−π* MQ); 4.03 M^–1^ cm^–1^, 318 nm (π–π* MQ). NMR-^1^H (acetone-*d*_6_) [δ ppm, (integral; multiplicity; assignation, *J* Hz)]: 0.78; 1.95 (2H, d, H6′, ^3^ *J* = 13.91 Hz), 1.00 (2H, m, H5′), 1.05; 1.51 (2H, m, H4′), 2.53; 2.83 (2H, d, H3′, ^3^ *J* = 11.38 Hz), 2.99; 3.20 (4H, m, He), 3.58 (1H, m, H1′), 6.12 (1H, bs, NH), 6.22 (1H, bs, H1″), 7.48; 7.72 (8H, m, Hc), 7.55; 7.78; 7.81 (4H, m, Hd), 7.89 (1H, m, H6, ^3^ *J*_*ortho*_ = 7.85 Hz), 7.96; 8.07; 8.17; 8.30 (8H, m, Hb), 8.25 (1H, s, H3), 8.32 (1H, m, H5), 8.58 (1H, d, H7, ^3^ *J*_*ortho*_ = 7.85 Hz). NMR-^13^C­{^1^H} (acetone-*d*_6_) [δ ppm, (multiplicity; assignation, *J* Hz)]: 23.1 (C4′), 24.1 (C6′), 24.9 (C5′), 31.4 (dd, Ce, ^1^ *J* C—P = 36.17 Hz and ^2^ *J* C—P = 11.90 Hz), 50.9 (C3′), 68.7 (C1′), 79.6 (C1″), 117.3 (C3), 121.3 (C9), 123.5 (C10), 127.3; 128.7 (d, Ca, ^1^ *J* C—P = 49.86 Hz), 128.3 (C6), 129.0 (C12), 129.3 (C4), 129.6 (C7), 129.9 (C5), 130.0; 130.7; 130.9 (d, Cc, ^3^ *J* C—P = 11.03 Hz), 133.1; 133.4; 133.7; 134.0 (Cd), 133.9; 134.4; 134.5; 134.7 (Cb, ^2^ *J* C—P = 11.03; 11.51; 11.99 Hz), 143.8 (C8), 148.5 (q, C11, ^1^ *J* C—F = 34.50 Hz), 155.2 (d, C2, ^2^ *J* C—F = 7.20 Hz). NMR-^31^P­{^1^H} (acetone-*d*_6_) [δ ppm, (multiplicity, assignation)]: 58.35 (d, dppe^b^); 53.94 (d, dppe^a^); ^2^ *J* P_a_-P_b_ = 29.56 Hz; −144.5 (PF_6_, sept, ^1^ *J* P—F = 711.24 Hz). ESI­(+)-MS (acetone:water): [M – PF_6_]^+^ (881.1485 *m*/*z*, 7%), [MQ + H]^+^ (379.1243 *m*/*z*, 100%). Molar conductivity (DMSO): 24.63 ± 0.01 S cm^2^ mol^–1^.

##### [Pd­(dppp)­(MQ)]­PF_6_·^1^/_2_H_2_O (**10**)

A white solid was obtained with a yield of 91.2% (127 mg). Elemental analysis (%) Calc. for C_44_H_41_F_12_N_2_OP_3_Pd·^1^/_2_H_2_O: C 50.32; H 4.03; N 2.67. Found: C 49.89; H 3.94; N 2.67. IR [*v*_max_ cm^–1^ (assignation)]: ν 3258 (N—H), ν 3060 (C—H aromatic), ν 2942 (C—H aliphatic), ν 1621 (C=C), ν 1596 and 1585 (C=N), ν 1105 (P—C), 844 (PF_6_), ν 558 (Pd—P). UV–vis (DMSO) [Log ε, λ (assignation)]: 4.40 M^–1^ cm^–1^, 266 nm (π–π* Ph); 4.39 M^–1^ cm^–1^, 278 nm (L → M); 4.37 M^–1^ cm^–1^, 290 nm (L → M and n−π* MQ); 4.04 M^–1^ cm^–1^, 316 nm (π–π* MQ). NMR-^1^H (acetone-*d*_6_) [δ ppm, (integral; multiplicity; assignation, *J* Hz)]: 0.67; 1.97 (2H, d, H6′, ^3^ *J* = 13.70 Hz), 1.01; 1.32 (2H, d, H4′, ^3^ *J* = 12.70 Hz), 1.04; 1.57 (2H, d, H5′, ^3^ *J* = 12.70 Hz), 1.65; 2.36 (2H, d, H3′, ^3^ *J* = 11.70 Hz), 1.98; 2.47 (2H, m, Hf), 2.76; 2.93; 3.14 (4H, m, He), 3.46 (1H, d, H1′, ^3^ *J* = 9.78 Hz), 5.75 (1H, bs, NH), 6.07 (1H, d, H1″, ^3^ *J* = 2.73 Hz), 7.39 (1H, s, H3), 7.42; 7.48; 7.55; 7.60 (8H, m, Hc), 7.49; 7.58; 7.62; 7.69 (4H, m, Hd), 7.74; 7.86; 7.95; 8.00 (8H, m, Hb), 7.84 (1H, m, H6), 8.24 (1H, d, H5, ^3^ *J*_*ortho*_ = 7.23 Hz), 8.49 (1H, d, H7, ^3^ *J*_*ortho*_ = 8.48 Hz). NMR-^13^C­{^1^H} (acetone-*d*_6_) [δ ppm, (multiplicity; assignation, *J* Hz)]: 19.5 (Cf), 23.4 (C5′), 23.8 (C6′), 23.9;26.1 (dd, Ce, ^1^ *J* C—P = 32.47 Hz and ^3^ *J* C—P = 7.28 Hz), 26.1 (C4′), 49.1 (C3′), 69.2 (C1′), 79.0 (C1″), 117.2 (C3), 121.0 (C9), 123.8 (C10), 127.4; 128.0 (d, Ca, ^1^ *J* C—P = 39.65 Hz), 128.1 (C6), 129.0 (q, C12, ^1^ *J* C—F = 30.22 Hz), 129.2 (C4), 129.4; 129.8; 130.3 (d, Cc, ^3^ *J* C—P = 12.10; 11.30; 10.87 Hz), 129.5 (C7), 129.7 (C5), 132.4; 132.7; 132.9; 133.2 (Cd), 133.6; 133.9; 134.7; 135.1 (d, Cb, ^2^ *J* C—P = 10.87; 10.43; 11.30 Hz), 143.5 (C8), 148.4 (q, C11, ^1^ *J* C—F = 33.90 Hz), 154.6 (d, C2, ^2^ *J* C—F = 7.63 Hz). NMR-^31^P­{^1^H} (acetone-*d*_6_) [δ ppm, (multiplicity, assignation)]: 12.94 (d, dppp^b^); 7.26 (d, dppp^a^); ^2^ *J* P_a_-P_b_ = 57.50 Hz; −144.5 (PF_6_, sept, ^1^ *J* P—F = 711.24 Hz). ESI­(+)-MS (acetone:water): [M – PF_6_]^+^ (895.1617 *m*/*z*, 10%), [MQ + H]^+^ (379.1243 *m*/*z*, 100%). Molar conductivity (DMSO): 28.54 ± 0.01 S cm^2^ mol^–1^.

##### [Pd­(dppb)­(MQ)]­PF_6_ (**11**)

A white solid was obtained with a yield of 86.3% (110 mg). Elemental analysis (%) Calc. for C_45_H_43_F_12_N_2_OP_3_Pd: C 51.22; H 4.11; N 2.65. Found: C 51.68; H 4.23; N 2.97. IR [*v*_max_ cm^–1^ (assignation)]: ν 3277 (N—H), ν 3062 (C—H aromatic), ν 2940 (C—H aliphatic), ν 1619 (C=C), ν 1599 and 1586 (C=N), ν 1109 (P—C), 843 (PF_6_), ν 558 (Pd—P). UV–vis (DMSO) [Log ε, λ (assignation)]: 4.22 M^–1^ cm^–1^, 268 nm (π–π* Ph); 4.26 M^–1^ cm^–1^, 278 nm (L → M); 4.28 M^–1^ cm^–1^, 290 nm (L → M and n−π* MQ); 4.19 and 4.01 M^–1^ cm^–1^, 304 and 316 nm (π–π* MQ). NMR-^1^H (acetone-*d*_6_) [δ ppm, (integral; multiplicity; assignation, *J* Hz)]: 0.71;1.94 (2H, d; m, H6′, ^3^ *J* = 13.45 Hz), 1.03; 1.58 (2H, m; d, H4′, ^3^ *J* = 13.45 Hz), 1.17; 1.22 (2H, m, H5′), 1.33; 2.23 (2H, d; m, H3′, ^3^ *J* = 12.11 Hz), 1.80; 2.47 (4H, m, Hf), 2.40; 3.01; 3.19 (4H, m, He), 3.46 (1H, d, H1′, ^3^ *J* = 10.76 Hz), 5.56 (1H, bs, NH), 6.04 (1H, bs, H1″, ^3^ *J* = 2.50 Hz), 7.41; 7.48; 7.63; 7.66 (8H, m, Hc), 7.53; 7.62; 7.72 (4H, m, Hd), 7.61 (1H, s, H3), 7.84; 7.87; 7.94; 7.97 (8H, m, Hb), 7.85 (1H, m, H6), 8.25 (1H, d, H5, ^3^ *J*_*ortho*_ = 7.16 Hz), 8.50 (1H, d, H7, ^3^ *J*_*ortho*_ = 8.41 Hz). NMR-^13^C­{^1^H} (acetone-*d*_6_) [δ ppm, (multiplicity; assignation, *J* Hz)]: 22.9;26.3 (s; d, Cf, ^2^ *J* C—P = 7.39 Hz), 23.5 (C4′), 23.8 (C6′), 25.3; 28.5 (d, Ce, ^1^ *J* C—P = 30.80; 28.31 Hz), 26.5 (C5′), 48.1 (C3′), 69.0 (C1′), 79.1 (C1″), 117.2 (C3), 121.1 (C9), 123.8 (C10), 128.2 (C4), 128.3 (C6), 129.0 (q, C12, ^1^ *J* C—F = 29.42 Hz), 129.2; 130.0; 130.9; 132.2 (d, Ca, ^1^ *J* C—P = 48.45 Hz), 129.5 (C7), 129.4; 129.5; 130.5; 130.7 (d, Cc, ^3^ *J* C—P = 10.56 Hz), 129.8 (C5), 132.5; 132.6; 132.9; 133.2 (Cd), 133.8; 134.0; 134.2; 135.2 (d, Cb, ^2^ *J* C—P = 10.75; 10.00; 10.75; 10.00 Hz), 143.6 (C8), 148.4 (q, C11, ^1^ *J* C—F = 34.70 Hz), 154.3 (d, C2, ^2^ *J* C—F = 7.35 Hz). NMR-^31^P­{^1^H} (acetone-*d*_6_) [δ ppm, (multiplicity, assignation)]: 31.40 (d, dppb^b^); 21.93 (d, dppb^a^); ^2^ *J* P_a_-P_b_ = 47.21 Hz; −144.5 (PF_6_, sept, ^1^ *J* P—F = 711.24 Hz. ESI­(+)-MS (acetone:water): [M – PF_6_]^+^ (909.1796 *m*/*z*, 4%), [MQ + H]^+^ (379.1255 *m*/*z*, 100%). Molar conductivity (DMSO): 29.37 ± 0.01 S cm^2^ mol^–1^.

##### [Pd­(dppf)­(MQ)]­PF_6_ (**12**)

A dark red solid was obtained with a yield of 94.6% (154 mg). Elemental analysis (%) Calc. for C_51_H_43_F_12_FeN_2_OP_3_Pd: C 51.78; H 3.66; N 2.37. Found: C 51.87; H 3.69; N 2.40. IR [*v*_max_ cm^–1^ (assignation)]: ν 3262 (N—H), ν 3060 (C—H aromatic), ν 2942 (C—H aliphatic), ν 1619 (C=C), ν 1597 and 1587 (C=N), ν 1106 (P—C), 844 (PF_6_), ν 557 (Pd—P). UV–vis (DMSO) [Log ε, λ (assignation)]: 4.42 M^–1^ cm^–1^, 266 nm (π–π* Ph); 4.40 M^–1^ cm^–1^, 280 nm (L → M); 4.39 M^–1^ cm^–1^, 290 nm (L → M and n−π* MQ); 4.19 M^–1^ cm^–1^, 316 nm (π–π* MQ); 3.35 M^–1^ cm^–1^, 388 nm (*d*–*d* Pd); 3.14 M^–1^ cm^–1^, 446 nm (*d*–*d* ferrocene). NMR-^1^H (acetone-*d*_6_) [δ ppm, (integral; multiplicity; assignation, *J* Hz)]: 0.74; 2.24 (2H, d; m, H6′, ^3^ *J* = 13.41 Hz), 1.12; 1.76 (2H, m; d, H5′, ^3^ *J* = 12.77 Hz), 1.21; 2.16 (2H, m, H3′), 1.25; 1.58 (2H, m, H4′), 3.32; 3.73; 5.13; 5.74 (4H, bs, Hf), 3.53 (1H, m, H1′, ^3^ *J* = 11.00 Hz), 4.50; 4.55; 4.77; 5.12 (4H, bs, Hg), 5.75 (1H, bs, NH), 6.11 (1H, d, H1″, ^3^ *J* = 2.05 Hz), 7.05 (1H, s, H3), 7.29; 7.50; 7.54; 7.69 (8H, m, Hc), 7.62; 7.78 (4H, m, Hd), 7.95; 8.06; 8.13; 8.17 (8H, m, Hb), 7.85 (1H, t, H6, ^3^ *J*_*ortho*_ *=* 7.90 Hz), 8.25 (1H, d, H5, ^3^ *J*_*ortho*_ = 7.90 Hz), 8.50 (1H, d, H7, ^3^ *J*_*ortho*_ = 7.90 Hz). NMR-^13^C­{^1^H} (acetone-*d*_6_) [δ ppm, (multiplicity; assignation, *J* Hz)]: 23.7 (C6′), 23.7 (C5′), 27.0 (C4′), 48.0 (C3′), 69.4 (C1′), 69.7; 72.0 (d, Ce, ^1^ *J* C—P = 59.60 Hz), 73.9; 75.7; 75.8; 77.9 (d, Cg, ^3^ *J* C—P *=* 5.62 Hz), 76.0; 78.0; 78.2; 81.7 (d, Cf, ^2^ *J* C—P *=* 6.75; 22.50 Hz), 79.0 (C1″), 117.1 (C3), 120.9 (C9), 123.6 (C10), 128.1 (C4), 128.2 (C6), 129.0 (q, C12, ^1^ *J* C—F = 29.20 Hz), 129.5 (C7), 129.4; 129.7; 129.9; 130.3 (d, Cc, ^3^ *J* C—P *=* 11.50; 11.70; 11.00 Hz), 129.8 (C5), 130.4; 131.7 (d, Ca, ^1^ *J* C—P *=* 52.00; 75.00 Hz), 132.6; 132.8; 132.9; 133.7 (Cd), 133.5; 134.1; 136.0; 136.6 (d, Cb, ^2^ *J* C—P *=* 11.00; 13.34 Hz), 143.4 (C8), 148.4 (q, C11, ^1^ *J* C—F = 34.50 Hz), 153.6 (d, C2, ^2^ *J* C—F = 8.38 Hz). NMR-^31^P­{^1^H} (acetone-*d*_6_) [δ ppm, (multiplicity, assignation)]: 34.17 (d, dppf^b^); 29.98 (d, dppf^a^); ^2^ *J* P_a_-P_b_ = 28.50 Hz; −144.5 (PF_6_, sept, ^1^ *J* P—F = 711.24 Hz). ESI­(+)-MS (acetone:water): [M – PF_6_]^+^ (1037.1150 *m*/*z*, 6%), [MQ + H]^+^ (379.1250 *m*/*z*, 100%). Molar conductivity (DMSO): 29.36 ± 0.01 S cm^2^ mol^–1^.

##### [Pd­(bipy)­(MQ)]­PF_6_ (**13**)

A yellow solid was obtained with a yield of 82.4% (112 mg). Elemental analysis (%) Calc. for C_27_H_23_F_12_N_4_OPPd: C 41.32; H 2.95; N 7.14. Found: C 41.64; H 3.12; N 7.48. IR [*v*_max_ cm^–1^ (assignation)]: ν 3248 (N—H), ν 3059 (C—H aromatic), ν 2951 (C—H aliphatic), ν 1620 (C=C), ν 1597 and 1587 (C=N), 847 (PF_6_). NMR-^1^H (acetone-*d*_6_) [δ ppm, (integral; multiplicity; assignation, *J* Hz)]: 0.89; 2.58 (2H, d; m, H6′, ^3^ *J* = 13.30 Hz), 1.35; 1.79 (2H, m; d, H5′, ^3^ *J* = 14.16 Hz), 1.86; 1.88 (2H, m, H4′), 2.83; 3.70 (2H, m, H3′), 3.77 (1H, m, H1′), 6.16:6.17 (1H, s, H1″), 6.61 (1H, bs, NH), 7.85; 7.91 (2H, d; m, Hb, ^3^ *J*_*ortho*_ = 7.90 Hz), 7.92 (1H, m, H6), 8.36 (1H, s, H3), 8.27; 8.44 (2H, d; m, Hc, ^3^ *J*_*ortho*_ = 7.15 Hz), 8.43 (1H, m, H5), 8.54; 8.59 (2H, d, Hd, ^3^ *J*_*ortho*_ = 8.02; 7.98 Hz), 8.66; 8.69 (2H, d, Ha, ^3^ *J*_*ortho*_ = 8.58 Hz), 9.09 (1H, d, H7, ^3^ *J*_*ortho*_ = 4.7 Hz). NMR-^13^C­{^1^H} (acetone-*d*_6_) [δ ppm, (multiplicity; assignation)]: 23.3; 23.5 (C5′), 24.0; 24.1 (C6′), 25.1; 25.2 (C4′), 49.6; 49.8 (C3′), 71.1; 71.8 (C1′), 78.0; 78.1 (C1″), 116.8 (C3), 124.1; 124.8 (Cd), 124.4 (C9), 125.1 (C10), 126.1 (C4), 128.0 (C6), 128.4; 129.0 (Cb), 129.5; 151.1 (Ca), 130.1; 142.2 (Cc), 132.6 (C12), 142.1 (C5), 142.8 (C8), 149.0 (C7), 150.2 (C11), 152.1 (C2). NMR-^31^P­{^1^H} (acetone-*d*_6_) [δ ppm, (multiplicity, assignation, *J* Hz)]: −144.5 (PF_6_, sept, ^1^ *J* P—F = 707.32 Hz). ESI­(+)-MS-MS (acetone:water): [M – PF_6_]^+^ (639.0808 *m*/*z*, 100%), [M – PF_6_ – bipy]^+^ (483.0117 *m*/*z*, 30%. Molar conductivity (DMSO): 23.85 ± 0.01 S cm^2^ mol^–1^.

##### *cis*-[Pt­(PPh_3_)_2_(QN)­Cl]­PF_6_ (**14**)

A white solid was obtained with a yield of 91.2% (133 mg). IR [*v*_max_ cm^–1^ (assignation)]: ν 3320 (O—H), ν 3058 (C—H aromatic), ν 2970 (C—H aliphatic), ν 1615 (C=C), ν 1586 (C=N), ν 1096 (P—C), 864 (PF_6_), ν 548 (Pt—P), and ν 313 (Pt—Cl). UV–vis (DMSO) [λ (assignation)]: 261 nm (π–π* Ph); 331 nm (π–π* QN). NMR-^1^H (acetone-*d*_6_) [δ ppm, (integral; multiplicity; assignation, *J* Hz)]: 1.75; 2.32 (2H, m, H7), 2.12;2.27 (2H, m, H5), 2.38 (1H, m, H4), 3.05 (1H, m, H8), 3.63; 4.43 (2H, m, H2), 3.78; 3.95 (2H, m, H6), 4.01 (3H, s, H11′), 3.21; 5.29 (2H, d, H11, ^3^ *J* = 10.44; 17.40 Hz), 5.86 (1H, d, OH, ^3^ *J* = 3.48 Hz), 6.01 (1H, m, H10), 6.21 (1H, bs, H9), 7.25; 7.35 (12H, m, Hc), 7.41 (1H, d, H5′, ^4^ *J*_*meta*_ = 2.4 Hz), 7.44; 7.51 (6H, m, Hd), 7.63 (12H, m, Hb), 7.74 (1H, d, H3′, ^3^ *J*_*ortho*_ = 5.6 Hz), 7.88 (1H, dd, H7′, ^3^ *J*_*ortho*_ = 9.3 Hz, ^4^ *J*_*meta*_ = 2.4 Hz), 9.34 (1H, d, H2′, ^3^ *J*_*ortho*_ = 5.6 Hz), 9.36 (1H, d, H8′, ^3^ *J*_*ortho*_ = 9.3 Hz). NMR-^13^C­{^1^H} (acetone-*d*_6_) [δ ppm, (multiplicity; assignation, *J* Hz)]: 19.6 (C7), 24.7 (C5), 27.4 (C4), 37.4 (C8), 46.2 (C2), 55.6 (C6), 56.8 (C11′), 61.1 (C3), 68.2 (C9), 103.9 (C5′), 117.0 (C11), 122.1 (C3′), 124.1 (C7′), 129.3; 129.6 (d, Cc, ^3^ *J* C—P = 11.04; 11.80 Hz), 132.6; 132.9 (s, Cd), 135.0; 136.1 (d, Cb, ^2^ *J* C—P = 9.80; 10.60 Hz), 139.3 (C10), 141.2 (C10′), 149.2 (C9′), 151.2 (C4′), 151.4 (C2′), 160.4 (C6′). NMR-^31^P­{^1^H} (acetone-*d*_6_) [δ ppm, (multiplicity, assignation)]: 14.24 (d, ^b^PPh_3_-*trans*-Cl, ^1^ *J* P_b_-Pt = 3645.70 Hz); 4.11 (d, ^a^PPh_3_-*trans*-N, ^1^ *J* P_a_-Pt = 3306.68 Hz); ^2^ *J* P_a_-P_b_ = 17.60 Hz; −144.5 (PF_6_, sept, ^1^ *J* P—F = 706.12 Hz). Molar conductivity (DMSO): 22.30 ± 0.01 S cm^2^ mol^–1^.

### Parasites and Cell Culture

CQ-sensitive 3D7, NF54 and CQ-resistant W2 and K1 strains of *P. falciparum* were cultivated in human O^+^ erythrocytes (donated by HEMOBA, Salvador, Brazil) at 5% hematocrit with daily maintenance in RPMI-1640 medium (Sigma-Aldrich) supplemented with 5% (v/v) heat-inactivated human plasma (donated by HEMOBA, Salvador, Brazil), 25 mM 4-(2-hydroxyethyl)-1-piperazineethanesulfonic acid (HEPES, ChemCruz, Dallas, TX), 300 μM hypoxanthine (MP Biomedicals, Santa Ana, CA), 11 mM glucose (Sigma-Aldrich) and 10 μg/mL of gentamicin (Life, Carlsbad, CA). Five days prior to use, *P. falciparum* was cultivated without hypoxanthine and synchronized to rings by 5% D-sorbitol (USB, Santa Clara, CA). NK65 strain of *P. berghei* was routinely maintained in Swiss mice. J774 macrophages were cultured in Dulbecco’s modified Eagle’s medium (DMEM) (Sigma-Aldrich) supplemented with 10% (v/v) fetal bovine serum (FBS, Gibco, Gaithersburg, MD) and 50 μg/mL of gentamicin (Life). HepG2 cells were cultured in RPMI medium (Sigma-Aldrich) supplemented with 10% (v/v) fetal bovine serum (Gibco, Gaithersburg, MD) and 50 μg/mL of gentamicin (Life Technology).

### Animals for Malaria Infection and Pharmacokinetics

Male Swiss-Webster and C57BL/6 mice (18–22 g) were housed at Instituto Gonçalo Moniz (Fiocruz Bahia, Brazil), maintained in sterilized cages under a controlled environment, receiving a rodent balanced diet and water *ad libitum*. All experiments were carried out in accordance with the recommendations of Ethical Issues Guidelines in Brazil and were approved by the Animal Ethics Committee of Fiocruz Bahia (protocol numbers 002/2016 and 014/2018). All institutional and national guidelines for the care and use of laboratory animals were followed.

### Animals for Schistosome Experiments

*Ex vivo* schistosome experiments involving animals were conducted at the Rush University Medical Center (Chicago, USA) and were approved by the Institutional Animal Care and Use Committee of the Rush University Medical Center (Department of Health and Human Services animal welfare, assurance number A-3120-01) with protocol ID: 20-069. Three-week old, female Swiss-Webster mice obtained from the Charles River were housed in the Comparative Research Center of Rush University Medical Center (Chicago, IL, U.S.A.).

## Supplementary Material





## Notes

Molecular formula strings (CSV)
Spectra for the compounds, *in vitro* biological assays, *in vivo* efficacy studies assays, *in vitro* ADME assays, representative curve–concentration responses and raw data (PDF)

## Notes

CCDC 2475378, 2475380, and 2475379 contain the supplementary crystallographic data for complexes Pt (**2**), Pt (**6**), and Pd (**9**), respectively. These data can be obtained free of charge via the joint Cambridge Crystallographic Data Centre (CCDC) and Fachinformationszentrum Karlsruhe Access Structures service.

## ∇

Guangdong Technion-Israel Institute of Technology, Chemistry Program, Shantou, 515063, Guangdong Province, China

## Abbreviations

ABS: asexual blood stages
ATO: atovaquone
BHIA: β-hematin inhibitory activity
bipy: 2,2′-bipyridine
CQ: chloroquine
DHA: dihydroartemisinin
HepG2: hepatocellular carcinoma cells
Hz: hemozoin
ICP-MS: inductively coupled plasma-mass spectrometry
J774: murine macrophage lineage
MoA: mode of action
MQ: mefloquine
NTS: newly transformed schistosomula
*P. berguei*: *Plasmodium berguei*
*P. falciparum*: *Plasmodium falciparum*
pRBCs: parasitized red blood cells
PZQ: praziquantel
QN: quinine
SAR: structure–activity relationships
SD: standard deviation
S.I.: selectivity index
*S. mansoni*: *Schistosoma mansoni*
TEM: transmission electron microscopy
