# Supplementary material for: Examination of Coligands in Mefloquine–Metal Complexes Reveals the Structural Determinants of Activity against Plasmodium falciparum and Schistosoma mansoni
Source: J Med Chem. 2026 Mar 11;69(8):9242–64. doi: 10.1021/acs.jmedchem.5c03739 (PMC13126674; doi:10.1021/acs.jmedchem.5c03739)
Supplement: Supplementary file 2 [file jm5c03739_si_002.pdf]

Supporting Information for

Examination of co-ligands in mefloquine–metal complexes reveals the structural determinants of activity against *Plasmodium falciparum* and *Schistosoma mansoni*

Wilmer Villarreal,[a,b*,#] Helenita Costa Quadros,[c] Legna Colina-Vegas,[a,b,#] Sammy Y. Aboagye,[d] Godwin Akpeko Dziwornu,[e] Gabriel H. Ribeiro,[a] Ariane Isis Barros,[f] Dawid Jakub Kucharski,[g] Mahsa Rahbari,[h] Christina Brandstädter,[h] Sarah D'Alessandro,[i] Nicoletta Basilico,[j] Keabetswe Masike,[k] Nandi Mehlala,[k] Joaquim Araújo Nobrega,[a] Victor M. Deflon,[l] Maribel Navarro,[m] Przemysław J. Boratyński,[g] Kelly Chibale,[e,k] David L. Williams,[d] Alzir A. Batista,[a,*] Diogo R. M. Moreira [c,*]

[a] Universidade Federal de São Carlos, Departamento de Química, São Carlos, 13565-905, SP, Brazil.

[b] Universidade Federal do Rio Grande do Sul, Instituto de Química, Porto Alegre, 91501-970, RS, Brazil.

[c] Fundação Oswaldo Cruz, Instituto Gonçalo Moniz, Salvador, 40296-710, BA, Brazil.

[d] Rush University Medical Center, Department of Microbial Pathogens and Immunity, Chicago, Illinois 60612, United States of America.

[e] Drug Discovery and Development Centre (H3D), Department of Chemistry, University of Cape Town, Rondebosch 7701, South Africa.

[f] Universidade Federal de Mato Grosso, Departamento de Solos e Engenharia Rural, Cuiabá, 78060-900, MT, Brazil.

[g] Department of Organic and Medicinal Chemistry, Wrocław University of Technology, Wyb. Wyspiańskiego 26, Wrocław 50-370, Poland.

[h] Biochemistry and Molecular Biology, Interdisciplinary Research Center, Justus Liebig University Giessen, Heinrich-Buff-Ring 26-32, Giessen, 35392, Germany.

[i] Dipartimento di Scienze Farmacologiche e Biomolecolari, Università degli Studi di Milano, Milan, 20133, Italy.

[j] Dipartimento di Scienze Biomediche, Chirurgiche e Odontoiatriche, Universitá degli Studi di Milano, Milan, 20133, Italy.

[k] South African Medical Research Council Drug Discovery and Development Research Unit, Department of Chemistry and Institute of Infectious Diseases and Molecular Medicine, University of Cape Town, Rondebosch 7701, South Africa.

[l] Universidade de São Paulo, Instituto de Química de São Carlos, São Carlos, 13560-970, SP, Brazil.

[m] Universidade Federal de Juiz de Fora, Departamento de Química, Juiz de Fora, 36036-900, MG, Brazil.

[#]Current address: Guangdong Technion-Israel Institute of Technology, Chemistry Program, Shantou, 515063, Guangdong Province, China.

*Corresponding authors: Wilmer Villarreal at wilmer.villarreal@gtiit.edu.cn; Alzir A. Batista at daab@ufscar.br, Diogo R. M. Moreira at diogo.magalhaes@fiocruz.br

TABLE OF CONTENT

Entry	Caption	Page number
**Scheme 1**	Numbering of atoms used in assigning the NMR data of the MQ ligand.	S6
**Scheme 2**	Numbering of atoms used in assigning the NMR data of the QN ligand.	S7
**Figure S1**	^{1}H and COSY ^{1}H-^{1}H NMR spectra for the complex Pt (**1**) in acetone-*d*$_6$.	S8
**Figure S2**	$^{13}C\{^{1}H\}$, HSQC ^{13}C-^{1}H and HMBC ^{13}C-^{1}H NMR spectra for the complex Pt (**1**) in acetone-*d*$_6$.	S9
**Table S1**	Chemical shift of hydrogens atoms in the ^{1}H NMR (in acetone-*d*$_6$) for the MQ free ligand and the MQ in the complexes (**1-13).**	S10
**Figure S3**	$^{31}P\{^{1}H\}$ NMR spectra for the complexes (**1-14**) in acetone-*d*$_6$.	S11
**Figure S4**	MS spectra of complex Pt (**7**) (panel A), MS-MS spectra (panel B), experimental isotopic relation (panel C) and theory isotopic relation (panel D).	S12
**Table S2**	Selected interatomic distances (Å) and angles (deg) for the complexes Pt (**2**), Pt (**6**) and Pd (**9**).	S13
**Figure S5**	$^{31}P\{^{1}H\}$ NMR spectra of metal complexes Pt (**3**) and Pd (**9**) in DMSO-d$_6$ and in a DMSO-*d*$_6$ /D_2O mixture (70:30 v/v) at 298K and recorded in different times (fresh solutions, 24h, 48h and 72h). Panel (A): complex Pt (**3**) in DMSO-*d*$_6$. Panel (B): complex Pt (**3**) in DMSO-d$_6$ /D_2O mixture (70:30 v/v). Panel (C): complex Pd (**9**) in DMSO-*d*$_6$. Panel (D): complex Pd (**9**) in DMSO-*d*$_6$ /D_2O mixture (70:30 v/v).	S14
**Figure S6**	$^{31}P\{^{1}H\}$ NMR spectra of metal complexes Pt (**3**), Pd (**9**), Pt (**14**) e Pt (**15**) in a mixture of DMSO-d$_6$:cell culture at (70:30 v/v) at 298K and recorded in different times (fresh solutions, 24h, 48h and 72h). Panel (A): complex Pt (**3**). Panel (B): complex Pd (**9**). Panel (C): complex Pt (**15**). Panel (D): complex Pt (**14**).	S15
**Figure S7**	$^{31}P\{^{1}H\}$ NMR spectra of metal complex Pt (**3**) in a DMSO-*d*$_6$ /D_2O mixture (70:30 v/v) at 298K in the presence of GSH (panel A) or GSSG (panel B) recorded in different times (fresh solutions, 1h, 2h, 6h, 12h, and 24h).	S16
**Figure S8**	$^{31}P\{^{1}H\}$ NMR spectra of metal complexes Pd (**9**) in a DMSO-*d*$_6$ /D_2O mixture (70:30 v/v) at 298K GSH (upper) or GSSG (lower) recorded in different times (fresh solutions, 1h, 2h, 6h, 12h, and 24h).	S18
**Figure S9**	Panels A,B) Representative readings of the [^{3}H]-hypoxanthine incorporation in W2 strain of *P. falciparum* under treatment. Incubation of drugs for 72 h. Panel C) Curve-concentration response of parasite growth. Values normalized for untreated control. Bars are the mean and error bars are S.D. of one experiment using triplicates. ***p*<0.01; ****p*<0.005 by unpaired and nonparametric Mann–Whitney rank test.	S19
**Figure S10**	Representative curve-concentration response of cell viability normalized for untreated control. Experiment performed in J774 cell lineage and using AlamarBlue readout after 72 h of drug exposure. Dots are the mean and error bars are S.D. of one experiment using triplicates. MQ = mefloquine; CQ = chloroquine.	S20
**Table S3**	Compound solubility in phosphate buffered saline (PBS) in pH of 7.4 at 37 °C for 1 h and GSH binding in the same conditions and recorded at 1 h or 24 h.	S21
**Table S4**	Association constant (log *K*) for hemin, hypochromism of Soret's hemin band (Δε, %) and β-hematin inhibitory activity (BHIA) for the mefloquine-metal complexes.	S22

**Table S5**	Cytotoxicity in mammal cells of the frontrunner metal complexes with MQ, Pt (**3**) and Pd (**9**).	S23
**Table S6**	Examination of the activity for mefloquine–metal conjugates across a panel of different strains and their speed of inhibitory activity on *P. falciparum* growth.	S24
**Table S7**	Inhibitory effects on the enzymatic activity of recombinant flavoproteins *P. falciparum* thioredoxin reductase (*Pf*TrXR); human thioredoxin reductase (*h*TrxR1) and *S. mansoni* thioredoxin-glutathione reductase (*Sm*TGR).	S25
**Table S8**	Suppressive Peters test (treatment initiated 3 h post-infection) on parasitemia and animal survival in NK65 strain of *P. berghei*-infected Swiss mice (male).	S26
**Table S9**	Curative Thompson test (treatment initiated on 3rd post-infection) on parasitemia and animal survival in NK65 strain of *P. berghei*-infected Swiss mice (male).	S27
**Table S10**	Cumulative chemical transformation (%) of metal complexes (inferred by ^{31}P {^{1}H} NMR spectra and further confirmed by ^{1}H NMR and ^{13}C{^{1}H} NMR) and for Mefloquine (by ^{1}H NMR).	S28
**Figure S11**	Panel A) ^{1}H NMR spectra in deshielding region of Mefloquine (MQ) in DMSO-d_6/RPMI culture medium mixture (70:30) in different times (20 min., 2h, 24h, 48h and 72h) at 298K. Panel B) ^{1}H NMR spectra in shielding region of Mefloquine (MQ) in DMSO-d_6/RPMI culture medium mixture (70:30) in different times (20 min., 2h, 24h, 48h and 72h) at 298K.	S29
**Figure S12**	Panel A) ^{1}H NMR spectra in the deshielding region of complex [Pt(dppe)MQ]PF_6 Pt (**3**) in DMSO-d_6/RPMI culture medium mixture (70:30) in different times (20 min., 2h, 24h, 48h and 72h) at 298K. Panel B) ^{1}H NMR spectra in the shielding region of complex [Pt(dppe)MQ]PF_6 Pt (**3**) in DMSO-d_6/RPMI culture medium mixture (70:30) in different times (20 min., 2h, 24h, 48h and 72h) at 298K.	S30
**Figure S13**	Panel A) ^{1}H NMR spectra in the deshielding region of complex [Pd(dppe)MQ]PF_6 Pd (**9**) in DMSO-d_6/RPMI culture media mixture (70:30) in different times (20 min., 2h, 24h, 48h and 72h) at 298K. Panel B) ^{1}H NMR spectra in the shielding region of complex [Pd(dppe)MQ]PF_6 Pd (**9**) in DMSO-d_6/RPMI culture media mixture (70:30) in different times (20 min., 2h, 24h, 48h and 72h) at 298K.	S31
--	COMPLEMENTARY DESCRIPTION OF RESULTS	S32
1.0	1.0 Cell-free assays	S36
**1.1**	1.1 Determination of aqueous solubility	S36
**1.2**	Determination of aqueous stability by NMR	S36
**1.3**	Stability in the presence of reduced glutathione (GSH) by fluorescence assay	S36
**1.4**	Stability in the presence of GSH and GSSG by NMR analysis	S37
**1.5**	Interactions with ferriprotoporphyrin [$Fe^{(III)}$PPIX]	S38
**1.6**	1.6 Inhibition of β-hematin formation by UV-vis spectroscopy	S38
**2.0**	2.0 Pharmacology	S38
**2.1**	Drugs and dilutions	S38
**2.2**	Parasites and cell culture	S38
**2.3**	2.3 Cell cytotoxicity	S39
**2.4**	2.4 Hemolysis assay	S40
**2.5**	2.5 Screening of inhibitory activity for *P. falciparum* ring stages	S40
**2.6**	2.6 Antiparasitic activity for MQ-resistant strain of *P. falciparum*	S41
**2.7**	2.7 Parasite recrudescence assay	S41
**2.8**	2.8 Antiparasitic activity for specific parasite stages of *P. falciparum*	S42
**2.9**	2.9 Antiparasitic activity for *P. falciparum* gametocyte stages	S42

**2.10**	2.10 Speed of antiparasitic activity against *P. falciparum*	S43
**2.11**	2.11 Quantification of reactive oxygen species in trophozoite stages of *P. falciparum*	S43
**2.12**	2.12 Effects of MQ on *P. falciparum* redox homeostasis	S44
**2.13**	2.13 Parasite harvesting for microscopy and spectroscopy	S45
**2.14**	2.14 Transmission electron microscopy (TEM)	S45
**2.15**	2.15 Animals for malaria infection and pharmacokinetics	S45
**2.16**	2.16 Flow cytometry determination of *in vivo* parasitemia	S46
**2.17**	2.17 *In vivo* activity (Peters test)	S46
**2.18**	*In vivo* activity (Thompson test)	S47
**2.19**	2.19 Quantification of hemozoin in the blood of *P. berghei*-infected mice	S47
**2.20**	Parasite harvesting for ICP-MS, TEM and EDXS	S48
**2.21**	Energy-Dispersive X-ray (EDXS) spectroscopy	S48
**2.22**	2.22 Pharmacokinetics	S49
**2.23**	2.23 Quantification of platinum by ICP-MS analysis	S49
**2.24**	2.24 Enzyme preparation and activity determination	S50
**2.25**	2.25 Evaluation of schistosomicidal activity	S51
**2.26**	2.26 Preparation of newly transformed schistosomula (NTS)	S51
**2.27**	2.27 Preparation of adult *S. mansoni* worms	S51
**2.28**	2.28 Schistosomicidal activity of compounds against NTS and adult worms	S52
**2.29**	2.29 TRFS-green fluorescence quantification for TGR inhibition in worms	S52
**2.30**	2.30 *In vitro* metabolic stability assay in microsomes	S53
**2.31**	Statistical analyses	S53
**3.0**	References	S54

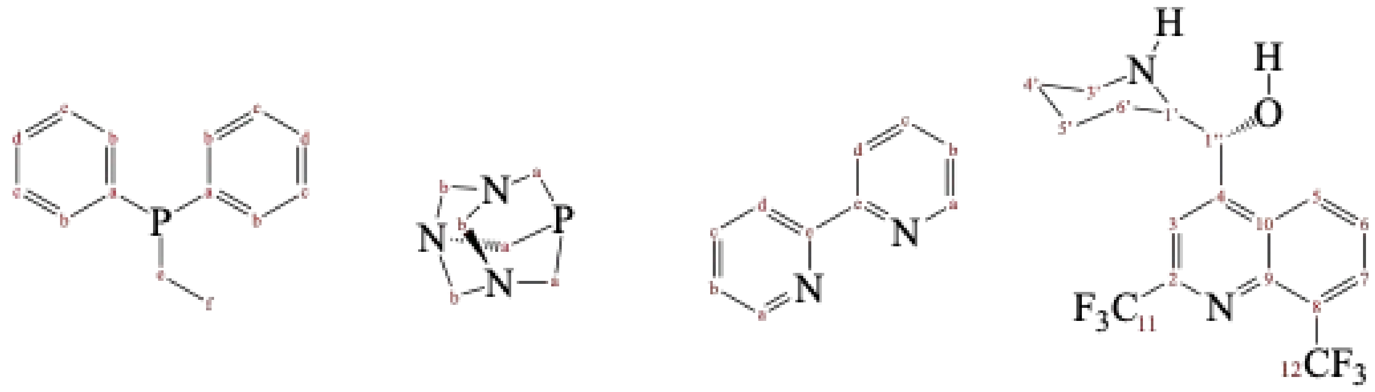

Phosphine PTA 2,2′-bipyridine Mefloquine

Scheme 1: Numbering of atoms used in assigning the NMR data of the MQ ligand.

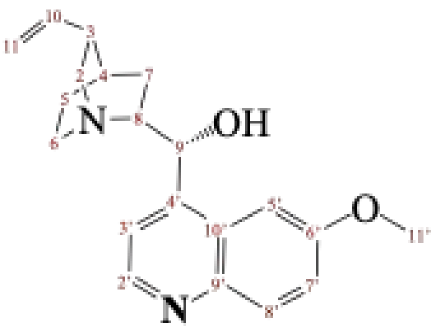

Scheme 2: Numbering of atoms used in assigning the NMR data of the QN ligand.

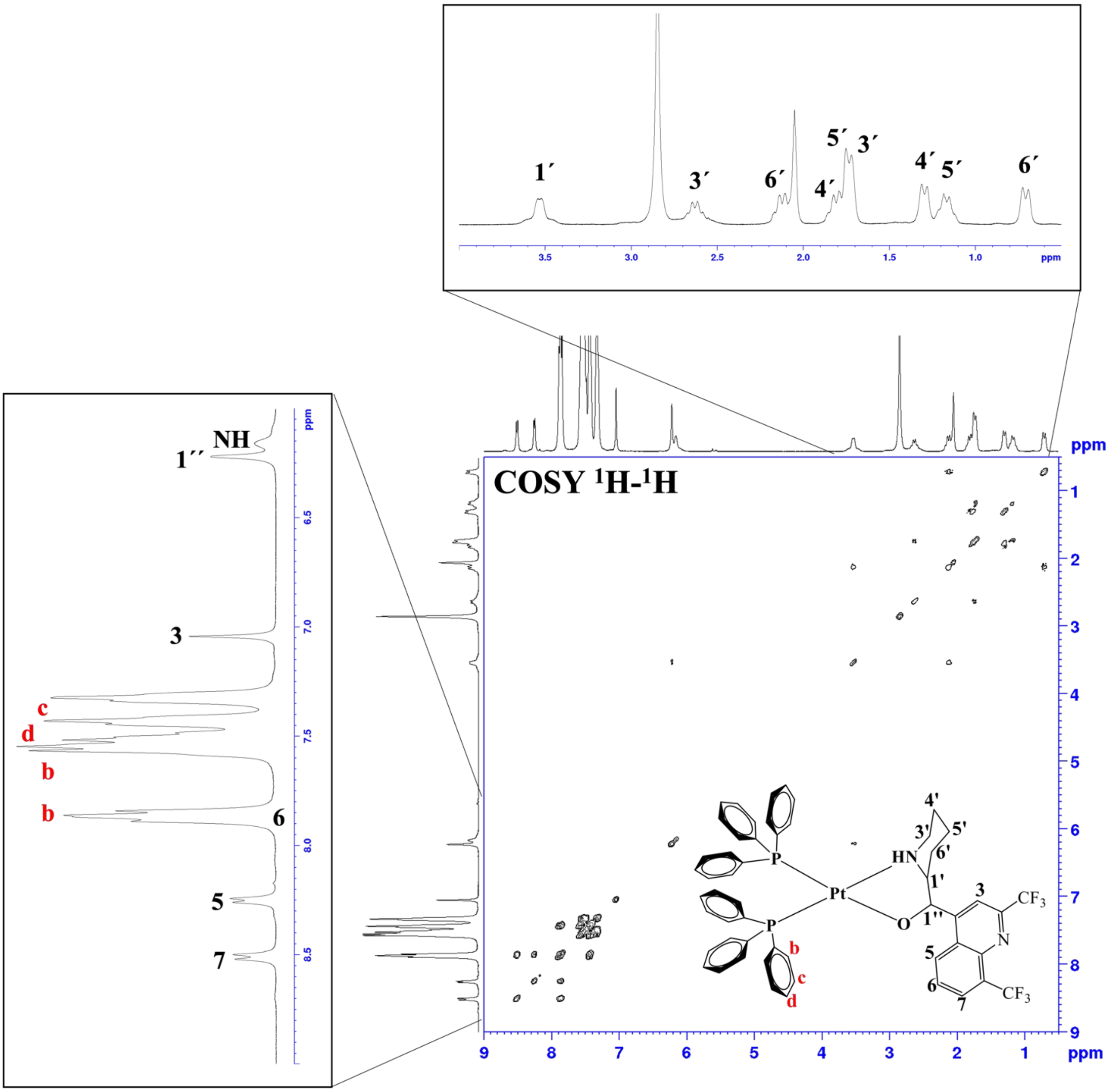

Figure S1. ^{1}H and COSY ^{1}H-^{1}H NMR spectra for the complex Pt (**1**) in acetone-d_6.

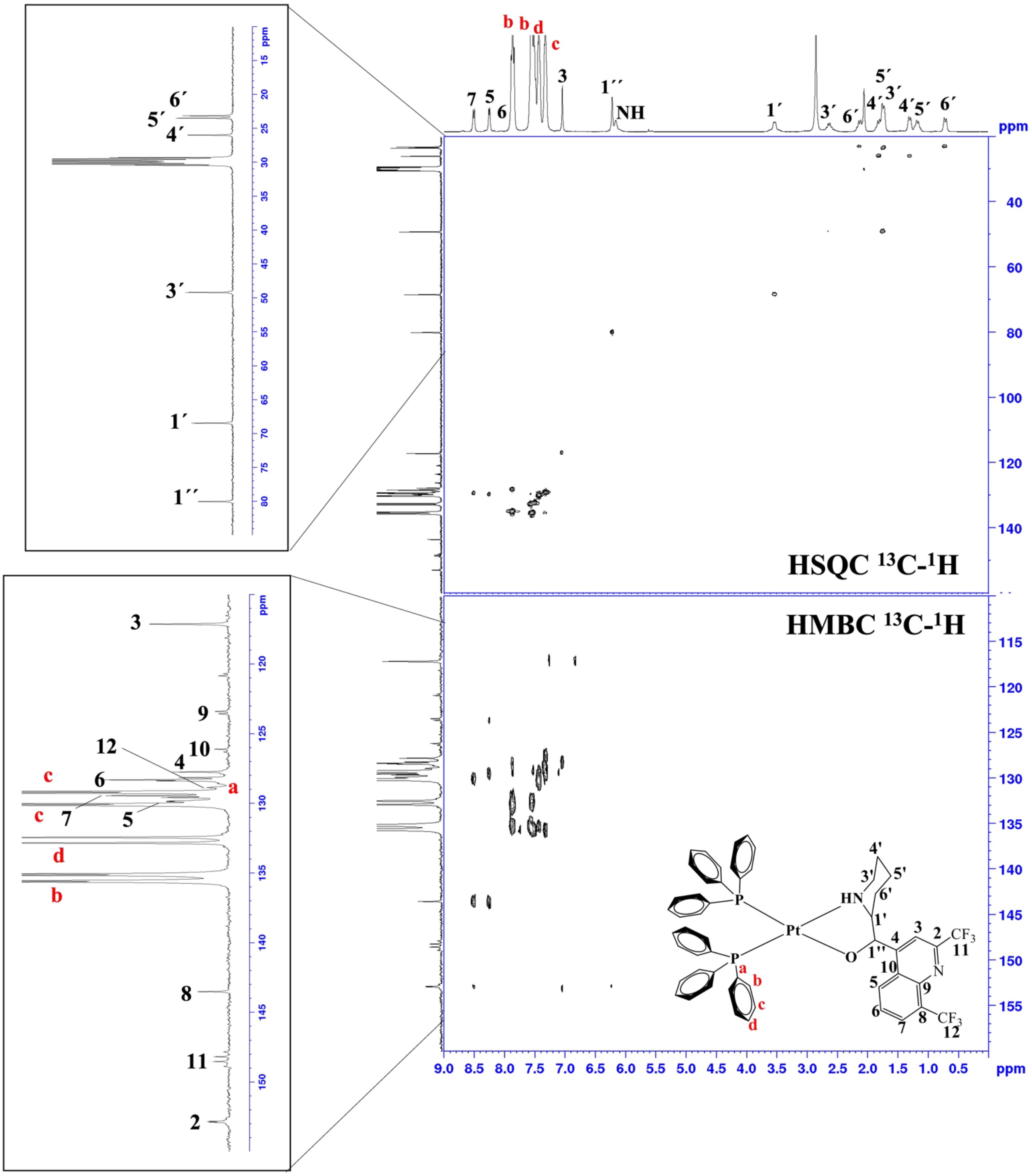

Figure S2. $^{13}C\{^1H\}$, HSQC ^{13}C-^1H and HMBC ^{13}C-^1H NMR spectra for the complex Pt (**1**) in acetone-d_6.

Table S1. Chemical shift of hydrogens atoms in the ^{1}H NMR (in acetone-d_6) for the MQ free ligand and the MQ in the complexes (**1-13).**

	H7	H3	H5	H6	NH	H1''	H1'	H3'	OH	H4'	H5'	H6'
**MQ**	8.50	8.26	7.82	7.01	6.87	6.45	3.56	3.22;3.83	2.93	1.89;2.01	1.37;1.76	1.24;1.93
**1**	8.51	7.04	8.25	7.86	6.16	6.22	3.53	1.72;2.63	-	1.30;1.81	1.17;1.75	0.71;2.12
**2**	8.56	8.51	8.33	7.94	6.66	6.21	3.56	3.20;3.59	-	1.10;1.41	0.89;0.95	0.76;2.01
**3**	8.68:8.82	8.18:8.21	8.06:8.30	7.52:7.88	*Overlap*	5.59:6.53	2.97:3.50	2.56;2.98:2.91;3.34	-	1.00;1.37:1.29	0.78;0.85:1.38	0.66;1.83:1.21;1.72
**4**	8.43	7.45	8.25	7.86	6.10	5.99	3.50	1.95;2.73	-	1.04;1.51	1.07;1.58	0.66;1.99
**5**	8.46	7.54	8.26	7.86	5.85	6.03	3.46	1.79;2.64	-	1.17;1.45	1.08;1.58	0.68;1.95
**6**	8.47	7.03	8.26	7.86	6.13	6.07	3.49	1.45;2.51	-	1.24;1.78	1.17;1.75	0.71;2.22
**7**	8.33	8.09	8.29	7.89	6.96	5.57	3.38	3.29	-	1.42;1.75	1.12;1.55	0.52;1.77
**8**	8.59:8.66	8.26:8.50	8.31:8.37	7.92:7.98	6.23	6.24:6.27	3.55:4.09	2.97;3.06:3.46;3.81	-	1.52;1.91	0.97:1.06;1.46	0.79;2.11:1.86
**9**	8.58	8.25	8.32	7.89	6.12	6.22	3.58	2.53;2.83	-	1.05;1.51	1.00	0.78;1.95
**10**	8.49	7.39	8.24	7.84	5.75	6.07	3.46	1.65;2.36	-	1.01;1.32	1.04;1.57	0.67;1.97
**11**	8.50	7.61	8.25	7.85	5.56	6.04	3.46	1.33;2.23	-	1.03;1.58	1.17;1.22	0.71;1.94
**12**	8.50	7.05	8.25	7.85	5.75	6.11	3.53	1.21;2.16	-	1.25;1.58	1.12;1.76	0.74;2.24
**13**	9.09	8.36	8.43	7.92	6.61	6.16:6.17	3.77	2.83;3.70	-	1.86;1.88	1.35;1.79	0.89;2.58

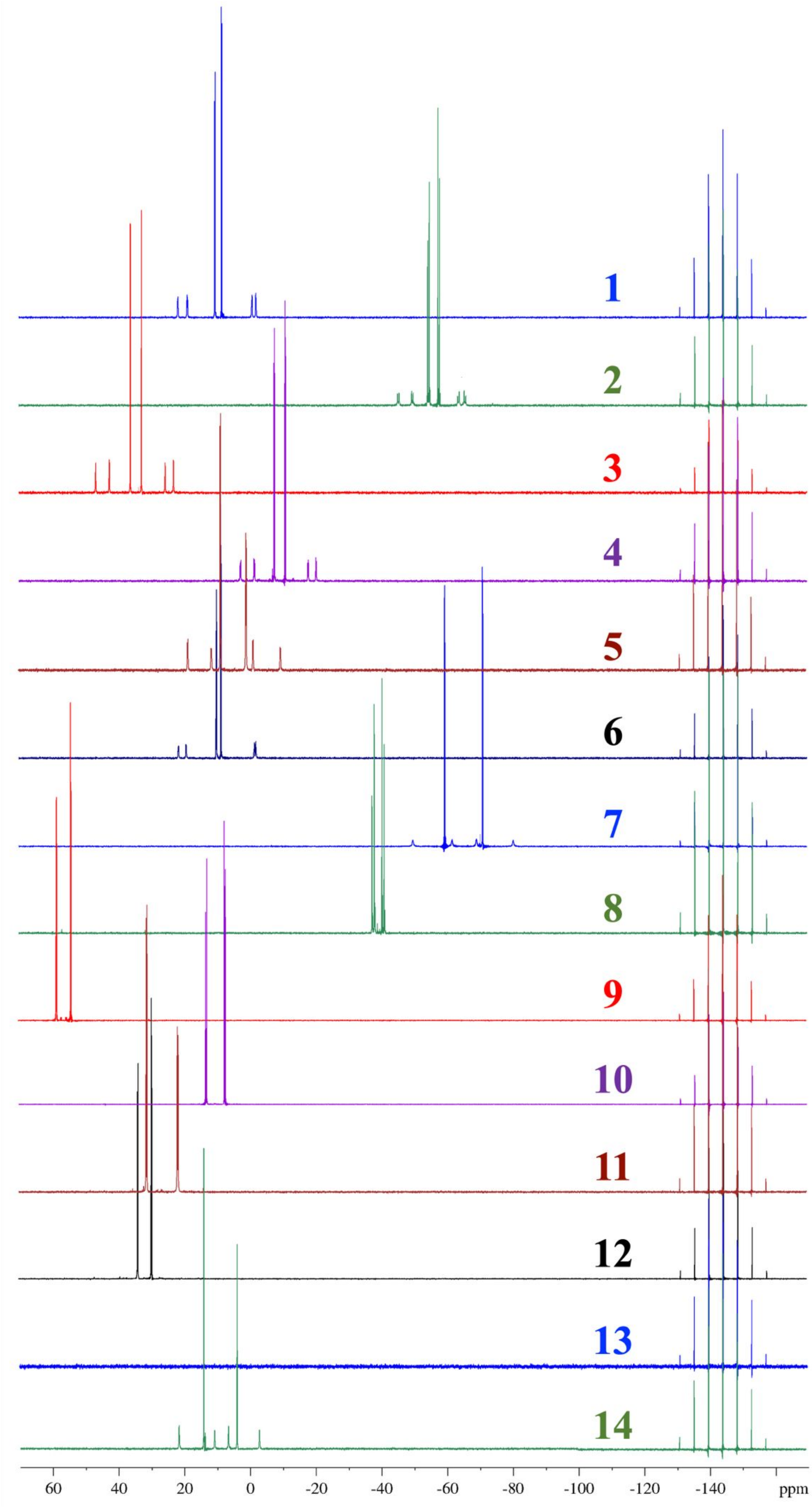
1
2
3
4
5
6
7
8
9
10
11
12
13
14
60
40
20
0
-20
-40
-60
-80
-100
-120
-140
ppm

Figure S3. $^{31}P\{^{1}H\}$ NMR spectra for the complexes (**1-14**) in acetone-d_6.

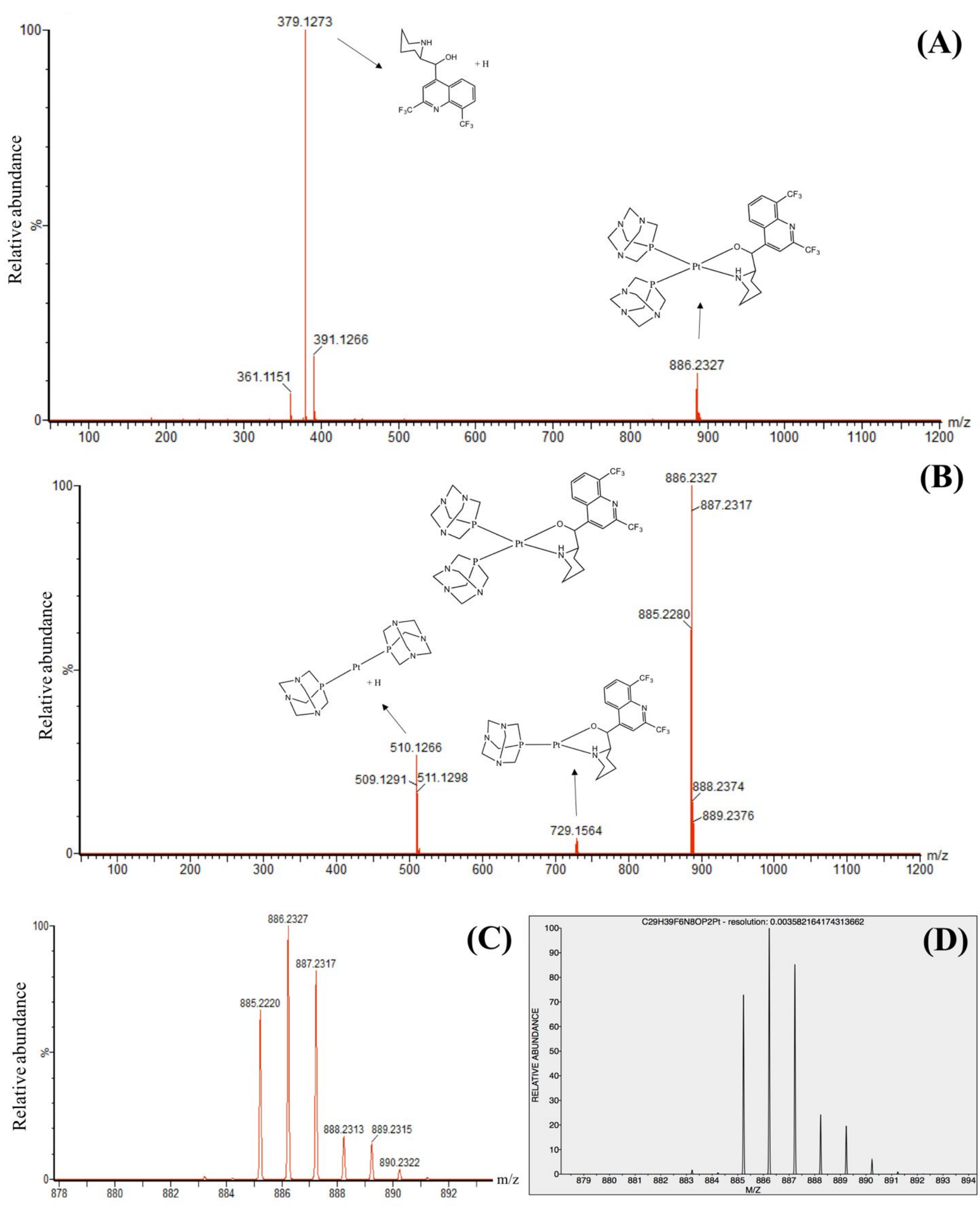

Figure S4. MS spectra of complex Pt (**7**) (panel A), MS-MS spectra (panel B), experimental isotopic relation (panel C) and theory isotopic relation (panel D).

Table S2. Selected interatomic distances (Å) and angles (deg) for the complexes Pt (**2**), Pt (**6**) and Pd (**9**).

**Bond**	Pt (**2**)	Pt (**6**)	Pd (**9**)
M – P1	2.239	2.271	2.248
M – P2	2.236	2.253	2.259
M – O	2.019	2.031	2.005
M – N	2.130	2.141	2.138
P1 – M – P2	73.55	96.50	84.80
P1 – M – N	179.04	163.21	172.14
P1 – M – O	98.96	82.47	89.93
P2 – M – N	105.92	99.74	102.75
P2 – M – O	172.42	173.58	174.63
N – M – O	81.66	81.86	82.55

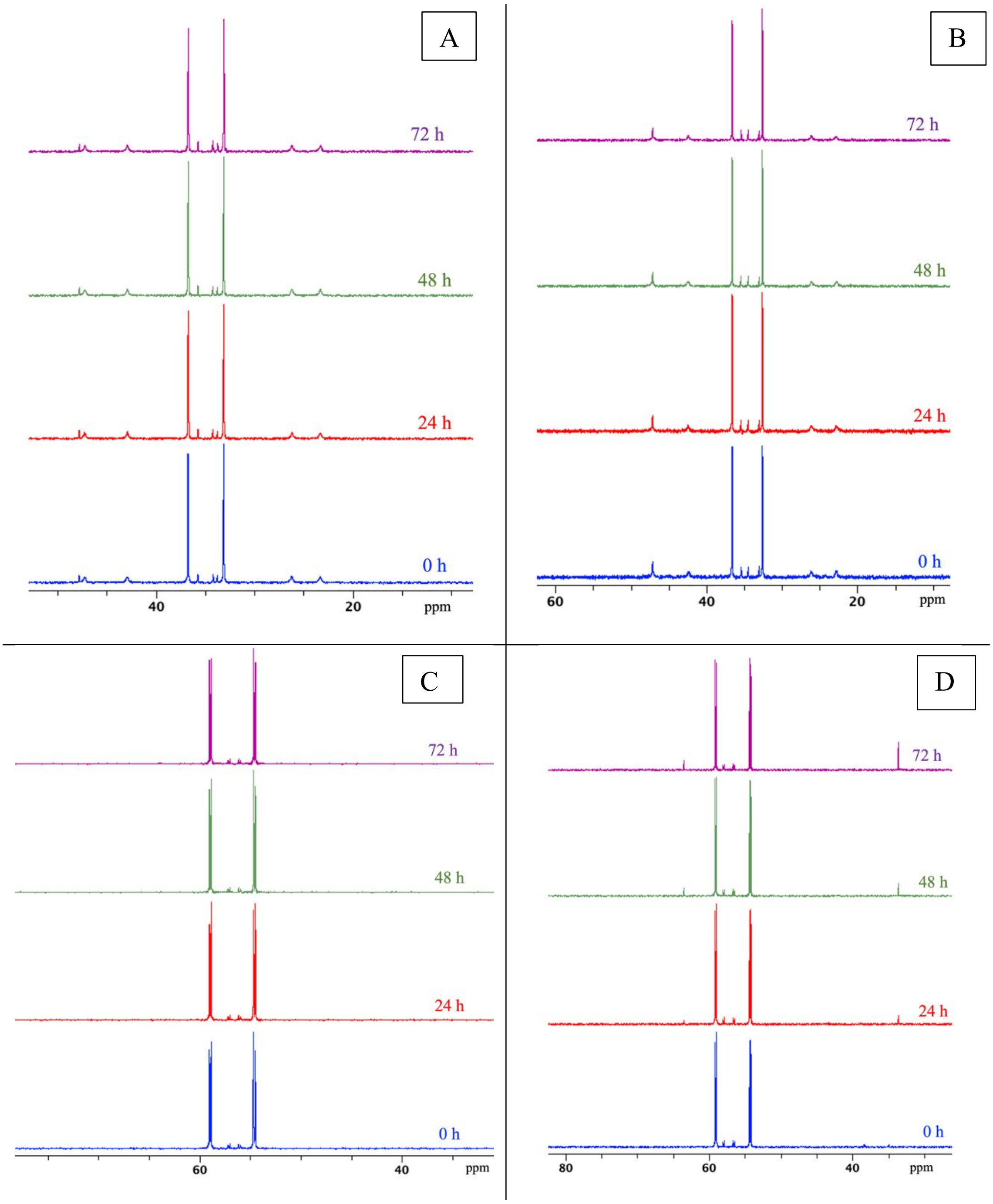

Figure S5: $^{31}P\ \{^1H\}$ NMR spectra of metal complexes Pt (**3**) and Pd (**9**) in DMSO-d$_6$ and in a DMSO-d_6 /D_2O mixture (70:30 v/v) at 298K and recorded in different times (fresh solutions, 24h, 48h and 72h). Panel (A): complex Pt (**3**) in DMSO-d_6. Panel (B): complex Pt (**3**) in DMSO-d$_6$ /D_2O mixture (70:30 v/v). Panel (C): complex Pd (**9**) in DMSO-d_6. Panel (D): complex Pd (**9**) in DMSO-d_6 /D_2O mixture (70:30 v/v).

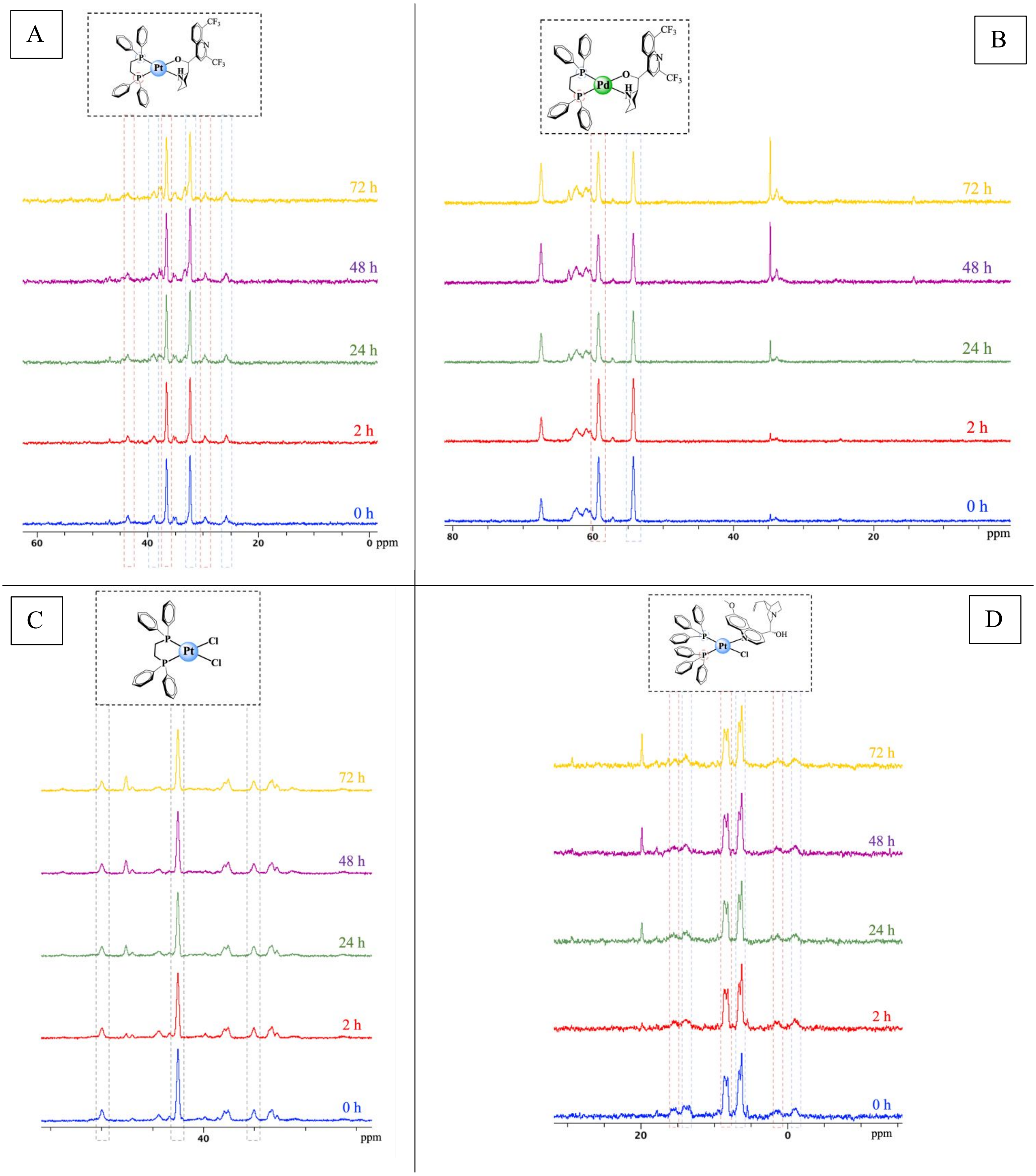

Figure S6: ^{31}P {^{1}H} NMR spectra of metal complexes Pt (**3**), Pd (**9**), Pt (**14**) and Pt (**15**) in a mixture of DMSO-d_6:cell culture at (70:30 v/v) at 298K and recorded in different times (fresh solutions, 24h, 48h and 72h). Panel (A): complex Pt (**3**). Panel (B): complex Pd (**9**). Panel (C): complex Pt (**15**). Panel (D): complex Pt (**14**).

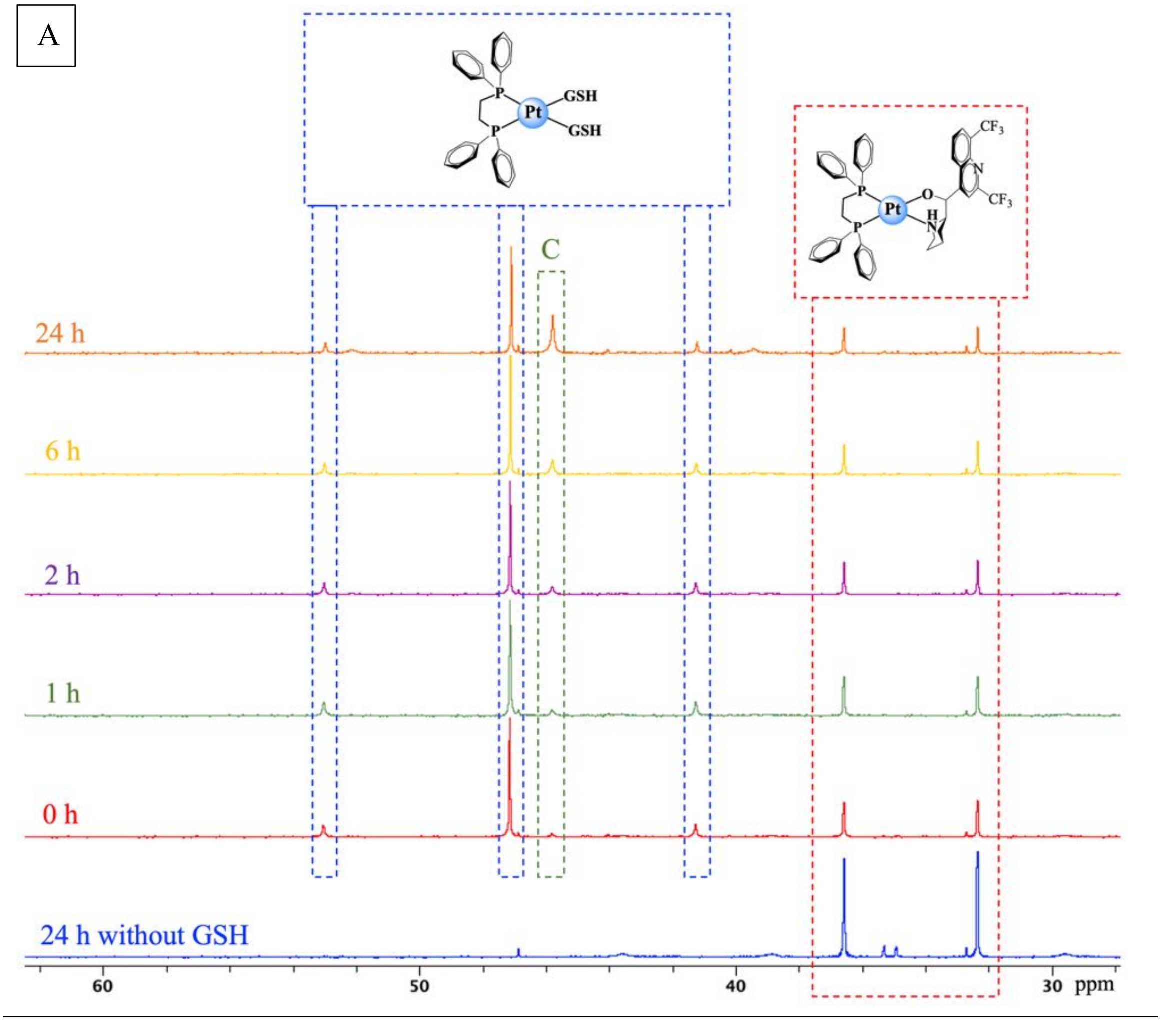

A
GSH
GSH
Pt
P
C
CF3
N
O
H
24 h
6 h
2 h
1 h
0 h
24 h without GSH
60
50
40
30
ppm

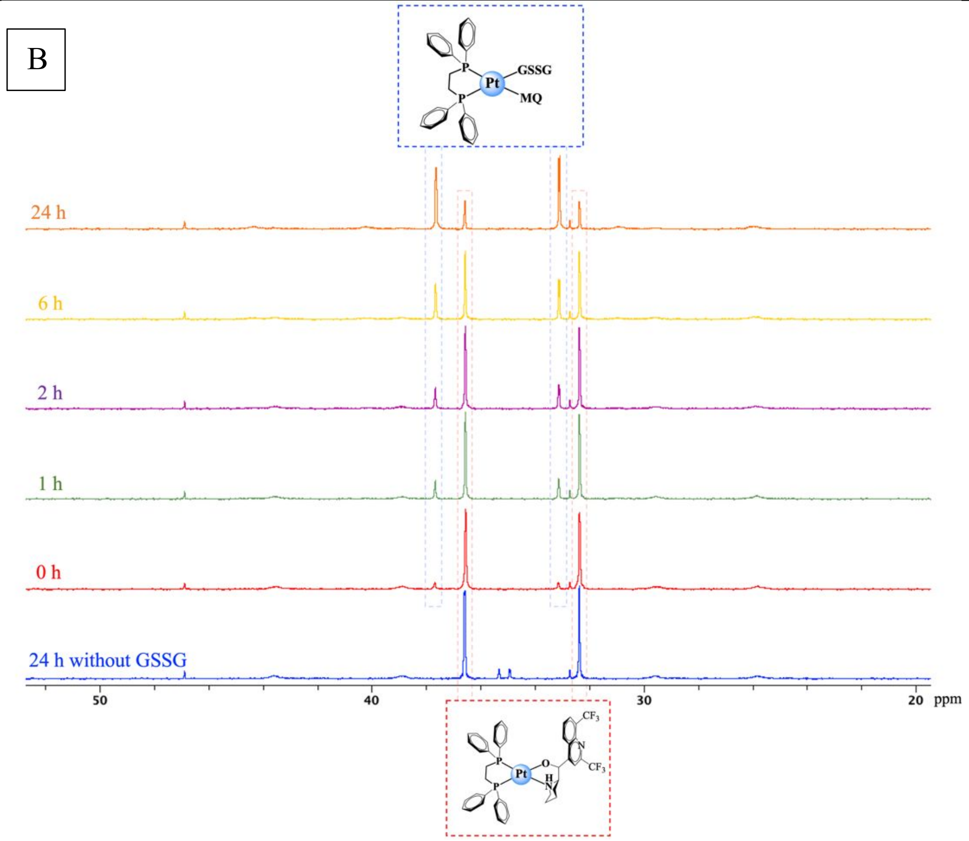

Figure S7: $^{31}P\{^{1}H\}$ NMR spectra of metal complex Pt (**3**) in a DMSO-d_6 /D_2O mixture (70:30 v/v) at 298K in the presence of GSH (panel A) or GSSG (panel B) recorded in different times (fresh solutions, 1h, 2h, 6h, 12h, and 24h).

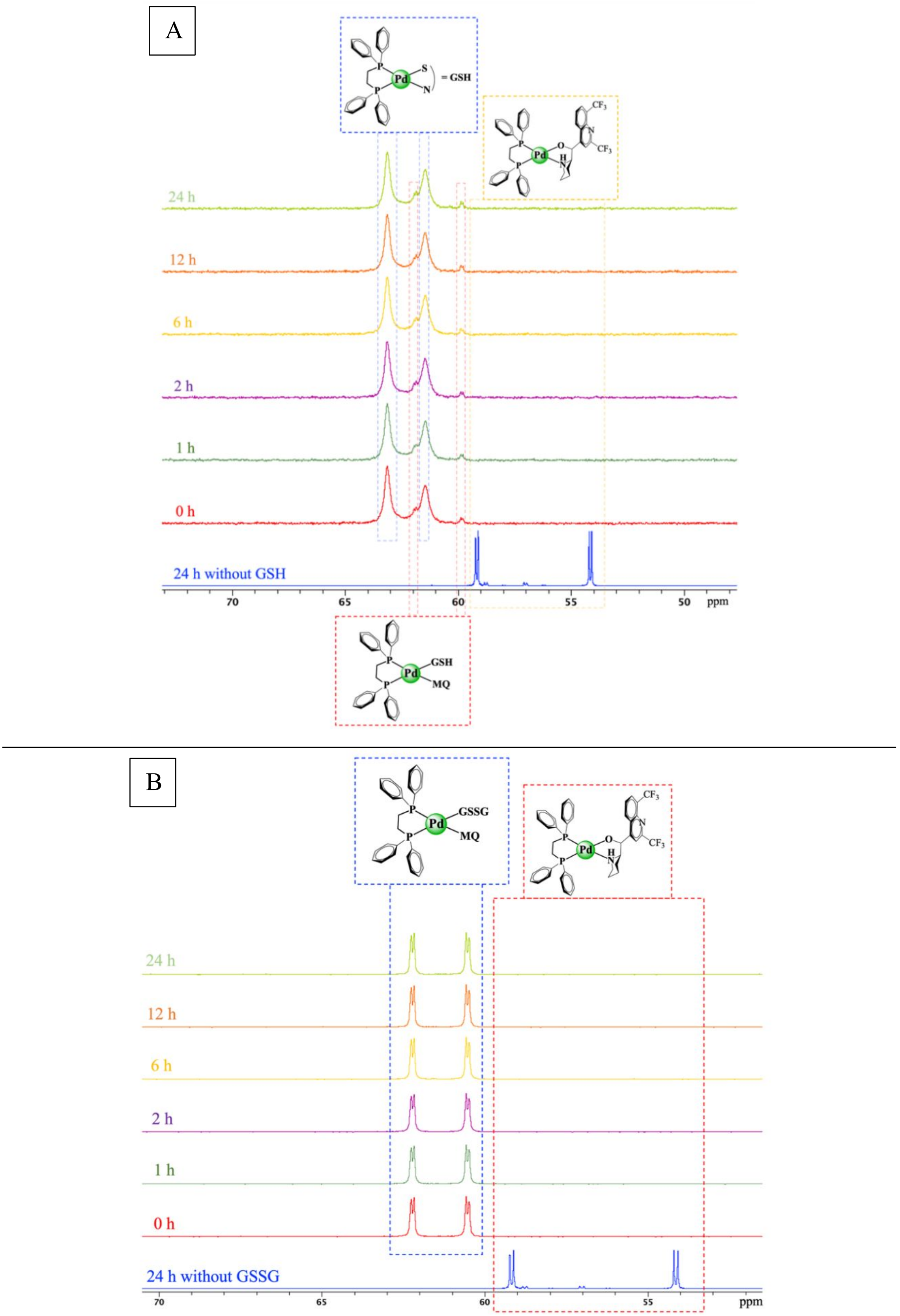

Figure S8: $^{31}P\{^{1}H\}$ NMR spectra of metal complexes Pd (**9**) in a DMSO-d_6/D_2O mixture (70:30 v/v) at 298K GSH (panel A) or GSSG (panel B) recorded in different times (fresh solutions, 1h, 2h, 6h, 12h, and 24h).

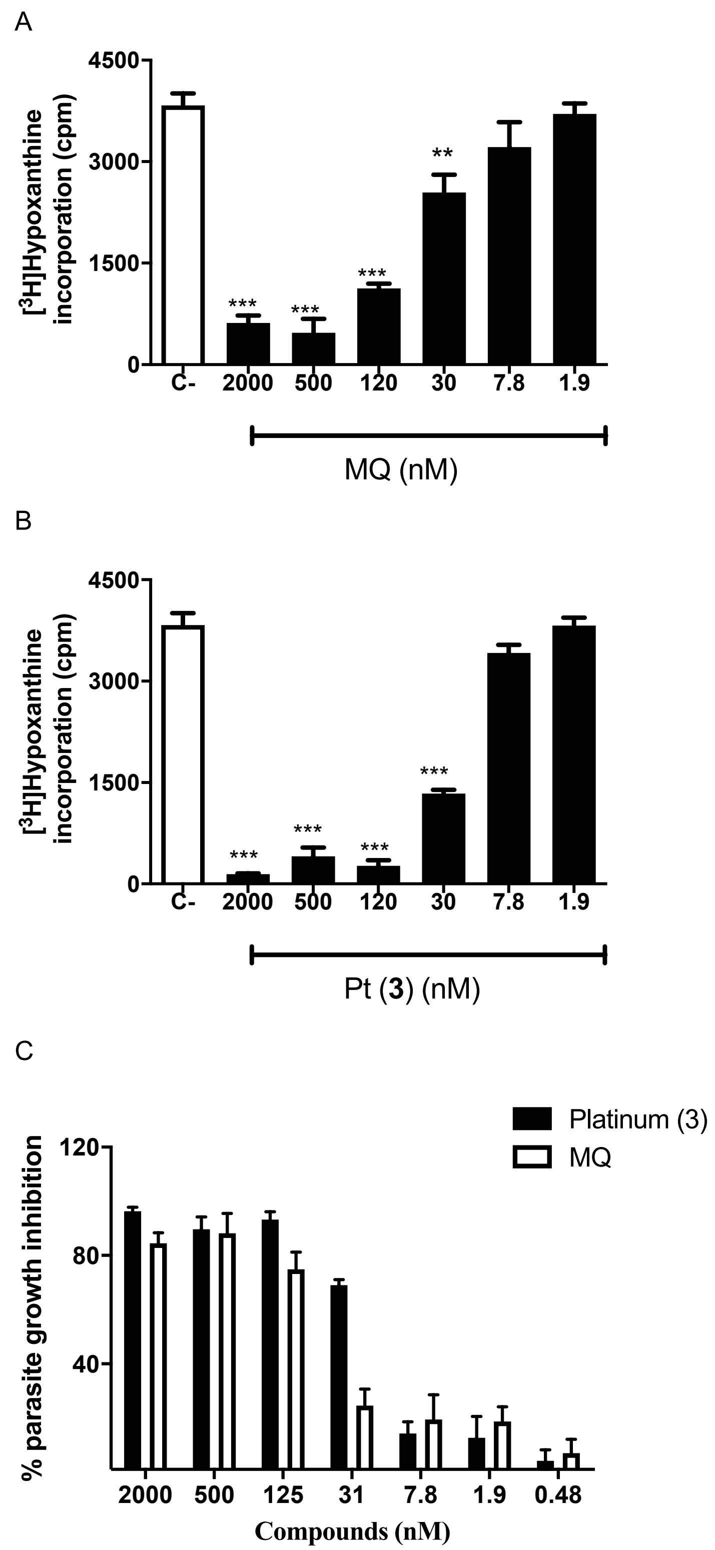

Figure S9: Panels A,B) Representative readings of the [^{3}H]-hypoxanthine incorporation in W2 strain of *P. falciparum* under treatment. Incubation of drugs for 72 h. Panel C) Curve-concentration response of parasite growth. Values normalized for untreated control. Bars are the mean and error bars are S.D. of one experiment using triplicates. ***p*<0.01; ****p*<0.005 by unpaired and nonparametric Mann–Whitney rank test.

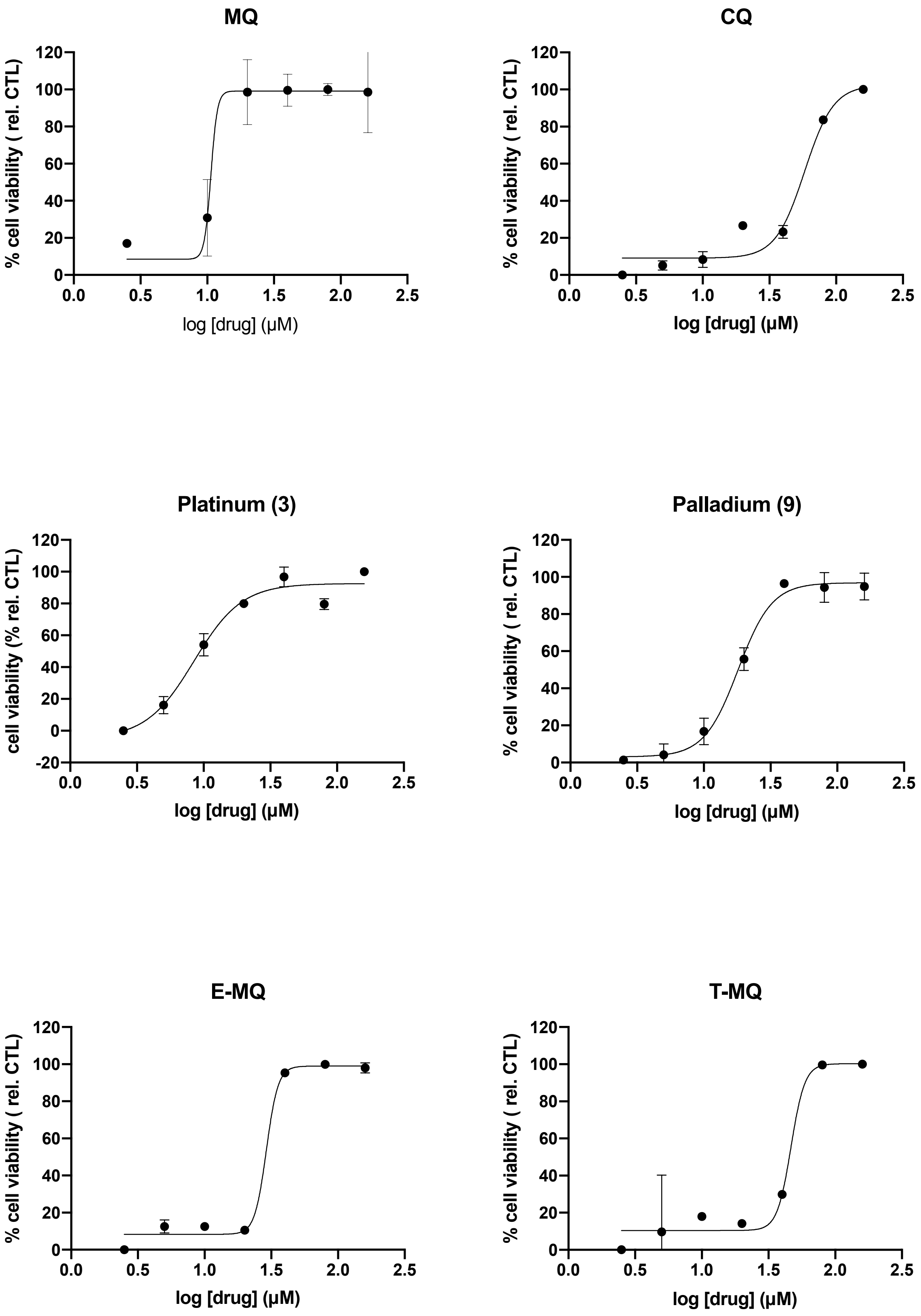

Figure S10: Representative curve-concentration response of cell viability normalized for untreated control. Experiment performed in J774 cell lineage and using AlamarBlue readout after 72 h of drug exposure. Dots are the mean and error bars are S.D. of one experiment using triplicates. MQ = mefloquine; CQ = chloroquine.

Table S3. Compound solubility in phosphate buffered saline (PBS) in pH of 7.4 at 37 °C for 1 h and GSH binding in the same conditions and recorded at 1 h or 24 h.

**Compounds**	**Solubility (mg/mL)**	**GSH binding (% rel. to CTRL)**	
		**1 h**	**24 h**
cis-$[Pt(MQ)(PPh_3)_2]PF_6$ (**1**)	1.5	40	55
$[Pt(dppm)(MQ)]PF_6$ (**2**)	1.3	43	66
$[Pt(dppe)(MQ)]PF_6$ (**3**)	1.0	0.7	38
$[Pt(dppp)(MQ)]PF_6$ (**4**)	1.9	1.0	39
$[Pt(dppb)(MQ)]PF_6$ (**5**)	0.75	10.7	52
$[Pt(dppf)(MQ)]PF_6$ (**6**)	1.9	6.5	43
cis-$[Pt(MQ)(PTA)_2]PF_6$ (**7**)	0.41	8.0	39
$[Pd(dppm)(MQ)]PF_6$ (**8**)	0.35	9.1	11.7
$[Pd(dppe)(MQ)]PF_6$ (**9**)	0.50	4.7	13.9
$[Pd(dppp)(MQ)]PF_6$ (**10**)	0.50	2.5	14.7
$[Pd(dppb)(MQ)]PF_6$ (**11**)	0.20	4.7	31.8
$[Pd(dppf)(MQ)]PF_6$ (**12**)	0.50	3.9	4.6
$[Pd(bipy)(MQ)]PF_6$ (**13**)	2.0	3.8	17.9
cis-$[PtCl(QN)(PPh_3)_2]PF_6$ (**14**)	N.D.	11.2	32
Mefloquine (MQ)	0.24	1.2	2.1
$[Pt(dppe)Cl_2]$ (**15**)	0.1	4.9	38

Table S4. Association constant (log *K*) for hemin, hypochromism of Soret's hemin band (Δε, %) and β-hematin inhibitory activity (BHIA) for the MQ-metal complexes.

**Compound**	**Log *K*** [a]	**Δε (%)**[b]	**BHIA, IC_{50} in mM (rel.)** [c]
cis-$[Pt(MQ)(PPh_3)_2]PF_6$ (**1**)	4.64±0.03	53.68±0.34	0.64±0.02 (3.0)
$[Pt(dppm)(MQ)]PF_6$ (**2**)	4.79±0.04	51.57±0.26	0.49±0.04 (3.9)
$[Pt(dppe)(MQ)]PF_6$ (**3**)	4.83±0.05	49.83±0.60	0.57±0.02 (3.4)
$[Pt(dppp)(MQ)]PF_6$ (**4**)	4.87±0.07	55.43±4.07	0.43±0.01 (4.5)
$[Pt(dppb)(MQ)]PF_6$ (**5**)	4.73±0.05	51.57±0.15	0.61±0.03 (3.2)
$[Pt(dppf)(MQ)]PF_6$ (**6**)	4.60±0.03	53.60±0.29	0.57±0.02 (3.4)
cis-$[Pt(MQ)(PTA)_2]PF_6$ (**7**)	4.88±0.03	30.69±6.41	1.01±0.05 (1.9)
$[Pd(dppm)(MQ)]PF_6$ (**8**)	4.72±0.07	39.40±0.85	0.64±0.02 (3.0)
$[Pd(dppe)(MQ)]PF_6$ (**9**)	4.71±0.09	36.38±0.27	0.52±0.01 (3.7)
$[Pd(dppp)(MQ)]PF_6$ (**10**)	4.86±0.05	38.96±0.25	0.30±0.02 (6.4)
$[Pd(dppb)(MQ)]PF_6$ (**11**)	4.71±0.05	39.34±0.47	0.29±0.04 (6.6)
$[Pd(dppf)(MQ)]PF_6$ (**12**)	5.15±0.09	59.02±0.79	0.41±0.02 (4.7)
$[Pd(bipy)(MQ)]PF_6$ (**13**)	5.03±0.05	45.80±0.48	0.87±0.02 (2.2)
Mefloquine (MQ)	4.51±0.17	33.56±2.45	1.92±0.02
Chloroquine (CQ)	5.21±0.13	56.91±0.22	0.40±0.02 (4.8)
Primaquine (PQ)	N.D.	N.D.	> 2.0

[a] Association constant to [Fe(III)-PPIX] (hemin). Values are median ± S.E.M. of three independent experiments.
[b] Percentage in hypochromicity of drug binding to the hemin. Values are mean ± S.D. of one single experiment.
[c] β-hematin formation upon incubation with compounds and determined after 48 h. Values in parentheses correspond to relative activity to MQ. Values are median ± S.E.M. of three independent experiments. Values in parenthesis are the ratio IC_{50} for MQ/IC_{50} of the compound.
N.D. = not determined, due to the lack of affinity for hemin.

Table S5. Cytotoxicity in mammal cells **of the** frontrunner metal complexes with MQ, Pt (**3**) and Pd (**9**).

**Compounds**	**Cell lineages, CC_{50} ± S.D. [μM][a]**		
	**HMEC-1**	**BMDM**	**HepG2**
MQ	9.86 ± 2.2	18.22 ± 3.5	44.9 ± 3.0
Pt (**3**)	3.42 ± 0.85	11.78 ± 1.3	29.9 ± 4.1
Pd (9)	N.D.	N.D.	57 ± 5.0
Pt (**15**)	33.82 ± 7.4	120.4 ± 0.3	> 80

[a]Cytotoxicity determined after 72 h incubation by AlamarBlue. Data are means and S.D. of two experiments, using three technical replicates. HMEC-1 = human microvascular endothelial cells. BMDM = mouse C57Bl/6 bone marrow derived macrophages. HepG2 = human hepatocellular carcinoma cells.

Table S6: Examination of the activity for mefloquine–metal conjugates across a panel of different strains and their speed of inhibitory activity on *P. falciparum* growth.

**Compound**	**Asexual blood stages of *P. falciparum* (asynchronous), IC_{50} ±S.D. [nM]**				**Sexual stages, IC_{50} ±S.D. [nM]**[c]
	**NF54** [a]	**K1** [a]	**MQ-resistance** [b]		
			**W2**	**TM91C235**	
Pt (**3**)	7.4±1.1	1.7±0.6	8.3±0.3	40.6±12.7*	2700±1100
Pd (**9**)	12±3.4	3.2±0.6	5.4±0.6	34.6±10.0*	8900±2000
[Pt(dppe)Cl_2] (**15**)	> 15000	> 15000	N.D.	N.D.	22400±1200
MQ	10±1.0	3.8±1.0	6.9±2.3	34.4±3.5*	3800±1150
CQ	7.5±0	180±15	N.D.	N.D.	> 2000
DHA	3.9±0.5	1.6±0.2	2.3±1.5	2.8±1.5	N.D.
MB	N.D.	N.D.	N.D.	N.D.	38±14

		**Median in recrudescence time (in days)**		
**Compd.**	Conc. [nM]	**NF54**	**W2**	**TM91C235**
ATO	1000	11	N.D.	N.D.
CQ	800	N.D.	3	N.D.
MQ	1000	>25	24	11[#]
Pt (**3**)	1000	>25	>25	24

	**Speed of activity against NF54 strain of *P. falciparum*, IC_{50} [nM]**[d]				
**Compd.**	**24 h**	**48 h**	**72 h**	**Ratio** [e]	**Conclusion**
ATO	>600	5.9	0.88	6.7	Slow
Pt (**3**)	>300	20.8	16.1	1.2	Relatively fast
Pd (**9**)	>300	28.5	22.2	1.2	Relatively fast
MQ	>300	16.9	17.8	0.9	Relatively fast
DHA	8.3	5.9	2.8	2.1	Fast

[a] Assessment of inhibitory breadth in asynchronous parasites after 72 h incubation by [^{3}H]-hypoxanthine incorporation. NF54 is drug-sensitive strain; K1 is multidrug-resistant strain. [b] Assessment of inhibitory breadth in MQ-susceptible (W2 strain) and MQ-resistant (TM91C235 strain) in asynchronous parasites after 72 h incubation by pLDH readout. [c] Assessment of inhibitory activity against gametocytes IV/V of 3D7 *P. falciparum* after 72 h incubation by luciferase readout. [d] Assessment of inhibitory activity against ring stages of NF54 *P. falciparum* after 24, 48 and 72 h of incubation and determined by pLDH readout. [e] IC_{50} (48 h) / IC_{50} (72 h). [a,b,c] Data are the mean ± S.D. of at least two independent experiments. CQ = chloroquine; DHA = dihydroartemisinin; MB = methylene blue; ATO = atovaquone. *$p<0.05$, unpaired and nonparametric Mann–Whitney rank test (TM91C235 strain *versus* W2 strain). #$p<0.05$, log-rank test (Mantel-Cox) for TM91C235 strain between MQ *versus* Pt (**3**).

Table S7: Inhibitory effects on the enzymatic activity of recombinant flavoproteins *P. falciparum* thioredoxin reductase (*Pf*TrXR); human thioredoxin reductase (*h*TrxR1) and *S. mansoni* thioredoxin-glutathione reductase (*Sm*TGR).

Compound	Thioredoxin reductases, IC_{50} in [nM][a]			*S. mansoni*, LC_{50} in [µM][b]	
	***Pf*TrxR**	***h*TrxR1**	***Sm*TGR**	**NTS**	**Adult worms**
Pt (**3**)	72±6.0	88±10	55±20	0.52±0.36	10.5±0.55
Pd (**9**)	19±2.6	1.4±0.7	0.88±0.21	27.6±6.8	>30
Pd (**13**)	14±11	0.17±0.1	5.5±1.0	>30	>30
MFQ	>66700	>66700	>66700	6.2±0.23	15.7±0.95
E-MQ	>66700	>66700	>66700	>30	>30
T-MQ	>66.7	47300±4600	>66700	15.8±0.4	23.3±0.98
[Pt(dppe)Cl_2] (15	31±2.9	0.69±0.11	0.71±0.7	>30	>30
PZQ	N.D.	N.D.	N.D.	27.1±2.27	32.8±4.07

[a] Values are in µM and the mean of one experiment, where each drug was tested in five different concentrations in triplicate. [b] Antiparasitic activity in NTS and adult stages of *S. mansoni,* was determined after 24 h of drug exposure. Pt(Cl_2) = [Pt(dppe)Cl_2]; PZQ = praziquantel; NTS = newly transformed schistosomula.

Table S8: Suppressive Peters test (treatment initiated 3 h post-infection) on parasitemia and animal survival in NK65 strain of *P. berghei*-infected Swiss mice (male).

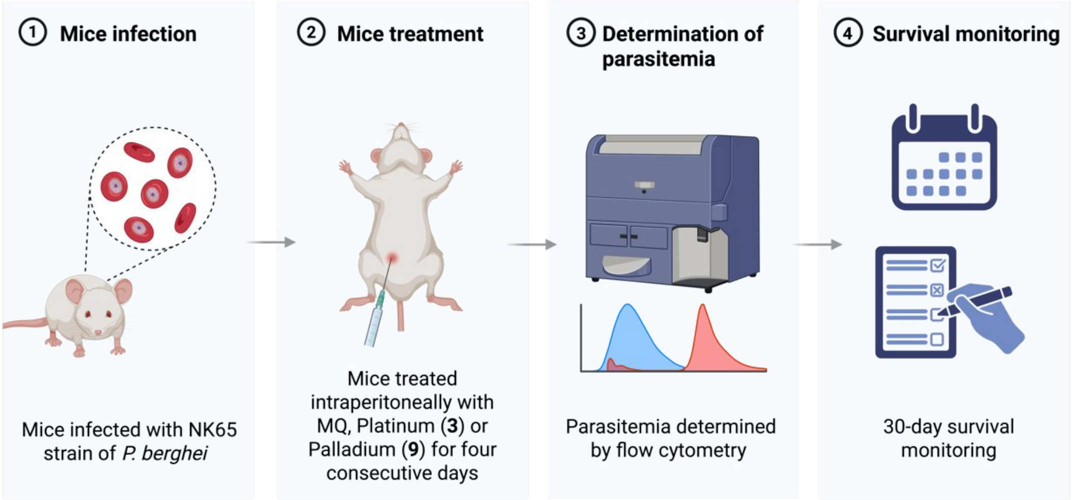

Compounds	Dose (intraperitoneal; i.p.) [a]		% Parasitemia reduction [b]	Survival [c]
	mg/kg/day	µmol/kg/day		
Vehicle (CTL)	-	-	-	0/20
Mefloquine (MQ)	4.4	11	78.2±6.6	0/5
	8.8	22	95.2±0.7	2/5
	12.0	33	100	8/10
Pt (**3**) (WV-41)	6.2	5.7	49.9±10.7	0/5
	12.5	11	92.8±6.5	4/5
	25	22	100	10/10
Pd (**9**) (WV-60)	12.5	12	55.1±9.1	0/5
	25	24	96.0±1.4	8/10

[a] An injection of 100 µL volume was given daily.
[b] Parasitemia was determined by flow cytometry using SY61 for parasite staining; values are mean and S.D. normalized for untreated group (vehicle).
[c] Animal survival monitored daily for up to 30 days post-infection (DPI).

Table S9: Curative Thompson test (treatment initiated on 3rd post-infection) on parasitemia and animal survival in NK65 strain of *P. berghei*-infected Swiss mice (male).

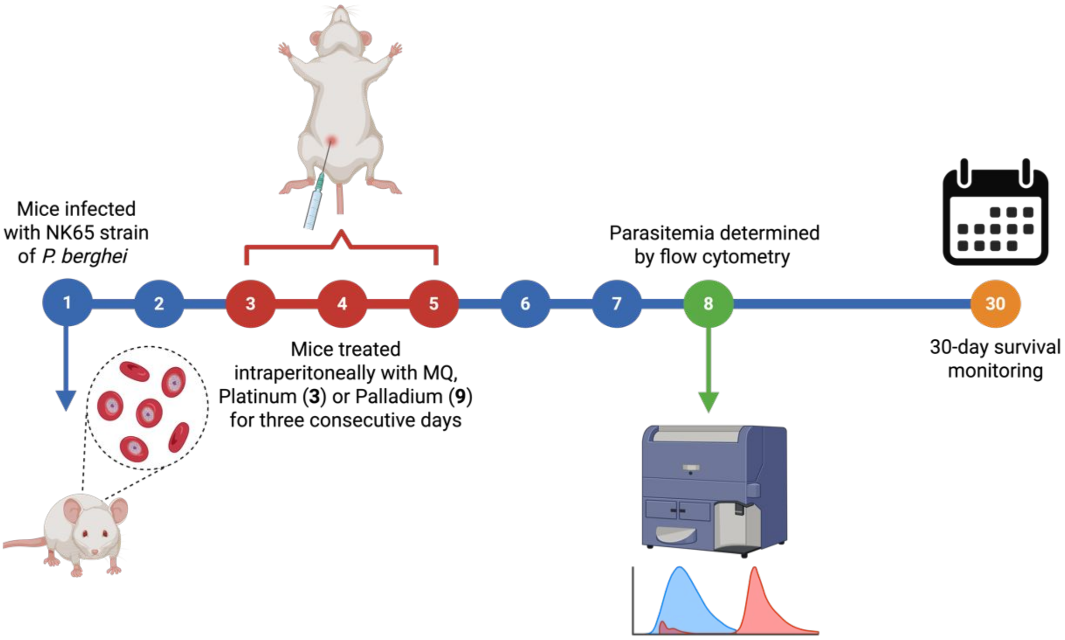

**Compounds**	**Dose (intraperitoneal; i.p.)** [a]		**% Parasitemia reduction** [b]	**Median survival (days)**	**Survival** [c]
	**mg/kg/day**	**µmol/kg/day**			
Vehicle (CTL)	-	-	-	9	0/10
Mefloquine (MQ)	10	26	> 99	20.5	2/10
Pt (**3**) (WV-41)	30	26	> 99	26.5	4/10
Mefloquine+Artesunate (Reference treatment)	55 mg/kg of Mefloquine + 100 mg of Artesunate		> 99	>30	5/5

[a] Injection of 100 µL volume was given daily.
[b] Parasitemia was determined by flow cytometry using SY61 for parasite staining; values are mean and S.D. normalized for untreated group (vehicle).
[c] Animal survival monitored daily for up to 30 days post-infection (DPI).

Table S10: Cumulative chemical transformation (%) of metal complexes (inferred by $^{31}P\ \{^{1}H\}$ NMR spectra and further confirmed by ^{1}H NMR and $^{13}C\{^{1}H\}$ NMR) and for Mefloquine (by ^{1}H NMR).

**Complexes**	**Cumulative chemical transformation (%) at 72 h of incubation**		
	**DMSO-*d*$_6$**	**DMSO-*d*$_6$/D$_2$O**	**DMSO-*d*$_6$/cell culture**
Mefloquine (**MQ**)	< 2.0	< 2.0	< 2.0
[Pt(dppe)Cl$_2$] (**15**)	N.D.	N.D.	23.99
Pt(dppe)(MQ)]PF$_6$ (**3**)	< 2.0	< 2.0	< 2.0
Pd(dppe)(MQ)]PF$_6$ (**9**)	< 2.0	10.11	44.07
cis-PtCl(QN)(PPh$_3$)$_2$]PF$_6$ (**14**)	< 2.0	< 2.0	23.99

*Conditions: pure DMSO-*d*$_6$; a DMSO-d$_6$ /D$_2$O mixture (70:30 v/v) or at a mixture of a DMSO-d$_6$ /RPMI cell culture (70:30 v/v) at 298K.

A

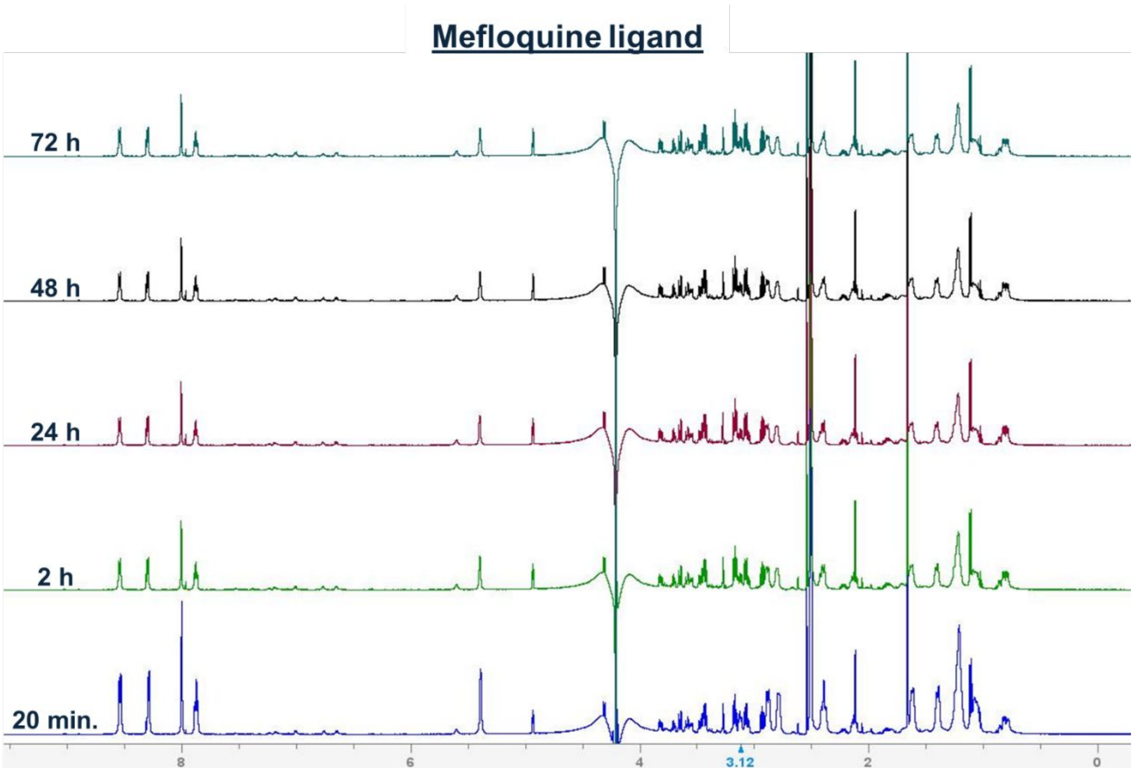

B

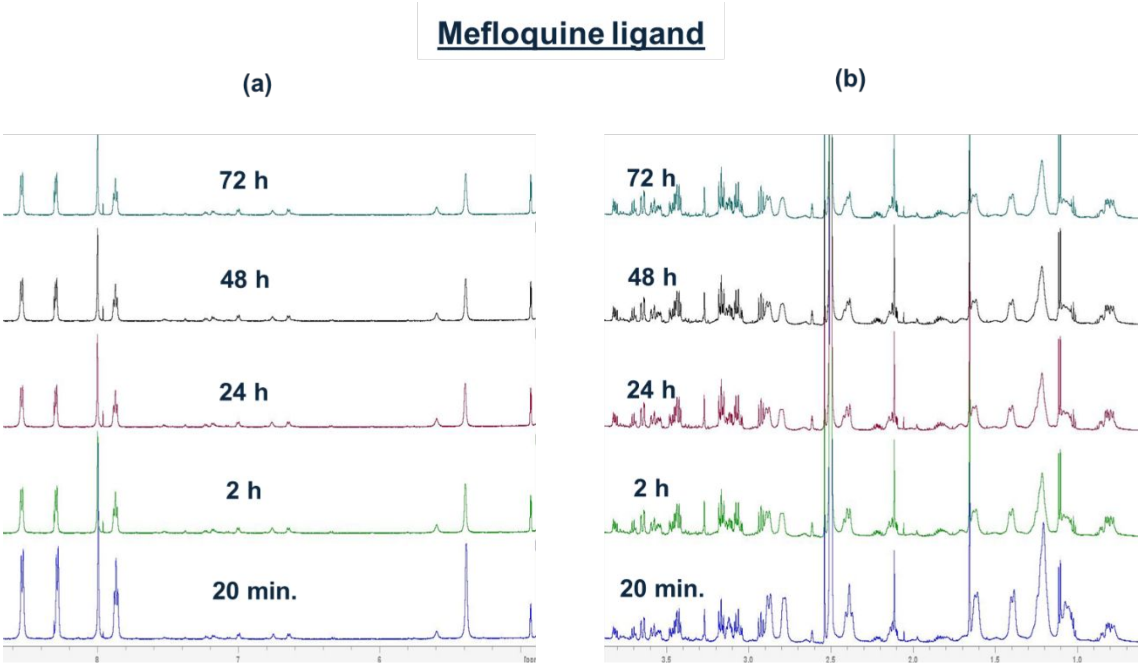

Figure S11. Panel A) ^{1}H NMR spectra in deshielding region of Mefloquine (MQ) in DMSO-d_6/RPMI culture medium mixture (70:30) in different times (20 min., 2h, 24h, 48h and 72h) at 298K. Panel B) ^{1}H NMR spectra in shielding region of Mefloquine (MQ) in DMSO-d_6/RPMI culture medium mixture (70:30) in different times (20 min., 2h, 24h, 48h and 72h) at 298K.

A

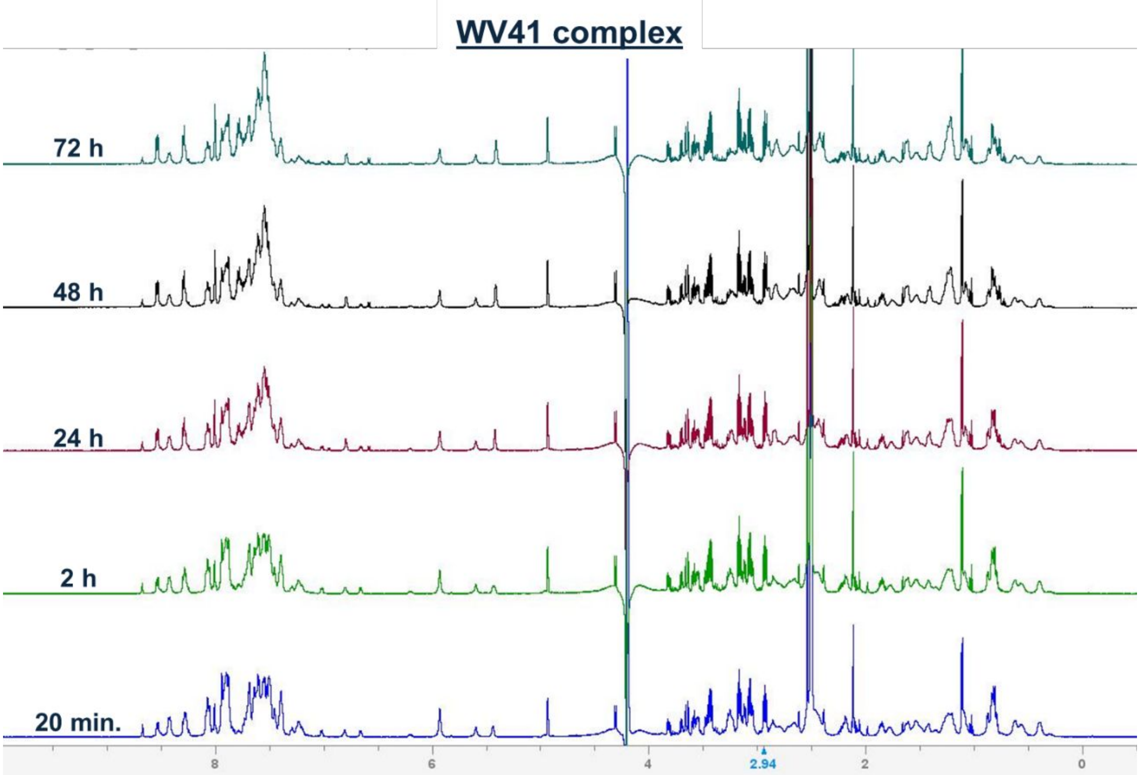

B

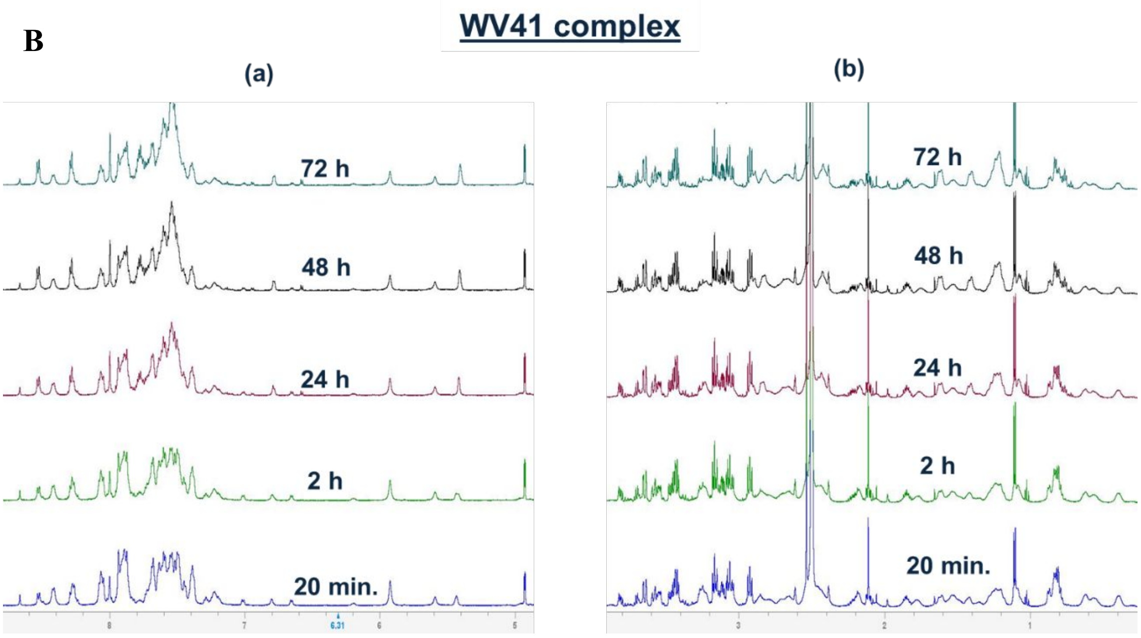

Figure S12. Panel A) ^{1}H NMR spectra in the deshielding region of complex $[Pt(dppe)MQ]PF_6$ Pt (**3**) in DMSO-d_6/RPMI culture medium mixture (70:30) in different times (20 min., 2h, 24h, 48h and 72h) at 298K. Panel B) ^{1}H NMR spectra in the shielding region of complex $[Pt(dppe)MQ]PF_6$ Pt (**3**) in DMSO-d_6/RPMI culture medium mixture (70:30) in different times (20 min., 2h, 24h, 48h and 72h) at 298K.

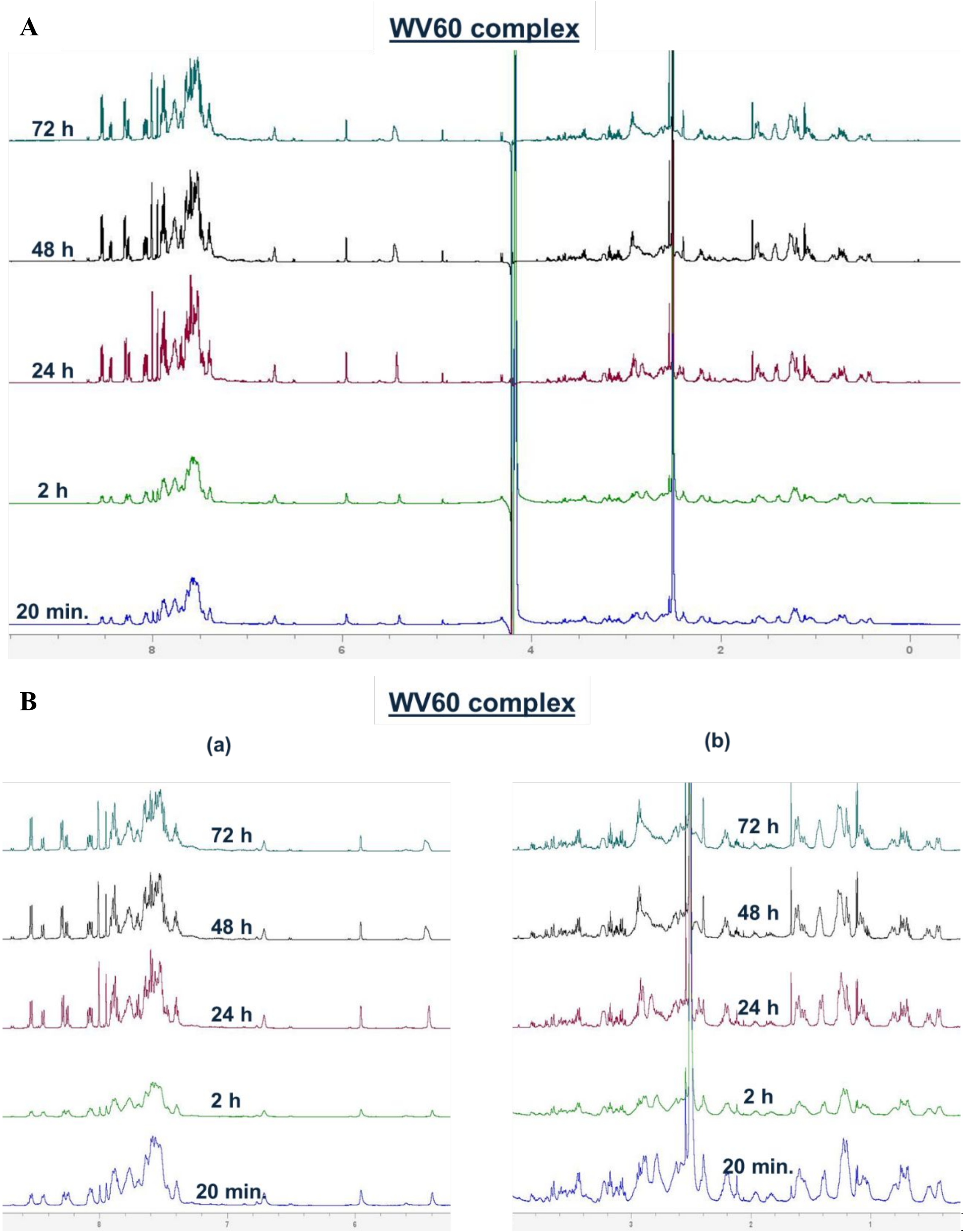

Figure S13. Panel A) ^{1}H NMR spectra in the deshielding region of complex [Pd(dppe)MQ]PF_6 Pd (**9**) in DMSO-d_6/RPMI culture media mixture (70:30) in different times (20 min., 2h, 24h, 48h and 72h) at 298K. Panel B) ^{1}H NMR spectra in the shielding region of complex [Pd(dppe)MQ]PF_6 Pd (**9**) in DMSO-d_6/RPMI culture media mixture (70:30) in different times (20 min., 2h, 24h, 48h and 72h) at 298K.

COMPLEMENTARY DESCRIPTION OF RESULTS

A full description of physical-chemical and spectral characterization of the $[M^{II}(L)(MQ)]PF_6$ (**1-13**) is provided in this section.

The UV-vis spectra of the complexes (**1-13**) displayed bands near to 304-320 nm, corresponding to π-π^* transitions of MQ, while the 264-268 nm bands were assigned to π-π^* transitions of phosphine and bipyridine. Two bands in the 270-280 nm region were attributed to as the metal-to-ligand charge transfer (MLCT) transitions from Pt or Pd (dπ) to the phosphine or bipyridine ligand (π^*), and as the ligand-to-metal charge transfer (LMCT) transitions, respectively. Moreover, it was observed bands in 346-410 nm range were observed and assigned as the *d-d* transitions of the metal centers. For complexes (**6)** and (**12),** these bands were displayed at 434 and 446 nm respectively, which we attributed to the influence of the employed phosphine (dppf). Finally, it is worth to mention that not all the MLCT, LMCT and *d-d* transitions in the metal precursors were observed in the complexes (**1-13**), most likely due to their low intensity, or those were hidden by the intra-ligand transitions of MQ.

The IR spectrum of the complexes (**1-13**) displayed a band at 3240-3280 cm^{-1} corresponding to NH stretching vibrations, indicating that after coordination the hydrogen of NH group is kept in the molecule. The band corresponding to C=N vibrations of MQ in the complexes was observed between 1596-1601 cm^{-1}, while in the free ligand, it was at observed at 1587 cm^{-1}. A band at 845 cm^{-1} was assigned to P-F vibrations of the PF_6^- counter-anion. In the far-IR spectra of the complexes (**1-12, 14**), it was observed a band at 548-592 cm^{-1} corresponding to M-P vibrations, while in the case of the complexes (**1**) and (**7**), those bands were observed as two distinct bands, most likely due the presence of two monophosphinic ligands in *cis* position to each other.

The NMR signals were assigned on the basis of 1D (^1H, $^{13}C\{^1H\}$, $^{31}P\{^1H\}$) and 2D (COSY, HSQC and HMBC), enabling us to identify the signals of MQ, phosphine and bipyridine. The chemical shift of each signal of the ligands upon metal coordination is shown in **Table S1** and were used for

inferring the mode of bonding of MQ to the metal. The signals in the ^{1}H NMR spectrum which displayed the greater modification correspond to the hydrogens located in the region of bonding to the metallic center. In the piperidine ring, these signals corresponding to NH and H3' and those underwent the greatest shielding, which we explained this by a gain in electronic density of the nitrogen atom after it is coordinated, shielding the atoms of nearest hydrogen. In the case of metal binding by the oxygen atom, this has caused a strong electronic and spatial change under the surrounding hydrogens of H1", H5', H6', as well as for the quinolinic hydrogen H3. Based on the shielding of the NH signal from MQ as well as the absence of the OH signal, we have interpreted that the binding of MQ to the metal is through the NH of the piperidine ring and the oxygen atom of the former OH group (which is deprotonated), therefore, MQ behaving as a *N,O*-bidentate ligand.

The $^{13}C\{^{1}H\}$ NMR spectra have not only confirmed the presence of all ligand carbons as well as the deshielding of the atoms near to the coordination site such as the C3', C1' and C1'' atoms, but the spectra also showed the duplication of the signals of the carbons atoms of the piperidine ring for the complexes **2**, **3**, **6**, **8**, **9** and **13**. In Figure 3D, this is shown for the complex **8**, This is suggestive the presence of two conformational isomers generated by the combination of the enantiomers of mefloquine ligand (*S*,*R* and *R*,*S*) with the new stereogenic center created on the nitrogen atom and the chair flip of the piperidine ring.

In the $^{31}P\{^{1}H\}$ NMR spectra for phosphine complexes **1-12, 14** (**Figure S3**) two doublets corresponding to the phosphorus atoms are observed, which have a different chemical environment, - a phosphorus atom (P_a) in a *trans* position to the nitrogen atom of the piperidine ring of mefloquine, and another phosphorus atom (P_b), in *trans* position to the oxygen atom. This variation can be attributed to the difference in the electronegativity of the oxygen atom when compared with nitrogen atom, causing to the oxygen atom deshielding the phosphorus atom in *trans* position to it (P_b). In the case of complex **14**, (P_a) correspond to phosphorus atom in a *trans* position to the nitrogen atom of quinolinic ring and (P_b) correspond to phosphorus atom in a *trans* position to the chloride atom. A

septet is also observed for all the compounds (**1-14**) at -144.5 ppm, which corresponds to the counterion PF_6^-, necessary to neutralize the charge of the cationic complexes. In the case of the platinum complexes (**1-7, 14**), the satellite signals are observed on both sides of each phosphorus signal, due to the couplings of the ^{31}P and ^{195}Pt nuclei.

The ESI(+)-MS and ESI(+)-MS-MS spectra for the complexes (as shown for the complex **7** in the **Figure S4**) displayed a signal corresponding to a loss in the hexafluorophosphate ion $[M-PF_6]^+$, followed by a loss in the mefloquine ligand $[M-PF_6-MQ]^+$ and a peak corresponding to the free ligand $[MQ+H]^+$.

The crystal structures of complexes Pt (**2**), Pt (**6**) and Pt (**9**) were determined by single-crystal X-ray diffraction. These three complexes have shown a distorted plane square geometry. Moreover, crystallography has confirmed the coordination of the mefloquine ligand to the metal center through the nitrogen of piperidine ring and the oxygen atom. Complexes Pt (**2**) and Pt (**6**) crystallized in the space group $P2_1/c$ and $P2_12_12_1$, respectively, where it is observed only one enantiomer of the mefloquine ligand coordinated to the metal center. In contrast, complex Pd (**9**) crystallized in the space group P-1, showing the presence of the compounds formed with the two enantiomers of the mefloquine ligand (*S*,*R* and *R*,*S*). This behavior was also observed in solution, as shown in the ^{13}C{^{1}H} NMR spectra.

An inspection on the bond lengths for the complexes Pt (**2**), Pt (**6**) and Pd (**9**) (**Table S2**) reveals that the length of the M-N bond is considerably greater than that of the M-O bond, which may be due to the fact that the alkoxide ligand is used a negative charge to make a covalent bond with the metal center, strengthening this bond and therefore reducing its length. In the case of the M-P bonds, the platinum complexes (**2** and **6**) show that the bond P1-Pt (in *trans* position to N) is higher in length that the bond P2-Pt (in *trans* position to O), however, the palladium complex (**9**) has a contrast behavior, this difference can be attributed to disparity in the size of the metal centers.

For the interpretation of the pharmacological assays, it was necessary to determine the stability of complexes in solution (**Table S9**). This was monitored by NMR, where assays were performed in three conditions: a) DMSO, b) in a mixture of DMSO/D_2O and c) performed in a mixture of DMSO:cell culture at 70:30 v/v ratio at 0 to 72 h, which is a timeframe that coincides with the overall drug incubation in the *in vitro* assays. As it is well known that an interaction with aqueous medium can lead to a rapid ligand exchange reaction. In the **Figure S5** is observed that the NMR spectra profiled in DMSO and DMSO/D_2O for the compounds **3** and **9** don't display significant variations when compared with the fresh solutions.

In the mixture DMSO:cell culture at 70:30 v/v, Mefloquine (MQ) was stable during incubation for up to 72 h (**Figure S11**). Metal precursor Pt Cl_2 of formula [Pt(dppe)Cl_2] (**15**) showed a small transformation after dissolution as inferred by $^{31}P\{^1H\}$ NMR spectra (**Figure S6C**). However, no obvious transformation was inferred by in the aromatic region on the ^1H NMR but rather than in the aliphatic region, suggesting that ligand exchange reactions took place by scrambling chloride by DMSO. After 20 min, 5.11% of [Pt(dppe)Cl_2] was transformed and 23.99% of it was transformed after 2 h. These portions remained constant during the remaining 72 h incubation period.

The complex Pt (**3**) [Pt(dppe)MQ]PF_6 was stable under all of the timeframe monitored by both ^1H NMR and $^{31}P\{^1H\}$ NMR spectra (**Figures S12 and S6A**). Minor and marginal peaks at the $^{31}P\{^1H\}$ NMR spectra (**Figure S6A**) were observed, similar behavior was showed by the complex Pt (**14**) *cis*-[Pt(PPh_3)$_2$(QN)Cl]PF_6 (**Figure S6D**). On the other hand, the stability of Pd (**9**) [Pd(dppe)MQ]PF_6 was different than its counterpart Pt (**3**). After addition of the aqueous medium, 5.30% of Pd (**9**) was transformed as inferred by $^{31}P\{^1H\}$ NMR spectra (**Figure S6B**). This proportion remained constant for 2 h. However, after 24 h, the proportion substantially changed to 14.01 % and finally to 44.07 % at 72 h. The stability of Pd (**9**) inferred by $^{31}P\{^1H\}$ NMR spectra was also mirrored by ^1H NMR (**Figures S13**). Showing the classical behavior of palladium compounds, which are characterized by being less stable both kinetic and thermodynamic than their platinum counterparts.

1.0 Cell-free assays

1.1 Determination of aqueous solubility

Solubility in water was evaluated at 37 °C in pH 7.4 in phosphate buffered saline (PBS). Each complex was weighed into individual screw cap polypropylene vials of HPLC grade and aqueous media was added to result in a 2 mg/mL. Each vial was vortexed on an orbital mixer for 1 h at 600 rpm. After centrifugation and filtration in a 0.22 μm syringe filter, aliquots of 250 μL were collected, digested in acid and analyzed in an ICP-MS (details described below). The amount of Pt per mL was expressed as the aqueous solubility of the respective complex.

1.2 Determination of aqueous stability by NMR

Each metal complex (0.021 mmol) was dissolved in 500 μL of DMSO-d_6 or 350 μL of DMSO-d_6 and 150 μL of D_2O and added to an NMR tube. This was stirred and then sealed. ^1H and $^{31}P\{1H\}$ NMR spectra (14.1 T, 600 MHz) were recorded at different times in a Bruker Biospin NEO model spectrometer and equipped with a 5.0 mm BBI probe. The rate of chemical transformation (%) was estimated as the decrease in peak intensity from each time point in comparison to a fresh prepared solution (referred to as the 0 h). As the signal of PF_6 remains unaltered, it was used as an internal standard.[1]

1.3 Stability in the presence of reduced glutathione (GSH) by fluorescence assay

In a 96-well microplate, a volume of 170 μL/well of a solution of reduced glutathione (GSH) (Sigma-Aldrich, St. Louis, MO, USA) at 0.5 μM dissolved in PBS (pH 7.2) was aliquoted. Then, a 10 μL solution of each complex in DMSO was added in in the respective wells to a final concentration of 0.5 μM. The plate was sealed and incubated at 37 °C for 1 or for 24 h, and then, the contents were transferred to an opaque microplate containing 10 μL of a solution of monochlorobimane MClB

(Sigma-Aldrich) at 2.0 mM previously dissolved in methanol. After 1 h incubation at room temperature, fluorescence was recorded (λ_{ex} = 360 nm, λ_{em} = 440 nm) in a microplate reader. A blank subtraction was performed prior to the addition of MClB, and controls included untreated wells (no complex) and no GSH. The results were expressed as a percentage of inhibition of GSH binding to MClB in comparison to control without compound. Two technical replicates of each compound concentration were employed.

1.4 Stability in the presence of GSH and GSSG by NMR analysis

Metal complex (0.021 mmol) was dissolved in 300 µL of DMSO-d_6 and an amount of 0.021 mmol of reduced glutathione (GSH) previously dissolved in 150 µL of D_2O was added to the mixture in an NMR tube, then it was well mixed by vortex and sealed. NMR spectra were recorded at different times. The same reactions were performed but containing GSH disulfide (oxidized, GSSG). $^{31}P\{1H\}$ NMR spectra (14.1 T, 600 MHz) were recorded in a Bruker Biospin NEO model spectrometer and equipped with a 5.0 mm BBI probe. The rate of chemical transformation (%) was estimated as described above.

1.5 Interactions with ferriprotoporphyrin [$Fe^{(III)}$PPIX]

A stock solution of hemin (3.5 mg of hemin chloride in 10 mL of DMSO) was prepared, from which solutions of Fe(III)PPIX (pH 7.5) 40% v/v were prepared, mixing 140 µL of the stock solution, 3.68 mL of DMSO, 1 mL of 0.2 M trizma buffer and 5 mL of water. The complexes were also prepared in this 40% DMSO-Trizma solution with concentrations of 0.3 to 0.5 mM. 2 mL of hemin solution were added to the cuvette and the reference cuvette contained a 40% v/v DMSO – 0.02 M trizma solution (pH 7.5). Aliquots of complex solutions were added, both in the cuvette containing hemin and in the reference cuvette, in order to subtract the absorbance of the complexes from the absorption spectrum. The binding affinities were obtained using the equation:

$$A = \frac{A_0 + A_\infty K[C]}{1 + K[C]}$$

for a 1:1 interaction model, fitting the curve using the non-linear least squares method, where A0 is the initial absorbance without the presence of the complex, A∞ is the absorbance of the complex-hemin adduct at saturation, A is the absorbance of each point in the titration and *K* is the association constant. Three independent experiments were performed.

1.6 Inhibition of β-hematin formation by UV-vis spectroscopy

A solution of hemin chloride (52 mg in 10 mL of DMSO) was prepared and distributed in a 96-well plate (50 μL/well), solutions of the complexes and mefloquine at different concentrations (10mM-10 μM) were distributed in the 96-well plate (50 μL/well), then acetate buffer (100 μL, 0.4 M, pH 4.4) was added to initiate hematin formation. Subsequently, the plates were incubated for 48 h at 37°C, and centrifuged at 4000 rpm for 20 min, the supernatant was discarded and the solid was washed with 200 μL of DMSO, centrifuged again under the conditions described above, was repeat the washing and finally the solid was dissolve in 200 μL of 0.2 M NaOH and solubilized with 100 μL of 0.1 M NaOH for reading by UV-Vis at 405 nm. The results obtained are expressed as percentage of β-hematin inhibition.

2.0 Pharmacology

2.1 Drugs and dilutions

Mefloquine and Chloroquine were supplied by FarManguinhos (Rio de Janeiro, Brazil). Atovaquone and Dihydroartemisinin were purchased from Sigma-Aldrich. In all assays, Mefloquine free base was employed and denoted as mefloquine (MQ). All drugs were dissolved in DMSO (PanReac, Barcelona, Spain) prior to use and then diluted in culture medium. The final concentration of DMSO was less than 0.5 % in all *in vitro* experiments.

2.2 Parasites and cell culture

CQ-sensitive 3D7, NF54 and CQ-resistant W2 and K1 strains of *P. falciparum* were cultivated in human O^+ erythrocytes (donated by HEMOBA, Salvador, Brazil) at 5 % hematocrit with daily maintenance in RPMI-1640 medium (Sigma-Aldrich) supplemented with 5 % (v/v) heat-inactivated human plasma (donated by HEMOBA, Salvador, Brazil), 25 mM 4-(2-hydroxyethyl)-1-piperazineethanesulfonic acid (HEPES, ChemCruz, Dallas, TX), 300 μM hypoxanthine (MP Biomedicals, Santa Ana, CA), 11 mM glucose (Sigma-Aldrich) and 10 μg/mL of gentamicin (Life, Carlsbad, CA). Five days prior to use, *P. falciparum* was cultivated without hypoxanthine and synchronized to rings by 5 % D-sorbitol (USB, Santa Clara, CA). NK65 strain of *P. berghei* was routinely maintained in Swiss mice. J774 macrophages were cultured in Dulbecco′s modified Eagle′s medium (DMEM) (Sigma-Aldrich) supplemented with 10 % (v/v) fetal bovine serum (FBS, Gibco, Gaithersburg, MD) and 50 μg/mL of gentamicin (Life). HepG2 cells were cultured in RPMI medium (Sigma-Aldrich) supplemented with 10 % (v/v) fetal bovine serum (Gibco, Gaithersburg, MD) and 50 μg/mL of gentamicin (Life Technology).

2.3 Cell cytotoxicity

In 96-well plates, J774 cells were seeded (1.0×10^4 per well) in 100 μL of DMEM. Compounds were added 24 h later in a volume of 100 μL suspended in medium and the plates were incubated for 72 h at 37 °C and 5 % CO_2. Each compound was tested in seven concentrations (50-0.78 μM), each one in triplicate. Gentian violet (Synth, Diadema, Sao Paulo, Brazil) was used as positive control, while untreated cells were employed as negative controls. Then, 20 μL of AlamarBlue (Life Technologies) was added and plates were incubated for 4-6 h. Colorimetric readings were performed at 570 and 600 nm using SpectraMAx 190 instrument (Molecular Devices, Sunnyvale, CA). This same procedure was performed for HepG2 cells cultivated in RPMI medium, BMDM cultivated in DMEM and HMEC-1 cultivated in MCDB 131 medium. Mean CC_{50} values were calculated using data from three independent experiments.

2.4 Hemolysis assay

Fresh uninfected human O^+ erythrocytes were washed three times with sterile phosphate-buffered saline (PBS), adjusted to 1 % hematocrit and 100 μL dispensed in a 96-well round bottom plate. Then, 100 μL of drugs previously dissolved in DMSO and suspended in PBS was dispensed in the respective wells. Each drug was tested at seven concentrations (100-1.5 μM), assayed in triplicate. Untreated cells received 100 μL of PBS containing 0.5 % (v/v) DMSO (negative control), while positive controls received saponin (Sigma-Aldrich) at 1 % v/v. Plates were incubated for 1 h at 37 °C under 5 % CO_2. Plates were centrifuged at 1500 rpm for 10 min and 100 μL of supernatant were transferred to another plate, in which absorbance at 540 nm was measured using a SpectraMax 190 instrument. The percentage of hemolysis was calculated in comparison to positive and negative controls and plotted against drug concentration generated using GraphPad Prism. Two independent experiments were performed.

2.5 Screening of inhibitory activity for *P. falciparum* ring stages

Chloroquine-sensitive 3D7 and chloroquine-resistant W2 strains of *P. falciparum* at ring stages in RPMI were dispensed in a 96-well round bottom plate for 0.5 % parasitemia and 1.5 % hematocrit in 80 μL/well. Then, 80 μL of each compound (2.0-0.0015 μM) previously dissolved in DMSO and suspended in RPMI were dispensed into the respective wells. Each compound was tested at seven different concentrations, each concentration in triplicate. Untreated parasite wells received 80 μL of medium containing 0.5 % DMSO. Plates were incubated in a gas-tight box for 48 h at 37 °C under 3 % O_2, 5 % CO_2 and 91 % N_2 atmosphere. Then, 25 μL of [^{3}H]-hypoxanthine (0.5 μCi/well, PerkinElmer, Shelton, CT) in RPMI was added to each well and incubated for 24 h. Plates were frozen at -20 °C and subsequently thawed and the contents transferred to UniFilter-96 GF/B PEI coated plates (PerkinElmer) using a cell harvester. After drying, 50 μL of scintillation cocktail (MaxiLight, Hidex, Turku, Finland)

was added to each well, sealed and plates read in a liquid scintillation microplate counter (Chameleon, Turku, Finland). The % of inhibition was determined in comparison to untreated and inhibitory concentration for 50 % (IC_{50}) values were determined by using non-linear regression with Logistic equation available at OriginPro 8.5. Three independent experiments were performed. Additional tests of antiparasitic activity against the NF54 (drug-sensitive) and K1 (resistant to chloroquine and pyrimethamine) strains of *P. falciparum* (asynchronous culture) for selected compounds were performed by [^{3}H]-hypoxanthine incorporation assay as described above; in this case, two independent experiments were performed.

2.6 Antiparasitic activity for MQ-resistant strain of *P. falciparum*

Asynchronous parasites of TM91C235 (MQ-resistant) strain of *P. falciparum* were dispensed into a 96-well round bottom plate at 0.5 % parasitemia and 1.5 % hematocrit in 100 µL/well. Then, 100 µL of each compound (0.3-0.003 µM) previously dissolved in DMSO and suspended in RPMI were dispensed in the respective wells. Dihydroartemisinin (DHA) was employed as a positive control. Each compound was tested at seven different concentrations. Untreated parasite wells received 100 µL of medium containing 0.5 % DMSO. Plates were incubated in a gas-tight box for 72 h at 37 ^oC under 3 % O_2, 5 % CO_2 and 91 % N_2 atmosphere. Control experiments employed the isotype W2 strain. Plates were processed using the parasite lactate dehydrogenase (pLDH) method,[2] and IC_{50} values were calculated as described above. Three independent experiments were performed using two technical replicates. A parallel experiment was carried out using asynchronous parasites of the W2 strain (MQ susceptible) of *P. falciparum.*

2.7 Parasite recrudescence assay

Parasites of NF54, W2, and TM91C235 (MQ-resistant) strains of *P. falciparum* were synchronized at the ring stage by D-sorbitol treatment, followed by adjustment to 3 % parasitemia and 2 % hematocrit

in a 5.0 mL volume in 6-well plates, and treated with each drug for 48 h. Treatment was given at 1000 nM for MQ, atovaquone, and Pt (**3**), and chloroquine at 800 nM. Parasites were washed twice with RPMI medium with 0.5 % albumax before being placed in drug-free culture conditions. At day 0 that treatment was removed, parasitemia was determined every two days by Giemsa-staining of blood smears until the cultures reached a parasitemia of 3.0 % and this was defined as the recovery day. If no parasitemia recrudescence was observed for up to 30 days, the experiment was terminated. Two independent experiments were performed, and each treatment was performed using two replicates.

2.8 Antiparasitic activity for specific parasite stages of *P. falciparum*

Parasites of NF54 (drug-sensitive) strain of *P. falciparum* were synchronized using D-sorbitol. At rings and trophozoites stages, as determined by Giemsa staining, parasites in RPMI were dispensed in a 96-well round bottom plate for 0.5 % parasitemia and 1.5 % hematocrit in 100 μL/well. Then, 100 μL of each compound (0.3-0.003 μM) previously dissolved in DMSO and suspended in RPMI were dispensed in the respective wells. Each compound was tested in seven different concentrations. Untreated parasite wells received 100 μL of medium containing 0.5 % DMSO. Plates were incubated in a gas-tight box for 6 h at 37 °C under 3 % O_2, 5 % CO_2 and 91 % N_2 atmosphere. Parasites were then washed with RPMI-1640 medium before being placed in drug-free culture conditions with 10% human serum and incubated for 66 h. The control plate was left without the washing step, enabling continuous drug exposure throughout. Plates were processed using the pLDH method, and IC_{50} values were calculated as described above. Three independent experiments were performed using two technical replicates.

2.9 Antiparasitic activity for *P. falciparum* gametocyte stages

In a 96-well round bottom plate, 100 μL of 3D7elo1-pfs16-CBG99 gametocytes (stages IV/V) at 0.5-1 % parasitemia and 2 % hematocrit) were dispensed. Each drug was tested in triplicate, at seven

different concentrations. The DMSO concentration was not toxic for gametocytes. Methylene blue (MB) was used as positive control. Plates were incubated for 72 h at 37 ^{o}C under 3 % O_2, 5 % CO_2, 91 % N_2 atmosphere. After this time, 100 µL of culture medium was removed from each well to increase hematocrit, 70 µL of resuspended culture was transferred to a black 96-well plate and 70 µL of D-luciferin (1 mM in citrate buffer 0.1 M, pH 5.5) was added. Luminescence measurements were performed after 10 min with 500 ms integration time. The IC_{50} was extrapolated from the non-linear regression analysis of the concentration–response curve. The percentage of gametocytes viability was calculated as 100 × [(OD treated sample – OD blank) / (OD untreated sample – µc-blank)] where "blank" is the sample treated with 500 nM of MB that completely kills gametocytes.

2.10 Speed of antiparasitic activity against *P. falciparum*

The assay was performed as describe in the literature.[3] Briefly, each well of a 96-well plate was filled with 100 µL drug and 100 µL culture containing asynchronous *P. falciparum* (NF54 strain) parasites, which were mixed to obtain a final parasitemia of 1.5 % and 1.0 % hematocrit. The plates were incubated for 24, 48, and 72 h at 37°C with a standard gas mixture. Dihydroartemisinin (a fast-acting drug) was used as a positive control and atovaquone was used as a relatively slow-acting drug. Parasite viability was measured using the pLDH method and IC_{50} values were calculated as described above. Two independent experiments were performed using two technical replicates.

2.11 Quantification of reactive oxygen species in trophozoite stages of *P. falciparum*

The assay was performed as describe in the literature.[4] A volume of 500 µL of trophozoites of *P. falciparum* 3D7 strain at 3.0 % parasitemia and 1.0% hematocrit in RPMI was dispensed per well in a 12-well plate. CM-H2-DCFDA (Life Technology) at 5 µM suspended in medium was added to each well and incubated in the dark for 20 min. Then, drugs previously suspended in RPMI was added to the respective wells. Each drug concentration (500 nM) was tested in triplicate. The untreated parasites

received medium containing 0.5% DMSO. The plates were incubated for 4 h at 37 °C under 3% O_2, 5% CO_2, 91% N_2 atmosphere. The plates were centrifuged at 1500 rpm for 5 min, the supernatant was discarded and 200 µL of isotonic diluent was added and the samples were analyzed in a flow cytometer (LSRFortessa, BD). The gate of the infected cells was determined in comparison with the uninfected control. At least 200,000 events were acquired in the fluorescein isothiocyanate channel (488, 585 nm) for CM-H2-DCFDA. The analysis was performed using FlowJo (LLC) in three independent experiments.

2.12 Effects of MQ on *P. falciparum* redox homeostasis

P. falciparum 3D7 was propagated in RBCs (A^+) in RPMI 1640 medium supplemented with 0.5% Albumax, 9 mM glucose, 0.2 mM hypoxanthine, 2.1 mM L-glutamine, 25 mM Hepes, and 22 µg/ml gentamycin at 3.3% hematocrit and 37 °C in a gaseous mixture consisting of 3% O_2, 3% CO_2 and 94% N_2. Parasites were maintained under constant drug pressure with 5 nM WR99210. The effect of the compounds on *P. falciparum* was determined after 4 h and 24 h. For 4 h experiments, magnetically enriched trophozoite stage parasites (26-30 h post invasion) of $3D7^{[hGrx1\text{-}roGFP2]}$ (6-8% parasitemia) were used. 1.0 x 10^6 cells in 100 µL cell culture medium were placed into LoBind tubes. The parasites were treated with the compounds at 500 nM for 4 h and subsequently blocked with 2 mM *N*-ethylmaleimide (NEM) for 15 min at 37°C. For 24 h experiments, ring stage parasites (6-10 h post invasion, 6-8% parasitemia) were treated with compounds at 4xEC_{50}. Prior to enrichment, cysteines were blocked with 2 mM NEM. Cells were resuspended in pre-warmed Ringer´s solution for live cell imaging and measured in the Leica confocal system TCS SP5 with excitation wavelengths at 405 nm and 488 nm. All experiments included non-treated parasites as controls, and both fully oxidized and fully reduced parasites with 1 mM DIA and 10 mM DTT (2 min incubation) prior to blocking with NEM. The obtained ratio values were all related to the control (CTL) ratio value, which was set to 100.

The experiments were performed three times. Parasitemia was counted just once by using Giemsa-stained blood smears and represent approximate numbers.

2.13 Parasite harvesting for microscopy and spectroscopy

A 2.5 mL suspension of W2 strain of *P. falciparum* at the trophozoite stage was dispensed into 25 cm^2 flasks at 10 % parasitemia and 5.0 % hematocrit. Then, 10 μL of drugs previously dissolved in DMSO and suspended in RPMI were dispensed into the respective flasks for a final drug concentration of 50 nM. After incubation for 4 or 8 h, cells were centrifuged twice.

2.14 Transmission electron microscopy (TEM)

Cells were fixed with 2 % formaldehyde and 2.5 % glutaraldehyde (Electron Microscopy Sciences, Hatfield, PA) in sodium cacodylate buffer (0.1 M, pH 7.2) for 1 h at room temperature and then stored at 4°C until sample processing. Cells were washed 3 times with cacodylate buffer, post-fixed with a 2.0 % solution of osmium tetroxide (Sigma-Aldrich). Cells were subsequently dehydrated in increasing concentrations of acetone (30, 50, 70, 90 and 100 %) for 10 min at each step and embedded in Polybed resin (PolyScience family, Warrington, PA). Ultrathin sections on copper grids were contrasted with uranyl acetate and lead citrate. Micrographs were taken using a JEM-1230 microscope (JEOL, Peabody, MA). Only erythrocytes infected with one single parasite per cell were photographed. Ultra-structural alterations were analyzed from blind randomly chosen micrographs for each group and at least 20 micrographs were taken from each group.

2.15 Animals for malaria infection and pharmacokinetics

Male Swiss-Webster and C57BL/6 mice (18–22 g) were housed at Instituto Gonçalo Moniz (Fiocruz Bahia, Brazil), maintained in sterilized cages under a controlled environment, receiving a rodent balanced diet and water *ad libitum*. All experiments were carried out in accordance with the

recommendations of Ethical Issues Guidelines in Brazil and were approved by the Animal Ethics Committee of Fiocruz Bahia (protocol numbers 002/2016 and 014/2018). All institutional and national guidelines for the care and use of laboratory animals were followed.

2.16 Flow cytometry determination of *in vivo* parasitemia

A 10 µL volume of infected blood red cells (RBC) was collected from the tail vain of *P. berghei*-infected mice, placed in heparin coated vials, gently homogenized and then fixed with 0.5% (v/v) glutaraldehyde in sterile saline for 30 min. at 4 °C. After centrifugation, supernatant was discarded, cells suspended in saline and maintained at 4 °C until analysis. A volume of 100 µL of pancreatic RNAse (1 mg/mL, Sigma) was added and incubated for 15 min. at 37 °C, stained with 100 µL of SYTO-61 (5 µM, Life Technology), and samples were analyzed by flow cytometry (LSRFortessa, BD). At least 200,000 events were acquired in the APC channel (488, 585 nm) for SYTO-61. The analysis was performed using FlowJo (LLC), where for gating procedure, a selection of all RBC was determined based on their scatter property in an FSC/SSC dotplot. For comparison to uninfected and infected unstained RBC, a second gate for infected RBC was selected by plotting the FSC against APC fluorescent intensity signal.

2.17 *In vivo* activity (Peters test)

Male Swiss mice (18-22 g) were infected by intraperitoneal injection of 1×10^6 NK65 strain *P. berghei*-infected erythrocytes per mouse and randomly divided into groups of *n*=5. Each compound was solubilized in DMSO/saline (10:90 v/v) prior to administration. Treatment was initiated 3 h post infection and given once a day for four consecutive days by intraperitoneal injection of 100 µL. Untreated, infected mice receiving DMSO/saline (10:90 v/v) were used as negative controls (vehicle). Parasitemia was counted by flow cytometry at 4, 5, 6 and 7 days post-infection, animal survival was observed daily until day 30 post-infection. The % of parasitemia reduction was calculated as follow:

[mean vehicle group − mean treated group/mean vehicle group] × 100. Experiments were independently repeated for drug doses which inhibited > 80 % the parasitemia.

2.18 *In vivo* activity (Thompson test)

Male Swiss mice (18-22 g) were infected by intraperitoneal injection of 2×10^6 NK65 strain *P. berghei*-infected erythrocytes. At day 3 post-infection, mice with parasitemia up 2 % were randomly divided into groups of *n*=6. Each drug was solubilized in DMSO/saline (10:90 v/v) and treatment was initiated at day 3 post infection and given daily for three consecutive days by intraperitoneal injection of 100 µL at dose of 26 µmol/kg/day. Untreated infected mice receiving DMSO/saline were used as a negative control (vehicle). The following parameters were evaluated: parasitemia determined at days 8, 14 and 16 post-infection and animal survival at 30 days post-infection. Two independent experiments were performed.

2.19 Quantification of hemozoin in the blood of *P. berghei*-infected mice

P. berghei-infected mice with NK65 strain (*n*=4/group) at 8 day post-infection (parasitemia up 10 %) received a single dose of each compound, mefloquine (MQ), Pt(**3**) or Pt(Cl_2) (**15**) at 26 µmol/kg via intraperitoneal route. At 24 h post treatment, smears were obtained from tail blood, fixed with glutaraldehyde and stained with ProLong Glass Antifade Mounting with NucBlue Stain (Invitrogen). Nuclei were visualized in a DAPI channel and hemozoin crystals were visualized by reflection contrast polarized light microscopy (DMi8 S inverted microscope, Leica, Germany). Anesthesia was performed 24 h post treatment and blood samples were taken by cardiac bleed. Cell pellets were then washed, the supernatant was discarded, and the pellets were resuspended in a lysing solution (1.0% w/v saponin). The resulting suspension was centrifuged, the supernatant was discarded, and the pellets were washed twice with phosphate buffered solution (PBS). Parasite-derived heme species (hemoglobin, free heme, and hemozoin) were determined by successive fractionation assays in a 96-well plate format using a

standard method.[5] The relative amount of each species was determined by summing all three species (hemoglobin, free heme, and hemozoin).

2.20 Parasite harvesting for ICP-MS, TEM and EDXS

P. berghei-infected mice with NK65 strain (*n*=2/group) at 8 day post-infection (parasitemia up 10 %) received a single dose of each compound (Pt 3 or Pt 15 complexes at 26 μmol/kg) via intraperitoneal route. Anesthesia was performed 4 h post treatment, blood samples were taken by cardiac bleed. After centrifugation twice, white cells were removed out by cellulose filtration, parasitized red blood cells (pRBCs) were enriched using Ficoll-paque, and pRBCs were adjusted at $1x10^8$ cells/mL. Samples were fixed for TEM and EDXS analyses. In another experiment in parallel, blood from *P. berghei*-infected mice was diluted in RPMI medium, washed twice followed by white cell removal by cellulose filtration. Cell pellets were adjusted to 8 % parasitemia and 2.5 % hematocrit and dispensed in 12-well plates. Then, 10 μL of drugs previously dissolved in DMSO and suspended in RPMI were dispensed in the respective wells for final drug concentration of 1000 nM. After incubation at 37 ^oC for 6 h, plates were washed, pRBCs were adjusted to $1x10^8$ cells/mL and stored at -80 ^oC for ICP-MS analysis and then processed as described below. Cell suspension was centrifuged, and pellets were fixed as described above for TEM and EDSX analyzes.

2.21 Energy-Dispersive X-ray (EDXS) spectroscopy

Cells were fixed and dehydrated as described above and then embedded in Polybed epoxy resin (Polysciences; Warrington, PA). Ultrathin sections coated with carbon were examined under a JEM-1230 TEM integrated with an EDXS microanalytics system (JEOL USA, Inc., Peabody, MA, USA). EDXS spectrum of each micrograph was recorded from blind, randomly chosen groups and at least 20 micrographs and spectra were recorded, and the EDXS microanalysis of platinum (given as % of platinum weight) were collected.

2.22 Pharmacokinetics

Uninfected male C57BL/6 mice (20-25 g) were randomly divided in *n*=4/group and treated with a single dose of Pt (**3**) (88 μmol/kg) by intraperitoneal injection of 200 μL. After anesthesia (tribromoethanol, 200 mg/kg, intraperitoneal), blood was gently aspirated in the brachial plexus using a heparin coated tip and transferred into heparinized vials on ice at time intervals of 1, 3, 6, 12, 24 and 48 h after treatment. The blood samples were centrifuged at 2500 rpm at 4 °C for 15 min., supernatant plasma was transferred into 1.5 mL microcentrifuge tubes. A volume of 400 μL of blood cells were transferred to another microcentrifuge tube, washed three times with PBS 1x, supernatant was discarded, hematocrit was adjusted and a 200 μL of cell pellets were stored at −80 °C until analysis. The amount of protein in the whole plasma and blood samples were determined by Bradford protein assay. In another experiment in parallel, fractionating the plasma in protein fractions (referred here as protein-bound) and protein-free fractions (referred here as unbound) was performed using a centrifugal filter device (3 kDa size exclusion, Amicon, Merck) at 14,000 x *g* for 45 min. Both fractions were maintained at −80 °C until analysis. One single experiment was performed.

2.23 Quantification of platinum by ICP-MS analysis

For sample digestion, Milli-Q Element water purification system (Merck Millipore, Bedford, MA, USA) was used for the preparation of ultrapure water (resistivity ≥ 18.2 MΩ cm at 25 °C). The samples were thawed minutes before carrying out the digestion. The solution for digestion was composed of an alkaline solution prepared by mixing 4% v v^{-1} *n*-butanol, 1% m v^{-1} ammonium hydroxide, 0.1% m/v ethylenediaminetetraacetic acid, and 0.05% v v^{-1} Triton X-100. Ultrafiltrate plasma (100 μL) was transferred into sterile 15 mL falcon tube diluted 20-fold in alkaline solution. Addition and recovery experiment was performed at the addition level of 20 μg L^{-1}. All determinations of platinum were conducted by monitoring the ^{195}Pt signal on an inductively coupled plasma mass spectrometry ICP-

MS (Agilent 7800) equipped with a concentric nebulizer and a Scott double pass spray chamber. The Pt standard solution used for ICP-MS calibrations was prepared by diluting 1000 mg L^{-1} of Pt (Qhemis, São Paulo, SP, Brazil) in 0.14 mol L^{-1} HNO_3 medium and Ir used as internal standard. The analytical solutions for calibration were prepared in the alkaline solution contained from 0, 5.0, 10.0, 20.0, 60.0, 100,0 to 200.0 μg L^{-1} of platinum and the internal standard was added at 5.0 μg L^{-1} to analytical calibration solutions, analytical blanks and samples.

2.24 Enzyme preparation and activity determination

Recombinant *Sm*TGR and human TrxR1 were produced as described.[6,7] *P. falciparum* TrxR (*Pf*TrxR) with an *N*-terminal 6-His tag was synthesized and inserted into pET15b (GenScript) and expressed in BL21 (DE3) cells and purified as described.[8] *Sm*TGR, hTrxR1 and *Pf*TrxR enzyme inhibition assays were performed in triplicates at 25 °C as described.[7] In brief, assays were carried out in 0.1 M potassium phosphate (pH 7.4), 10 mM EDTA, 100 μM NADPH and 0.01% Tween-20. *Sm*TGR and hTrxR1 (4 nM) and *Pf*TrxR (50 nM) were preincubated with the compounds for 15 mins. The reaction was started with addition of an equal volume of DTNB (6 mM) and NADPH (100 μM) and the increase in A_{412} during the first 3 mins was recorded. The IC_{50} values were calculated in GraphPad Prism.

2.25 Evaluation of schistosomicidal activity

2.26 Preparation of newly transformed schistosomula (NTS)

Strain NMR of *Biomphalaria glabrata* infected with *S. mansoni* were provided by the NIAID Schistosomiasis Resource Center for distribution through BEI Resources, NIAID, NIH (USA). After infections were patent, snails were exposed to bright light for 1 h to obtain the cercariae. Then, cercariae were mechanically transformed into schistosomula.[7,8] Briefly, cercariae were placed on ice for 30 min and then centrifuged at 350 × *g* for 10 mins. The supernatant was decanted and a 2 mL of serum-free M199 medium was added to cercarial pellets and vortexed for 1 min until cercarial tails

were detached. The NTS were purified by layering on 4 °C Percoll gradient suspension containing Eagle's minimum essential medium, penicillin-streptomycin (10,000 U per ml penicillin/10,000 U per ml streptomycin), and 1 M HEPES in 0.85% NaCl with cercariae suspension and centrifuged at 500 × g for 15 min. Cercarial pellets were resuspended and washed thrice in serum-free M199 medium and collected at 100 × *g* for 5 mins. NTS (240) were transferred to U-bottom 96 well assay plates containing 200 µl of M199 medium supplemented with 5.5 mM D-glucose, penicillin-streptomycin and 5% heat inactivated fetal bovine serum and incubated at 37 °C in a 5% CO_2 incubator overnight.

2.27 Preparation of adult *S. mansoni* worms

Ex-vivo schistosome experiments involving animals were conducted at the Rush University Medical Center (Chicago, USA) and were approved by the Institutional Animal Care and Use Committee of the Rush University Medical Center (Department of Health and Human Services animal welfare, assurance number A-3120 − 01) with protocol ID: 20-069. Three-week old, female Swiss-Webster mice obtained from the Charles River were housed in the Comparative Research Center of Rush University Medical Center. Mice were infected by percutaneous tail exposure to about 200 *S. mansoni* cercariae for adult worms through natural transdermal penetration of the cercariae for 1 h. Mice were euthanized seven-weeks post infection for adult worm using a lethal dose of 0.018 ml of Euthasol and 5.85 mg/ml heparin to prevent blood coagulation (injection volume of 400 µL). Perfusion was performed by flushing pre-warmed RPMI containing phenol red and L-glutamine through a 25- and 3/8-gauge needle placed into the aorta attached to Tygon tubing aided by the Masterflex L/S perfusion pump as described.[9,10] Adult worms were carefully washed in phenol red free RPMI medium and subsequently incubated in phenol red free RPMI medium supplemented with 5.5 mM D-glucose, penicillin-streptomycin and 5% heat inactivated fetal bovine serum and at 37 °C in a 5% CO_2 incubator overnight.

2.28 Schistosomicidal activity of compounds against NTS and adult worms

DMSO formulated compounds were diluted with phenol red free M199 medium or RPMI medium for NTS and adult worms, respectively, at <1 % DMSO final concentrations. NTS and adult worms from overnight cultures were tested against compounds. Each concentration was tested in triplicate. Controls were treated with DMSO alone or 5 μM auranofin (AF) as a positive control in appropriate medium. Worm viability was assessed at 24 h by measuring ATP content of worms using CellTiterGlo Assay (Promega) as previously described.[7,8] Schistosome viabilities in the presence of the compounds were assessed using this formula: % viability = averages of test / averages of DMSO control x 100.

2.29 TRFS-green fluorescence quantification for TGR inhibition in worms

Selected compounds were assessed for TGR inhibition in NTS using a fluorescent probe TRFS-green (MedchemExpress).[11] NTS were prepared as previously described[7] and cultured in M199 supplemented with 5.5 mM D-glucose, penicillin-streptomycin and 5% heat inactivated fetal bovine serum were incubated at 37 °C in a clear bottom flat well plate in a 5% CO_2 incubator overnight. To inhibit TGR activity, NTS were treated with compounds (30 μM) or auranofin (5 μM) for 2 h. NTS were further treated with TRFS-green (10 μM) for additional 4 h and rinsed with M199 medium to remove residual TRFS-green. Fluorescence images were obtained using Keyence BZ-X800 and analyzed with ImageJ.

***2.30 In vitro* metabolic stability assay in microsomes**

In vitro microsomal stability assay was performed in duplicate in a 96-well microtiter plate, using a single-point experiment design.[12] Test compounds (1.0 μM) were incubated individually in mouse (pool of 1,634, male CD1) liver microsomes (Xenotech, Kansas, USA) to a final protein concentration of 0.4 mg/mL, suspended in 0.1 M phosphate buffer (pH 7.4). Incubations were started by the addition of NADPH (1 mM) as a cofactor and shaken for 30 min at 37°C. The reactions were quenched by the

addition of 300 μL of ice-cold acetonitrile containing an internal standard (carbamazepine, 0.0236 μg/mL) and centrifuged. The supernatant was transferred to a fresh 96-well plate and analyzed by liquid chromatography-tandem mass spectrometry (LC-MS/MS) (Agilent Rapid Resolution HPLC, AB SCIEX 4500 MS). The relative loss of the parent compound over time was monitored, and plots (concentration vs time) were prepared for each compound to determine the first-order rate constant for compound depletion. This was in turn used to calculate half-life, *in vitro* intrinsic clearance (Cl_{int}), and *in vivo* hepatic extraction ratio.[13]

2.31 Statistical analyses

Nonlinear regression analysis was used to calculate CC_{50} and IC_{50} values by using GraphPad Prism version 8.40 (Graph Pad Software, San Diego, CA). Each method was specified in the indicated experiments. It was considered statistically significant when $p < 0.05$ as analyzed by GraphPad Prism version 8.40.

References

[1] Butera V, D'Anna L, Rubino S, Bonsignore R, Spinello A, Terenzi A, Barone G. How the Metal Ion Affects the ^{1}H NMR Chemical Shift Values of Schiff Base Metal Complexes: Rationalization by DFT Calculations. J Phys Chem A. 2023 Nov 9;127(44):9283-9290. doi: 10.1021/acs.jpca.3c05653. Epub 2023 Oct 31. PMID: 37906682; PMCID: PMC10641838.

[2] Makler MT, Hinrichs DJ. Measurement of the lactate dehydrogenase activity of *Plasmodium falciparum* as an assessment of parasitemia. Am J Trop Med Hyg. 1993 Feb;48(2):205-10. doi: 10.4269/ajtmh.1993.48.205. PMID: 8447524.

[3] de Souza GE, Bueno RV, de Souza JO, Zanini CL, Cruz FC, Oliva G, Guido RVC, Aguiar ACC. Antiplasmodial profile of selected compounds from Malaria Box: in vitro evaluation, speed of action and drug combination studies. Malar J. 2019 Dec 30;18(1):447. doi: 10.1186/s12936-019-3069-3. PMID: 31888654; PMCID: PMC6938011.

[4] Macedo TS, Villarreal W, Couto CC, Moreira DRM, Navarro M, Machado M, Prudêncio M, Batista AA, Soares MBP. Platinum(ii)-chloroquine complexes are antimalarial agents against blood and liver stages by impairing mitochondrial function. Metallomics. 2017 Nov 15;9(11):1548-1561. doi: 10.1039/c7mt00196g. PMID: 28960224.

[5] Combrinck JM, Fong KY, Gibhard L, Smith PJ, Wright DW, Egan TJ. Optimization of a multi-well colorimetric assay to determine haem species in *Plasmodium falciparum* in the presence of anti-malarials. Malar J. 2015 Jun 24;14:253. doi: 10.1186/s12936-015-0729-9. PMID: 26099266; PMCID: PMC4484700.

[6] Cheng Q, Arnér ES. Selenocysteine Insertion at a Predefined UAG Codon in a Release Factor 1 (RF1)-depleted *Escherichia coli* Host Strain Bypasses Species Barriers in Recombinant Selenoprotein Translation. J Biol Chem. 2017 Mar 31;292(13):5476-5487. doi: 10.1074/jbc.M117.776310. Epub 2017 Feb 13. PMID: 28193838; PMCID: PMC5392690.

[7] Lyu H, Petukhov PA, Banta PR, Jadhav A, Lea WA, Cheng Q, Arnér ESJ, Simeonov A, Thatcher GRJ, Angelucci F, Williams DL. Characterization of Lead Compounds Targeting the Selenoprotein Thioredoxin Glutathione Reductase for Treatment of Schistosomiasis. ACS Infect Dis. 2020 Mar 13;6(3):393-405. doi: 10.1021/acsinfecdis.9b00354. Epub 2020 Jan 24. PMID: 31939288; PMCID: PMC7072008.

[8] Petukhova VZ, Aboagye SY, Ardini M, Lullo RP, Fata F, Byrne ME, Gabriele F, Martin LM, Harding LNM, Gone V, Dangi B, Lantvit DD, Nikolic D, Ippoliti R, Effantin G, Ling WL, Johnson JJ, Thatcher GRJ, Angelucci F, Williams DL, Petukhov PA. Non-covalent inhibitors of thioredoxin glutathione reductase with schistosomicidal activity in vivo. Nat Commun. 2023 Jun 22;14(1):3737. doi: 10.1038/s41467-023-39444-y. PMID: 37349300; PMCID: PMC10287695.

[9] Kuntz AN, Davioud-Charvet E, Sayed AA, Califf LL, Dessolin J, Arnér ES, Williams DL. Thioredoxin glutathione reductase from *Schistosoma mansoni*: an essential parasite enzyme and a key drug target. PLoS Med. 2007 Jun;4(6):e206. doi: 10.1371/journal.pmed.0040206. Erratum in: PLoS Med. 2007 Aug;4(8):e264. PMID: 17579510; PMCID: PMC1892040.

[10] Tucker MS, Karunaratne LB, Lewis FA, Freitas TC, Liang YS. Schistosomiasis. Curr Protoc Immunol. 2013 Nov 18;103:19.1.1-19.1.58. doi: 10.1002/0471142735.im1901s103. PMID: 24510597.

[11] Zhang L, Duan D, Liu Y, Ge C, Cui X, Sun J, Fang J. Highly selective off-on fluorescent probe for imaging thioredoxin reductase in living cells. J Am Chem Soc. 2014 Jan 8;136(1):226-33. doi: 10.1021/ja408792k. Epub 2013 Dec 18. PMID: 24351040.

[12] Di L, Kerns EH, Gao N, Li SQ, Huang Y, Bourassa JL, Huryn DM. Experimental design on single-time-point high-throughput microsomal stability assay. J Pharm Sci. 2004 Jun;93(6):1537-44. doi: 10.1002/jps.20076. PMID: 15124211.

[13] Obach RS. Prediction of human clearance of twenty-nine drugs from hepatic microsomal intrinsic clearance data: An examination of in vitro half-life approach and nonspecific binding to microsomes. Drug Metab. Dispos. 1999 Nov;27(11):1350-9. PMID: 10534321.
